# Capture of Singlet Oxygen Modulates Host‐Guest Behavior of Coordination Cages

**DOI:** 10.1002/anie.202309589

**Published:** 2023-08-23

**Authors:** Ilma Jahović, Yuchong Yang, Tanya K. Ronson, Jonathan R. Nitschke

**Affiliations:** ^1^ Department of Chemistry University of Cambridge Lensfield Road CB2 1EW Cambridge UK

**Keywords:** Anthracene, Chemical Separation, Metal-Organic Cage, Singlet Oxygen

## Abstract

The anthracene panels of two tetrahedral M^II^_4_L_6_ cages, where M^II^=Co^II^ or Fe^II^, were found to react with photogenerated singlet oxygen (^1^O_2_) in a hetero‐Diels–Alder reaction. ESI‐MS analysis showed the cobalt(II) cages to undergo complete transformation of all anthracene panels into endoperoxides, whereas the iron(II) congeners underwent incomplete conversion. The reaction was found to be partially reversible in the case of the **1‐Fe^II^** cage. The dioxygen‐cage cycloadducts were found to bind a set of guest molecules more weakly than the parent cages, with affinity dropping by more than two orders of magnitude in some cases. The light‐driven cycloaddition reaction between cage and ^1^O_2_ thus served as a stimulus for guest release and reuptake.

## Graphical abstract

A range of anthracene‐based tetrahedral capsules have been shown to react with photogenerated singlet oxygen in a hetero‐Diels–Alder reaction. The cage cycloadducts were found to bind an array of guest molecules more weakly than their precursor cages, offering a novel method for stimuli‐driven guest uptake and release.

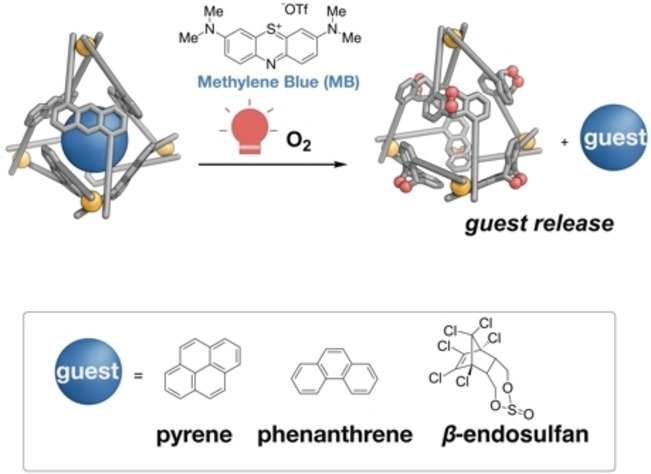

Self‐assembled molecular containers have been used for a range of applications, including molecular sensing,^[1]^ catalysis,^[2]^ and chemical separations.^[3]^ Post‐assembly modification (PAM) of these supramolecular structures has proven a useful strategy to impart the new functionalities needed for these applications,^[4]^ circumventing the need to design and synthesize additional cage components. Chemical transformations utilized for PAM have included reactions such as Diels–Alder^[5]^ and inverse electron‐demand Diels–Alder cycloadditions,^[6]^ olefin metathesis,^[7]^ acylation,^[8]^ and nucleophile‐isocyanate coupling.^[9]^ The development of new PAM reactions is a worthwhile goal to expand the scope of cage applications.

For PAM to work, a cage should contain a subunit that can react to change the structure of the cage upon application of an appropriate stimulus. One such motif is anthracene, which stacks with planar compounds due to its extended π‐surface, potentially leading to strong van der Waals interactions with guests.^[10]^ Because of its photophysical properties, anthracene has been incorporated into many supramolecular structures, including metal‐organic frameworks,^[11]^ metal‐organic cages,^[12]^ and polymers.^[13]^

Anthracene is also known to react with singlet oxygen (^1^O_2_) in a [4+2] hetero‐Diels–Alder reaction.^[14]^ ^1^O_2_ is a stimulus that has been used as an oxidation agent in synthetic organic chemistry,^[15]^ nanoscience^[16]^ and photodynamic therapy for cancer treatment.^[17]^ Zhang^[18]^ and Crowley^[19]^ employed different cages to catalyse the photo‐oxidation of anthracene guests. This reactivity of anthracene has been exploited by Stang and co‐workers to construct a metallacycle that reversibly reacts with ^1^O_2_.^[20]^ Shionoya and co‐workers have also made elegant use of this phenomenon to design metallo‐macrocycles that have different reactivities and binding affinities in the solid and solution states.^[21]^ The reactivity of an anthracene unit in an organic cage has also been exploited by Bibal and co‐workers to tune its Na^+^ and Cs^+^ binding properties.^[22]^ Moreover, this reaction has been also explored by our group to regulate different self‐sorting metal‐organic cage systems.^[23]^

In this work we explore the reactivity of ^1^O_2_ with a set of anthracene‐edged M^II^_4_L_6_ (M^II^=Co^II^, Fe^II^) three‐dimensional metal‐organic capsules in solution. Structural changes following cycloaddition strongly impacted host–guest chemistry of the capsules. Interestingly, the choice of metal was found to affect the extent of cycloaddition: iron(II) cages were found to incorporate fewer equivalents of ^1^O_2_ than their cobalt(II) congeners. Following singlet‐oxygen addition, under specific conditions, the modified cages were also observed to thermally revert back to their precursor structures, thus allowing reversible modulation of their host–guest binding properties.

Isomeric anthracene‐edged tetrahedral cages **1‐M^II^** and **2‐M^II^** (Figure 1) were prepared following published procedures.[^7^, ^24^] The reactions of both the cobalt(II) and iron(II) cages with ^1^O_2_ were investigated. Cages with two spacers, 1,5‐anthracene and 9,10‐anthracene, were studied because we hypothesized that different spacer geometries would lead to varying degrees of surface enclosure. The 1,5‐anthracene cages were predicted to be more enclosed, thus leading to stronger binding of more guests. An acetonitrile solution of each cage and the photosensitizer methylene blue (MB, 0.05 equiv.) was irradiated with red light (λ_max_=630 nm) for 2 hours at room temperature (Figure 1) under air. The photosensitizer and any byproducts were then removed using preparative size‐exclusion chromatography (SEC). The electrospray ionization mass spectra (ESI‐MS) of cobalt(II) cages **3‐Co^II^** and **4‐Co^II^** indicated incorporation of six equivalents of ^1^O_2_ per cage, and thus complete reaction of the six anthracene ligands (Figure 3a). In contrast, ESI‐MS analysis of the corresponding iron(II) cages (Figure 3b) indicated the presence of a mixture of cages containing between one and six endoperoxidized anthracene ligands; we designate these mixtures as **3‐Fe^II^** and **4‐Fe^II^**. The cobalt(II) cages thus underwent complete cycloaddition, whereas their iron(II) homologs did not.

**Figure 1:**
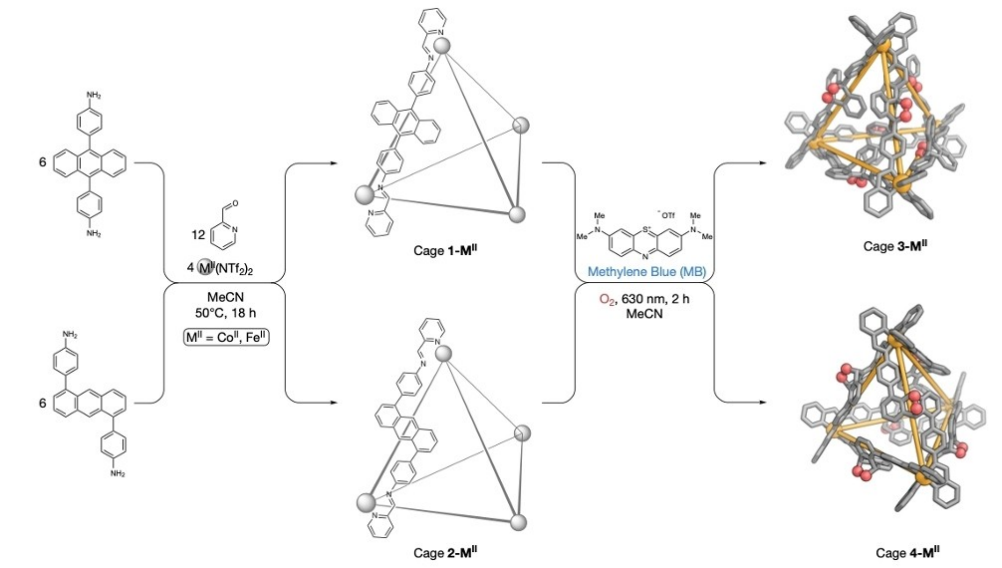
Subcomponent self‐assembly of cages **1‐M^II^** and **2‐M^II^** (where **M^II^** =**Co^II^** or **Fe^II^**), and their hetero‐Diels–Alder reactions with ^1^O_2_ to produce the modified cages **3‐M^II^**, **4‐M^II^**, respectively; **3‐Fe^II^** and **4‐Fe^II^** were produced as mixtures containing from 1 to 6 endoperoxide moieties per cage.

The ^1^H NMR spectra of the resulting cobalt(II) cages (Schemes S1 and S2, Figures S25–S27) **3‐Co^II^** and **4‐Co^II^**, produced from **1‐Co^II^** and **2‐Co^II^**, respectively, were intractably complex, which we attributed to the presence of multiple isomers in solution resulting from endoperoxide formation taking place both on the exterior and interior faces of the anthracene ligands. Similarly, we observed broadening of the ^1^H signals of **1‐Fe^II^** and **2‐Fe^II^** after treatment with ^1^O_2_, which we inferred to result from the presence of multiple products and diastereomers in solution. As multiple attempts to grow X‐ray quality crystals were unsuccessful, we used MM3 molecular modelling using SCIGRESS^[25]^ to visualize cage configurations with peroxo moities bound to the anthracene ligands. Figure 2 shows MM3 models of cages containing anthracene panels with endoperoxide moities oriented both inside and outside of the cage, consistent with the observed broadening of the ^1^H NMR spectra.

**Figure 2:**
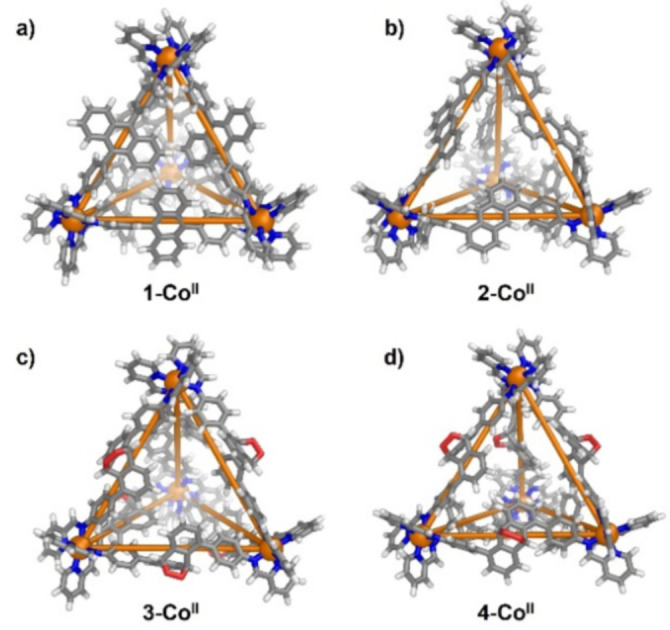
MM3 model of a) **1‐Co^II^**; b) **2‐Co^II^**; c) **3‐Co^II^**; d) **4‐Co^II^**; (Co: orange, N: blue, O: red, C: gray, H: white).

To probe whether complete conversion of the iron(II) cages might occur, we investigated different reaction conditions, including longer reaction times, different cage concentrations, and the use of a pure oxygen atmosphere. None of these factors significantly influenced the extent of reaction. A mixture of endoperoxidized anthracene products was obtained under all investigated conditions. Similarly incomplete conversion of endoperoxidized anthracene subunits has been also reported for anthracene based metallo‐macrocycles.^[21]^

The more limited reactivity of the iron(II) cages may result from the presence of a metal‐to‐ligand charge‐transfer (MLCT) band in the 500–650 nm range, which overlaps with the absorption band of MB. The low solubility of other photosensitizers in acetonitrile precluded us from testing their effects on photooxygenation. Stronger metal‐ligand bonding in the case of Fe^II^ may also progressively incorporate greater strain into the cage frameworks as more anthracene panels react, thus disfavoring further cycloaddition.

Changes in the ultraviolet/visible (UV/Vis) spectra of **1‐Co^II^** and **2‐Co^II^** further confirmed quantitative cycloaddition upon conversion to **3‐Co^II^** and **4‐Co^II^**, respectively, following treatment with ^1^O_2_. The UV/Vis spectra before reaction feature an absorption band at 350–400 nm, corresponding to the *π*→π* transition characteristic of anthracene moieties (Figure 3c). Irradiation of the cage in the presence of MB caused this feature to disappear, consistent with transformation of the anthracene moieties into endoperoxides. Partial disappearance of this anthracene band was also observed in the cases of the iron(II) cages, confirming incomplete reaction.

**Figure 3:**
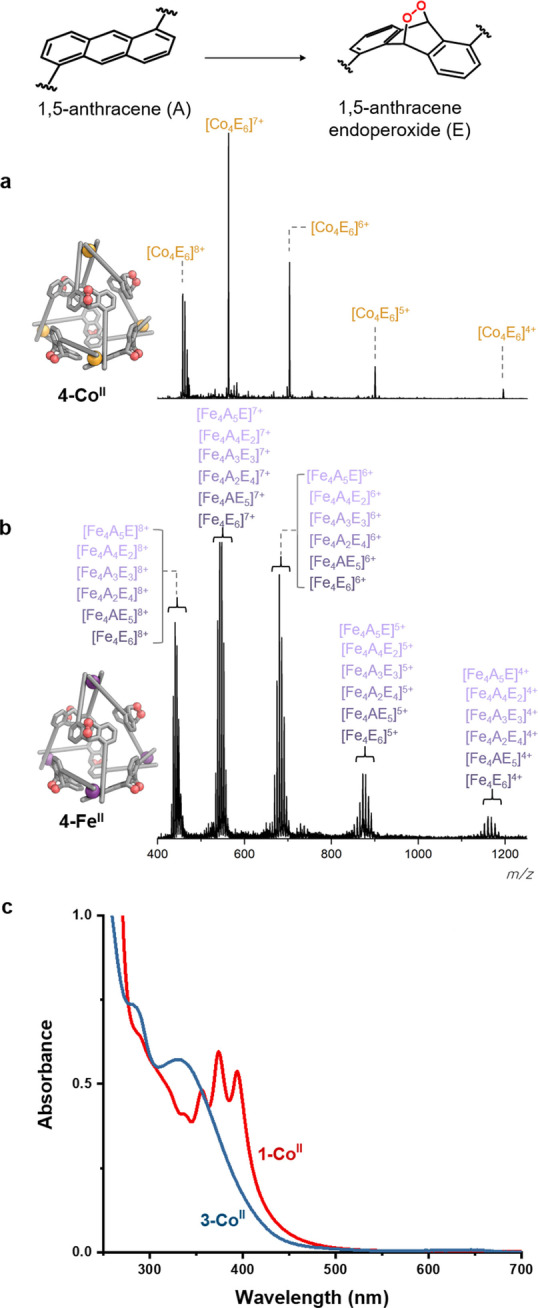
Electrospray mass spectra (MeCN) of cages (a) **4‐Co^II^** and (b) **4‐Fe^II^** following reaction with ^1^O_2_, confirming the presence of a single product for Co^II^, and a mixture of cycloadducts for Fe^II^. UV/Visible spectra of cages **1‐Co^II^** and **3‐Co^II^** after the reaction (c).

As the cycloaddition reaction between ^1^O_2_ and anthracene is thermally reversible,^[26]^ we hypothesized that heating cages that contain the endoperoxide moiety would result in regeneration of the parent anthracene cages. Heating an acetonitrile solution of **3‐Fe^II^** at 50 °C for three days resulted in the recovery of the majority of the precursor cage, as evidenced by ^1^H NMR and ESI‐MS (Figures S28 and S38). However, we did not observe recovery of precursor cage **2‐Fe^II^** from **4‐Fe^II^** through heating in solution. Heating **3‐Co^II^** and **4‐Co^II^** under identical conditions likewise did not result in cycloreversion. Increasing the temperature, or using a higher‐boiling point solvent such as benzonitrile or nitrobenzene, resulted in decomposition of **3‐Co^II^** and **4‐Co^II^**, as evidenced by ESI‐MS.

Inspired by a previously published method used for an organoplatinum(II) metallacycle,^[20]^ we explored thermolysis in the solid state as an alternative strategy for regenerating **1‐Co^II^** from **3‐Co^II^**. Heating a solid sample of endoperoxide cage **3‐Fe^II^**, **4‐Co^II^**, or **3‐Co^II^** to 80 °C under a dynamic vacuum resulted in the onset of retro‐cycloaddition, as indicated by mass spectra. Although dioxygen extrusion continued up to 160 °C (Figures S39–S41), it was not complete for any of the three cages at this temperature, where cage decomposition was also observed to compete with retro‐cycloaddition. We also did not observe the thermal extrusion of dioxygen from **4‐Fe^II^** in the solid state.

We hypothesized that the presence of the endoperoxide moieties would affect the shape and the size of the cage cavities. Given that the transformation occurred reversibly, we postulated that it could lead to controlled guest uptake and release.^[27]^ Due to the difficulty in deconvoluting the guest binding abilities of the iron(II) cages with differing numbers of endoperoxide groups, we examined the shifting ^1^H NMR signals of both cages and guest molecules to confirm guest binding, as shown in Figures S69–S74.

Solution‐state ^1^H NMR analysis was complicated by the paramagnetic character of the cobalt(II) hosts, which resulted in broad and overlapping ^1^H NMR signals (Figures S25–S27). Hence, we also used isothermal titration calorimetry (ITC) to measure the change in thermodynamic parameters of host–guest interactions as a result of endoperoxide formation. Therefore, we not only examined the shifting of guest ^1^H NMR signals to confirm guest binding, but also employed ITC to establish the presence of host–guest interactions of the two cages following endoperoxide formation.

The encapsulation of pyrene, phenanthrene and *β*‐endosulfan within cages **1‐Co^II^**, **2‐Co^II^**, **3‐Co^II^**, and **4‐Co^II^** was examined by ITC in acetonitrile. In all cases, it was found that the binding constants (*K*_a_) were lower following the reaction of the cages with ^1^O_2_ (Table S1). We observed a significant change in binding affinities before and after reaction of the anthracene panels with ^1^O_2_; in some cases, guest affinities differed by more than two orders of magnitude. For instance, *β*‐endosulfan bound to **1‐Co^II^** with *K*_a_=8.4±0.82×10^3^ M^−1^, decreasing to 21±11 M^−1^ for the corresponding endoperoxide cage **3‐Co^II^**. Similarly, the first association constant (K_*a1*_) for the binding of pyrene was found to be 630±72 M^−1^ for **2‐Co^II^**, versus less than 1.0 M^−1^ for **4‐Co^II^**.

Differences in binding were also observed between the two precursor hosts, **1‐Co^II^** and **2‐Co^II^**, where **1‐Co^II^** bound phenanthrene and *β*‐endosulfan more strongly than did **2‐Co^II^**, but not pyrene; the same trend was also observed between the corresponding modified assemblies **3‐Co^II^** and **4‐Co^II^**. We attribute this difference to the greater degree of surface enclosure afforded by the 1,5‐anthracene moiety. By contrast, the 9,10‐linkage in **1‐Co^II^** and **3‐Co^II^** leads to the rotation of the anthracene unit, such that it projects into the cage cavity and thus impedes the binding of an external molecule.

The bending of the anthracene panels following cycloaddition is inferred to lead to poorer stacking with planar guest molecules, thus reducing guest binding affinities. For example, two equivalents of pyrene fit well within the cavity of **2‐Co^II^** as an anthracene‐pyrene‐pyrene‐anthracene stack.^[12c]^ Following the transformation of the cage to **4‐Co^II^**, the bend in the anthracene ligands no longer provides a favorable conformation for the four‐component stack.

To examine guest release in solution, we designed experiments in which three different guests were initially bound within **2‐Co^II^**, after which ^1^O_2_ was photogenerated in the presence of the host–guest complex (Figure 4). We observed in the ^1^H NMR spectra (Figures S66–S68) that all three molecular cargoes were encapsulated and then released when a suitable stimulus was applied.

**Figure 4:**
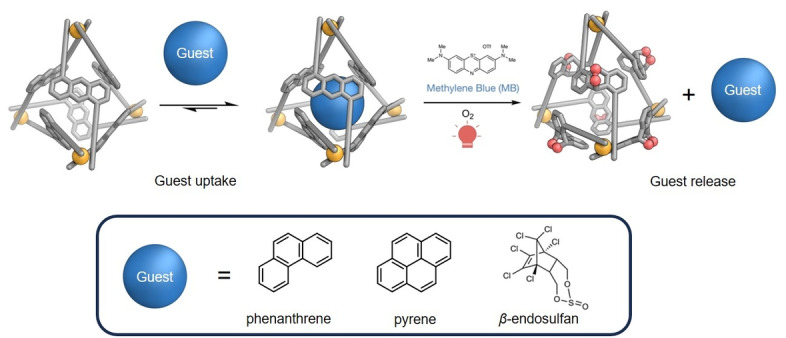
The release of a bound guest may be stimulated by the reaction of the anthracene panels of a cage (**2‐Co** ^II^ is shown) with photo‐generated ^1^O_2_.

Considering that **3‐Fe^II^** could revert to **1‐Fe^II^** in solution, we also explored the reversible re‐uptake of the guest pyrene when **3‐Fe^II^** reverted to **1‐Fe^II^**. Although the reversibility of the conversion of **3‐Fe^II^** to **1‐Fe^II^** was not complete, we were still able to observe the reversible encapsulation of pyrene in the regenerated cage **1‐Fe^II^** (Figure S75). We conclude, thus, that the difference in guest affinity between the precursor and modified cages may be used to effect reversible guest release and re‐uptake.

The ability to use a single external stimulus to reversibly fine tune the binding of industrially‐relevant molecules such as pyrene, phenanthrene and *β*‐endosulfan could serve as the basis for new methods of chemical separation.^[3d]^ The immobilization of cages analogous to **2‐Co^II^** on solid supports^[28]^ or in gels^[29]^ could enable the creation of new materials that take up, and then release, specific molecules from mixtures.

## Conflict of interest

The authors declare no conflict of interest.

## Supporting information

As a service to our authors and readers, this journal provides supporting information supplied by the authors. Such materials are peer reviewed and may be re‐organized for online delivery, but are not copy‐edited or typeset. Technical support issues arising from supporting information (other than missing files) should be addressed to the authors.

Supporting Information

## Data Availability

The data that support the findings of this study are available in the supplementary material of this article.
