# Supplementary material for: Capture of Singlet Oxygen Modulates Host‐Guest Behavior of Coordination Cages
Source: Angew Chem Int Ed Engl. 2023 Aug 23;62(39):e202309589. doi: 10.1002/anie.202309589 (PMC10952966; doi:10.1002/anie.202309589)
Supplement: Supplementary file 1 — Supporting Information [file ANIE-62-0-s001.pdf]

Angewandte Chemie
Eine Zeitschrift der Gesellschaft Deutscher Chemiker

Supporting Information

Capture of Singlet Oxygen Modulates Host-Guest Behavior of Coordination Cages

*I. Jahović, Y. Yang, T. K. Ronson, J. R. Nitschke**

Content

S1. General methods *3*

S2. Synthesis and characterization of precursor and modified cages *5*

S2.1. Synthesis of precursor cages 1-Co^{II} and 2-Co^{II} **5**

S2.2. Synthesis of modified cages **6**

S2.3. NMR spectroscopy data of (1-Fe^{II} to 4-Fe^{II} and 1-Co^{II} to 4-Co^{II}) **7**

S2.3.1 Characterization of 1-Fe^{II} **7**

S2.3.2 Characterization of 2-Fe^{II} **10**

S2.3.3 Characterization of 3-Fe^{II} **13**

S2.3.4 Characterization of 4-Fe^{II} **16**

S2.3.5 ^{1}H NMR spectra of 1-Co^{II} and 3-Co^{II} **19**

S2.3.6 ^{1}H NMR spectra of 2-Co^{II} and 4-Co^{II} **20**

S2.3.7 ^{1}H NMR spectra of 1-Fe^{II} and 3-Fe^{II} **21**

S2.3.8 ^{1}H NMR spectra of 2-Fe^{II} and 4-Fe^{II} **21**

S2.3.9 DOSY NMR spectra of 1-Fe^{II}, 2-Fe^{II}, 3-Fe^{II} and 4-Fe^{II} **22**

S2.4. Low-resolution ESI-mass spectrometry data **23**

S2.5. High-resolution mass spectrometry data **31**

S2.6. UV-visible spectroscopy data **36**

S3. Host-guest chemistry experiments *38*

S3.1. Summary of ITC results **38**

S3.2. Titration plots for host 1-Co^{II} **39**

S3.3. Titration plots for host 2-Co^{II} **42**

S3.4. Titration plots for host 3-Co^{II} **45**

S3.5. Titration plots for host 4-Co^{II} **48**

S3.6. Uptake and release of guests **51**

S4. References *61*

S1. General Methods

Reagents. Unless otherwise stated, all chemicals were purchased from commercial sources and used without further purification.

NMR Spectroscopy. ^{1}H NMR and ^{13}C NMR solution-state spectra were recorded at 298 K on a Bruker Avance III HD 400 MHz Nanobay, a Bruker Avance III HD 400 MHz Smart Probe or a Bruker Avance III HD 500 MHz Smart Probe spectrometer. Spectra were referenced relative to the residual non-deuterated solvents used as internal standards (^{1}H: 2.50 ppm for DMSO-d_6 and 1.94 ppm for CD_3CN; ^{13}C: 39.52 ppm for DMSO-d_6, and 118.26 ppm for CD_3CN). Chemical shifts are reported in ppm.

Mass Spectrometry. ESI-MS for the metal-organic species was recorded on a Micromass Quattro LC mass spectrometer with a flow rate of 4.0 µL/s. Cone voltage used was in the 5—15 V range.

Electrospray-ionization quadrupole-time-of-flight high-resolution mass spectrometric (ESI-Q-TOFHRMS) experiments were performed with a Synapt G3-S HDMS, Waters Co., Milfod, MA, USA. The flow rate was set to 20 µL/min and the spray voltage to 3.3 kV. The parameters for spray voltage, sample cone and extractor cone voltages were optimized for maximum abundances of the desired complex ions.

UV-Visible Spectroscopy. UV-Visible spectroscopy measurements were performed on a Cary 300 UV-Visible spectrophotometer with a 1 mm path length cuvette at 295 K. The solvent used was acetonitrile.

Isothermal Titration Calorimetry (ITC). Titration experiments were carried out in dry MeCN at 298 K on a MicroCal iTC200. To set up an experimental run, a 200 µL solution of the cage was transferred to the sample cell and 40 µL of the solution of a guest molecule in dry MeCN was placed in the syringe. The concentrations used varied between 1-3 mM and 10-60 mM for the cages and guests, respectively. Exact concentrations used are specified underneath each thermogram. The first injection of 0.5 µL was followed by another 19 injections of 2 µL each, with a 120 s spacing between the injections. The solution was stirred at 750 rpm. Heats of dilution were determined in identical experiments, but without cage in the cell and were subtracted from each data set. We titrated a solution of each cage with MeCN and observed negligible heats of dilution evolved. This suggested that it was appropriate to take into account only guest dilution experiments during the normalization procedure. Due to relatively low affinities for the guest molecules, the Wiseman *c*-value[1] was approaching the lowest recommended limit for some of the samples, thus resulting in the low sigmoidicity of the data. In order to resolve this, we carried out titrations at higher concentrations where possible. However, the cages were found to aggregate at higher concentrations and the solutions became viscous and thus unsuitable for the analysis by ITC. Thus, data shown here reflect the experiments that produced the best results given the limitations of systems under study. The data were processed using the Origin software package with the ITC plugin. One set of sites model was used to fit all data except for the experiments involving pyrene binding to cage **1-Co^{II}**. In these instances, we used the sequential binding sites model given that **2-Fe^{II}** has been shown to bind to a stacked dimer of pyrenes.[2] The first injection was disregarded in the fitting due to the ”first injection anomaly.”[3]

Molecular Modeling. Molecular mechanics simulations (MM2 and MM3 force fields) of supramolecular complexes were performed using CAChe WorkSystem Pro (Fujitsu Limited, Beaverton, Oregon, 2000-2006) and SCIGRESS version FJ 2.6 (EU 3.1.9) Build 5996.8255.20141202, Serial No. 1456215255499 (copyright 2008–2016, Fujitsu Limited). Models of the cages **3-Co^{II}** and **4-Co^{II}** were based on the previously published crystal structures of the parent cages **1-Co^{II}** and **2-Co^{II}**, respectively. O_2 moieties were placed such that they are oriented towards the outside of the cage. After the addition of each O_2, energy minimization was performed. It is worth

noting that models are used in this case just to illustrate one of the possible conformations and may not represent a global minimum due to the complexity of the system. Additionally, there many possible conformations for isomers from each set of O_2 pointing inside or outside the cage as well as due to stereochemistry at the metal vertex.

S2. Synthesis and Characterization of Precursor and Modified Cages

S2.1. Synthesis of precursor cages **1-Co^{II}** and **2-Co^{II}**

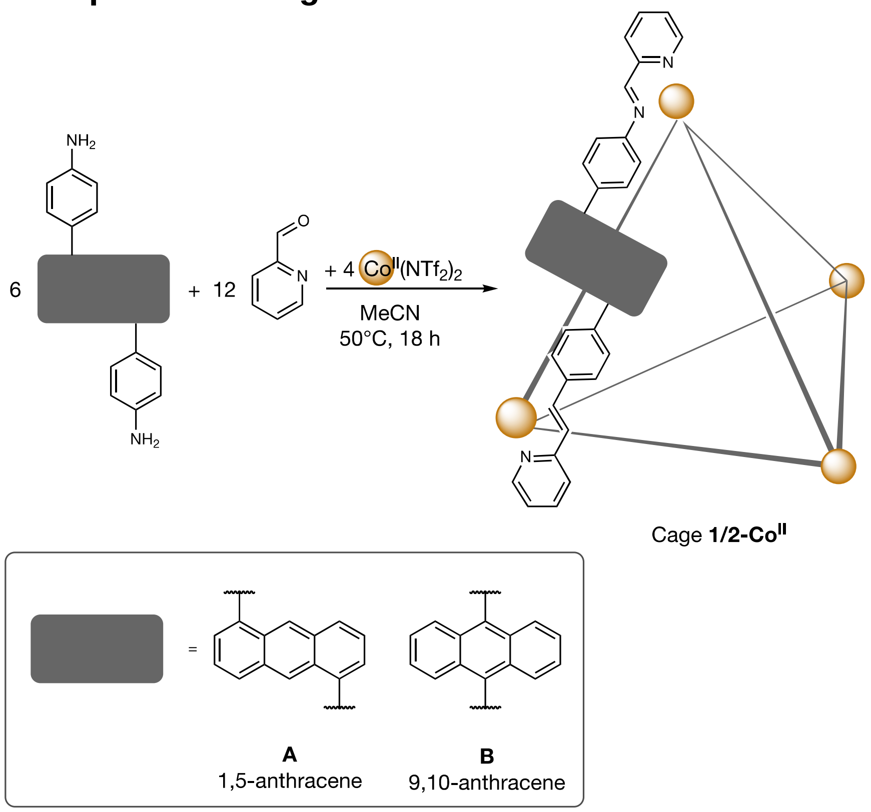

Scheme S1. Synthesis of cages **1-Co^{II}** and **2-Co^{II}**.

Subcomponents **A** and **B** were synthesized according to previously published procedures.[2,4]

Cage **1-Co^{II}**:
Dianiline subcomponent **B** (100.3 mg, 2.78×10^{-4} mol, 6 equiv.) was suspended in acetonitrile (100 mL). $Co(NTf_2)_2\cdot5H_2O$ (148 mg, 1.85×10^{-4} mol, 4 equiv.) was then added. 2-formylpyridine (57.5 μL, 5.55×10^{-4} mol, 12 equiv.) was transferred dropwise and the mixture stirred under nitrogen at 50°C for 18 hours. The volume was reduced to 2 mL *in vacuo* and excess diethyl ether added until an orange solid precipitated. The solid was filtered and washed with diethyl ether three times. Yield: 235 mg, 89%.

Cage **2-Co^{II}**:
The same procedure was used to synthesize cage **2-Co^{II}**.
Dianiline subcomponent **A**: 20.1 mg, 5.58×10^{-5} mol, 6 equiv.; $Co(NTf_2)_2\cdot5H_2O$: 29.6 mg, 3.72×10^{-5} mol, 4 equiv.; 2-formylpyridine: 11.6 μL, 1.12 $\times10^{-4}$ mol, 12 equiv; acetonitrile: 20 mL. Yield: 65.3 mg, 91%.

Cages **1-Fe^{II}** and **2-Fe^{II}** were synthesized according to previously published procedures.[2,4]

S2.2. Synthesis of modified cages

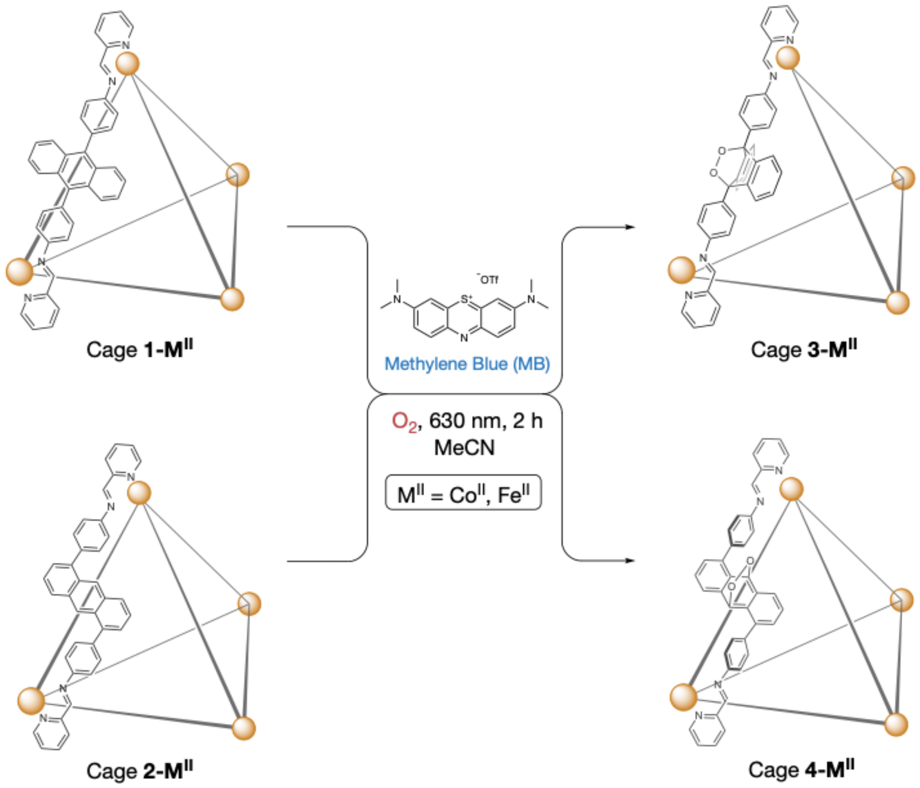

Scheme S2. Hetero-Diels-Alder reactions of cages **1-M^{II}** and **2-M^{II}** with $^1O_2$ to produce modified cages **3-M^{II}** and **4-M^{II}**.

In a typical experiment, methylene blue (MB, 5%) was added to an acetonitrile solution of the cage (40 mg, 15 mL) in a glass vessel equipped with a stir bar and capped with a rubber septum. The solution was stirred at room temperature for 2 hours, while being irradiated with red light using an LED lamp (SMD 5050 RGB, dominant wavelength range: 620-635 nm, λ_{max} = 630 nm). The volume of the reaction mixture was then reduced by rotary evaporation and the product purified by preparative size-exclusion chromatography (Bio-Beads SX1) with an eluent of 1:1 DCM/MeCN. The solvent was then removed under reduced pressure to obtain the final product as an orange (for **3-Co^{II}** and **4-Co^{II}**) or purple (for **3-Fe^{II}** and **4-Fe^{II}**) solid. Yield: 34.1 mg, 85% (for **3-Co^{II}**); Yield: 32.1 mg, 80% (for **4-Co^{II}**); the loss of 15-20% of the cages occurred during the separation procedure using preparative size-exclusion chromatography (SEC). **3-Fe^{II}** and **4-Fe^{II}** were not isolated due to their incomplete conversion under photoirradiation.

S2.3. NMR spectroscopy data of (1-Fe^{II} to 4-Fe^{II} and 1-Co^{II} to 4-Co^{II})

S2.3.1 Characterization of **1-Fe^{II}**

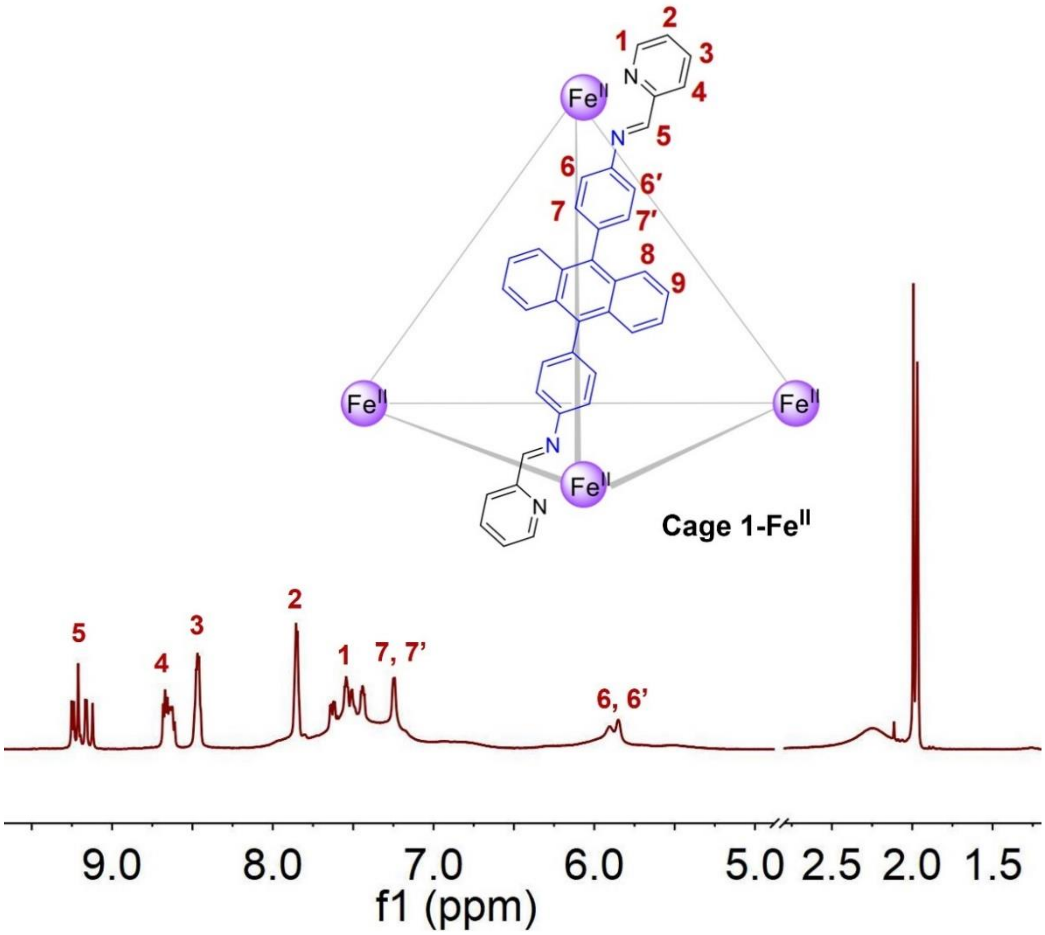

Figure S1. ^{1}H NMR spectrum of **1-Fe^{II}** (500 MHz, CD_3CN, 298 K).

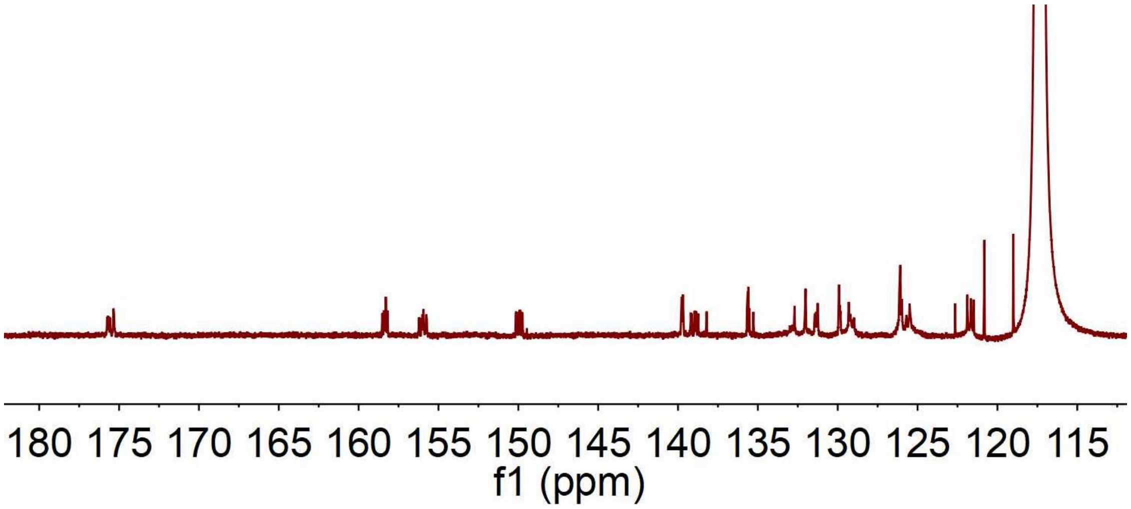

Figure S2. ^{13}C NMR spectrum of **1-Fe^{II}** (126 MHz, CD_3CN, 298 K).

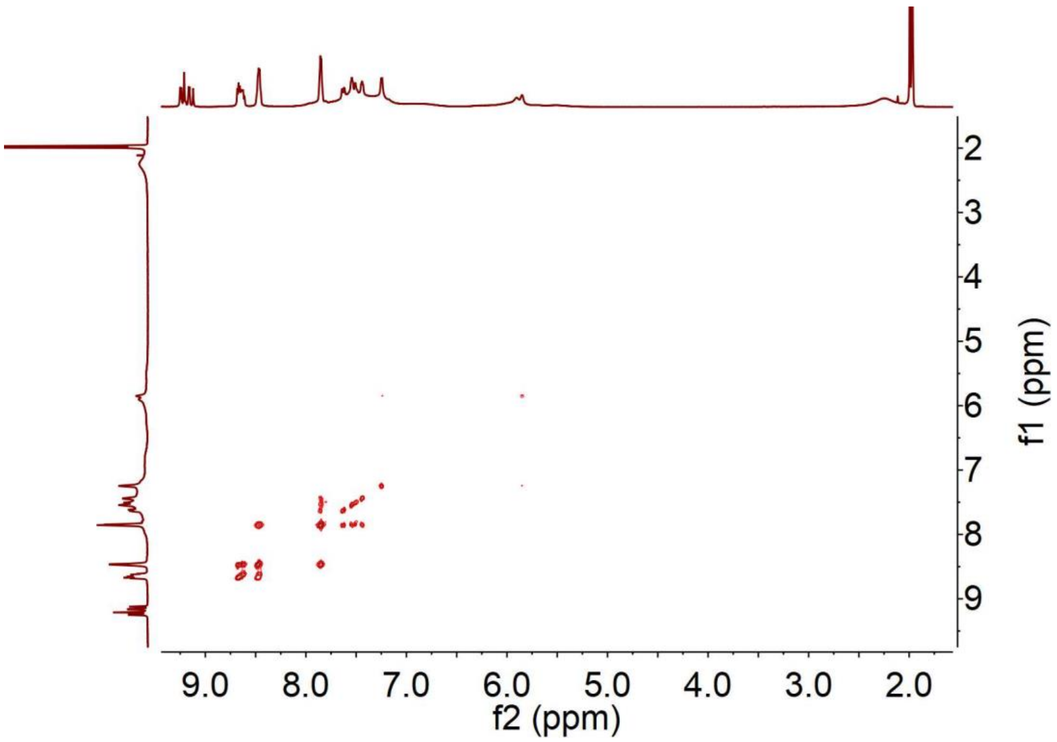

Figure S3. ^{1}H-^{1}H COSY NMR spectrum of **1-Fe^{II}** (500 MHz, CD_3CN, 298 K).

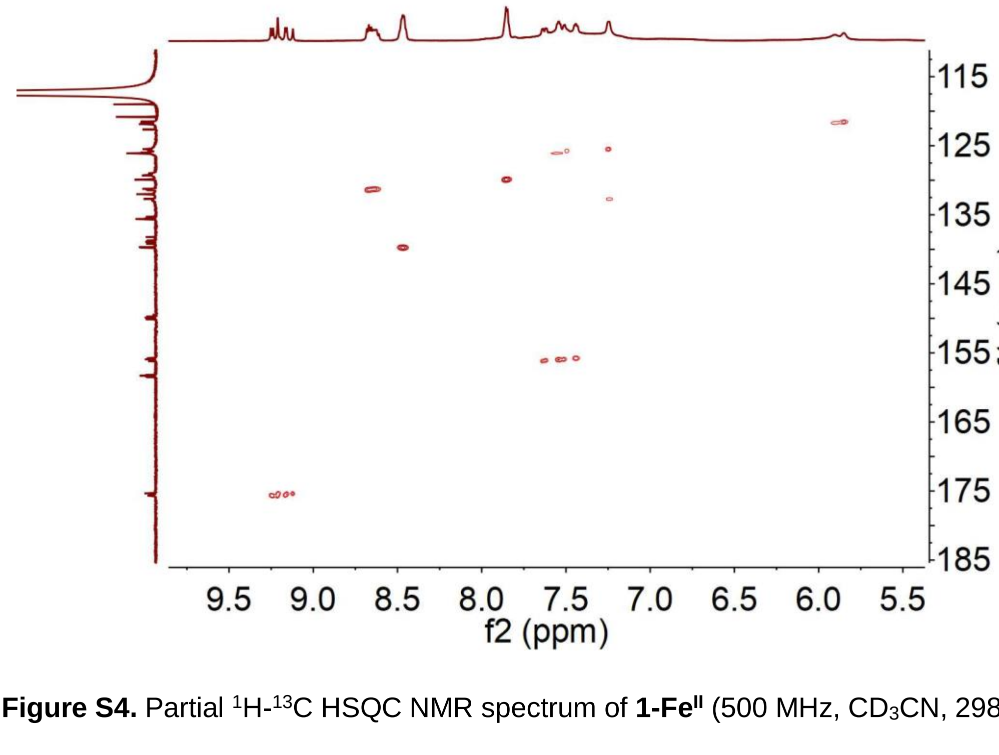

Figure S4. Partial ^{1}H-^{13}C HSQC NMR spectrum of **1-Fe^{II}** (500 MHz, CD_3CN, 298 K).

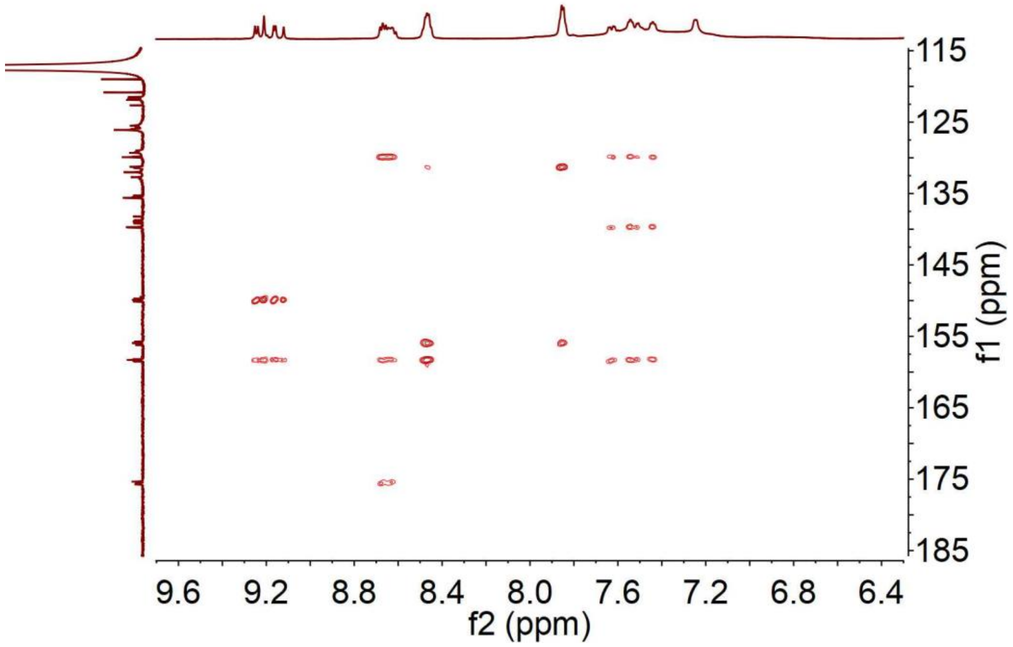

Figure S5. Partial ^{1}H-^{13}C HMBC NMR spectrum of **1-Fe^{II}** (500 MHz, CD_3CN, 298 K).

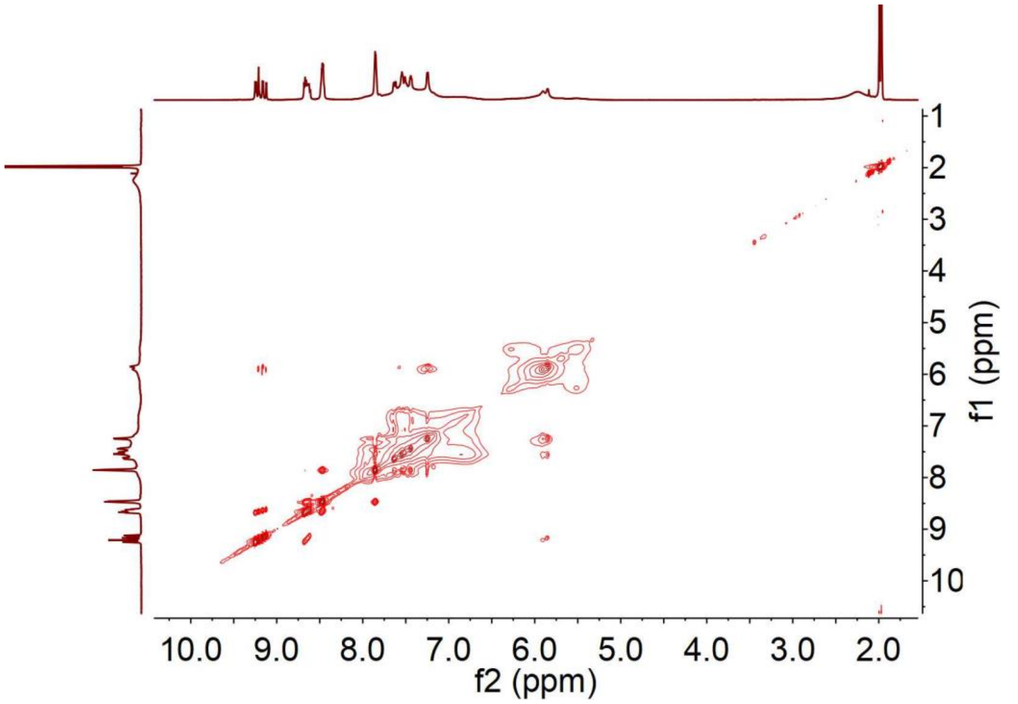

Figure S6. Partial ^{1}H-^{1}H NOESY NMR spectrum of **1-Fe^{II}** (500 MHz, CD_3CN, 298 K).

S2.3.2 Characterization of **2-Fe^II^**

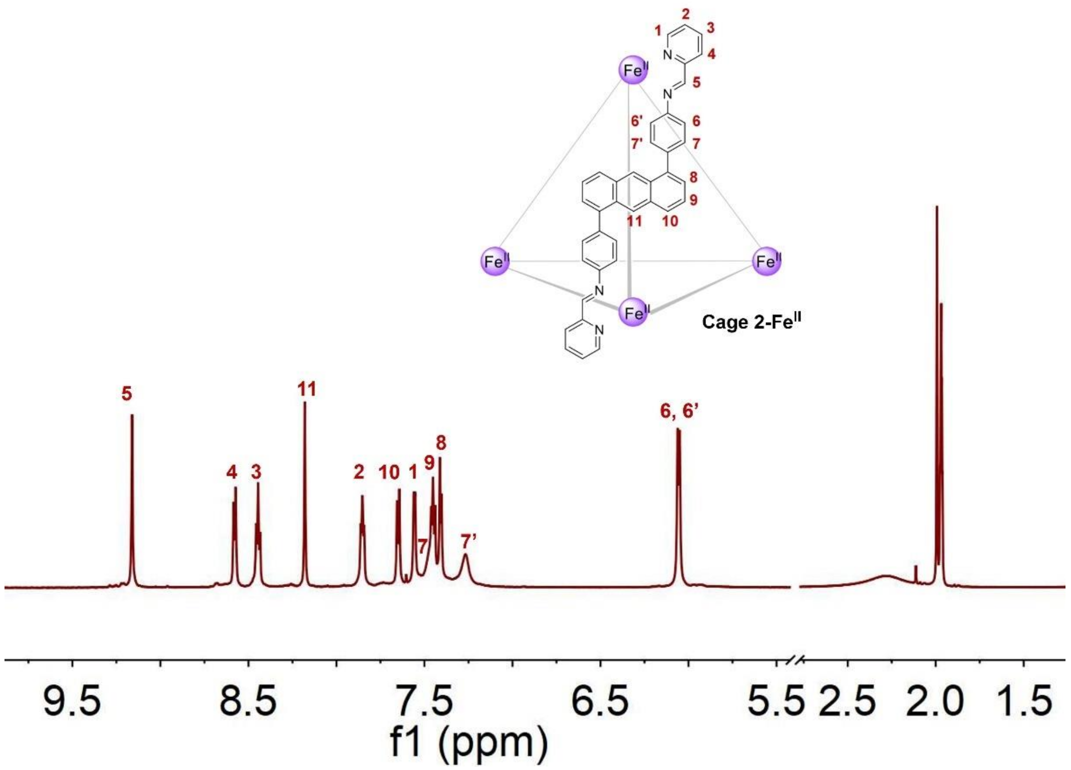

Figure S7. ^{1}H NMR spectrum of **2-Fe^II^** (500 MHz, CD_3CN, 298 K).

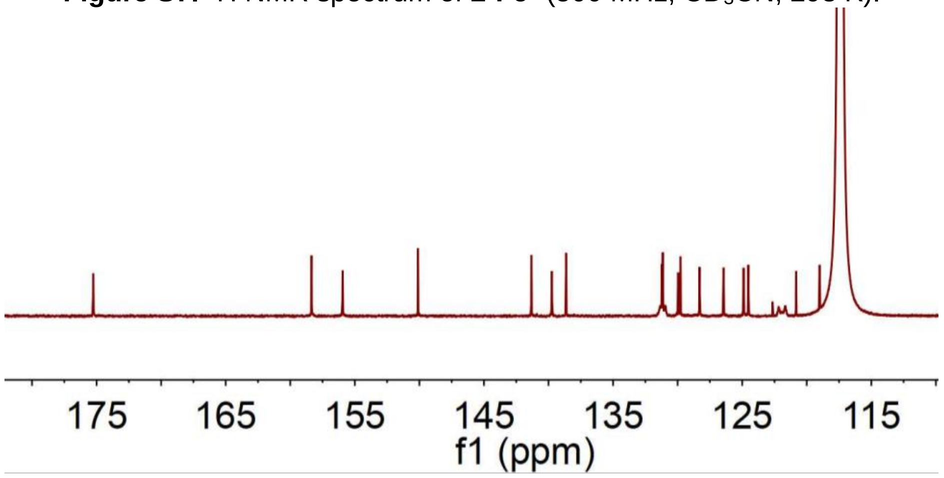

Figure S8. ^{13}C NMR spectrum of **2-Fe^II^** (126 MHz, CD_3CN, 298 K).

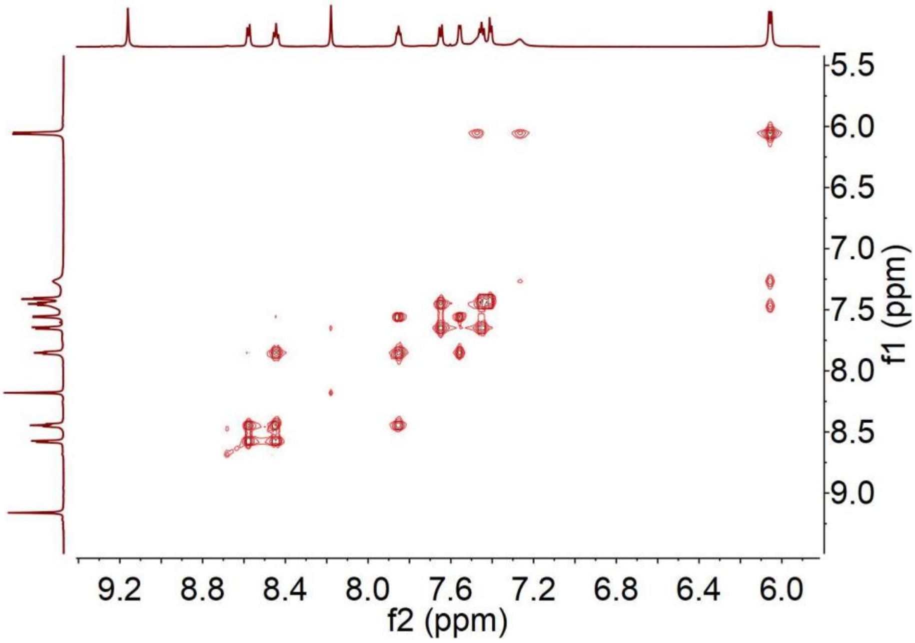

Figure S9. Partial ^{1}H-^{1}H COSY NMR spectrum of **2-Fe^{II}** (500 MHz, CD_3CN, 298 K).

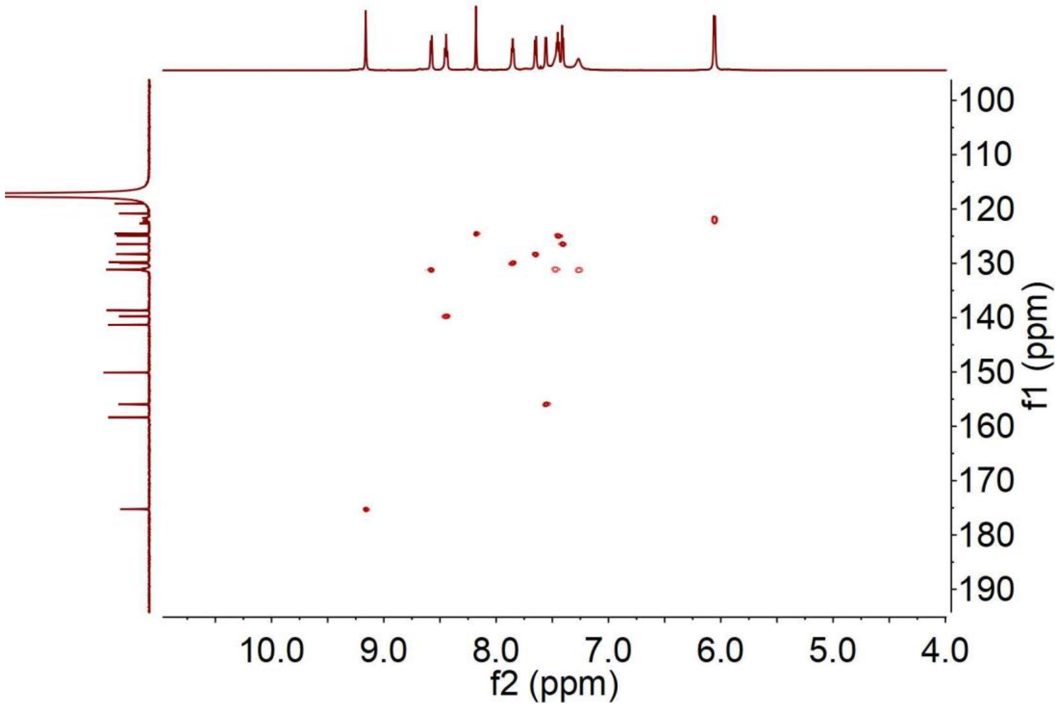

Figure S10. Partial ^{1}H-^{13}C HSQC NMR spectrum of **2-Fe^{II}** (500 MHz, CD_3CN, 298 K).

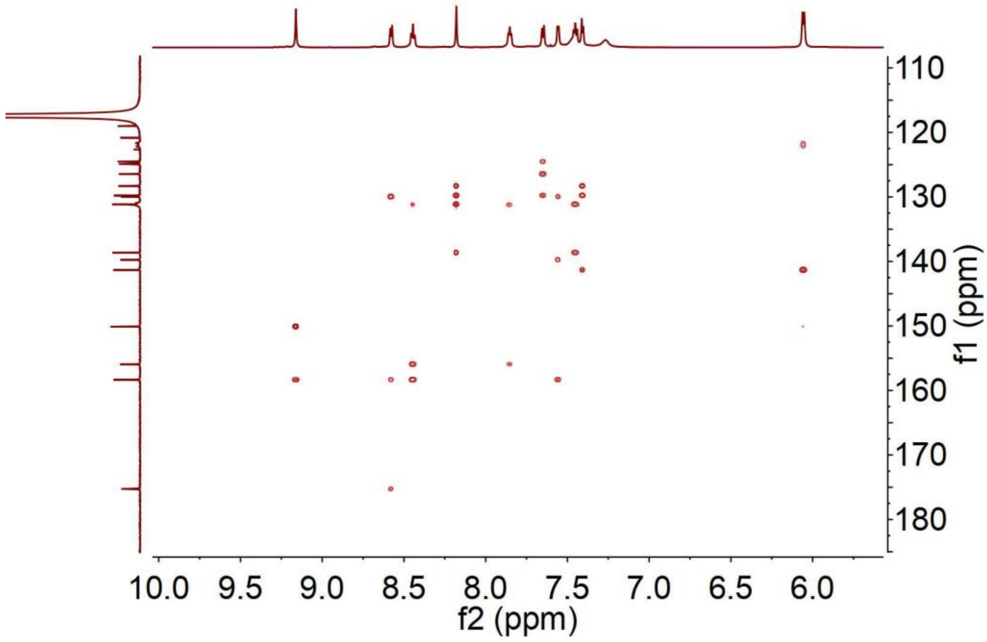

Figure S11. Partial ^{1}H-^{13}C HMBC NMR spectrum of **2-Fe^{II}** (500 MHz, CD_3CN, 298 K).

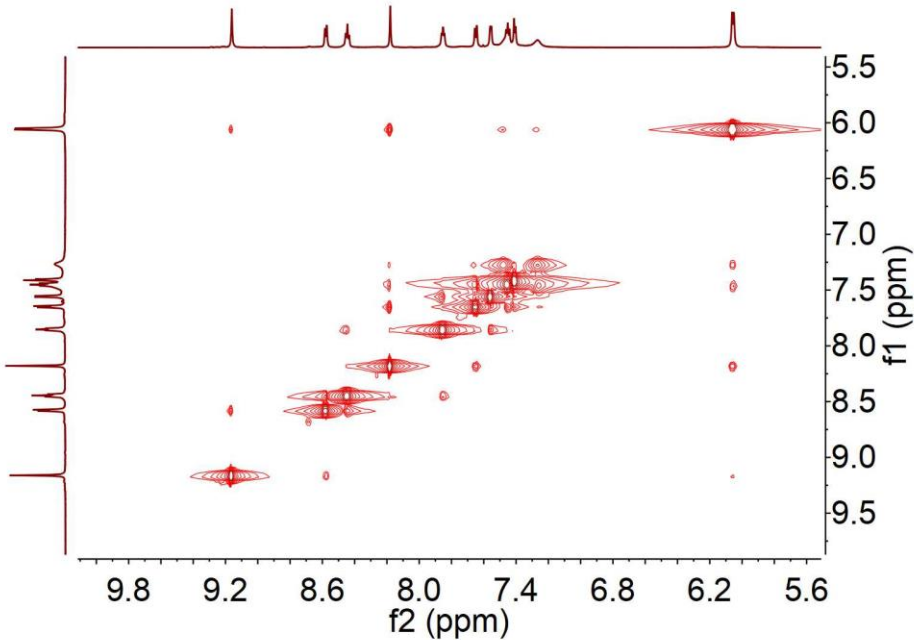

Figure S12. Partial ^{1}H-^{1}H NOESY NMR spectrum of **2-Fe^{II}** (500 MHz, CD_3CN, 298 K).

S2.3.3 Characterization of **3-Fe^{II}**

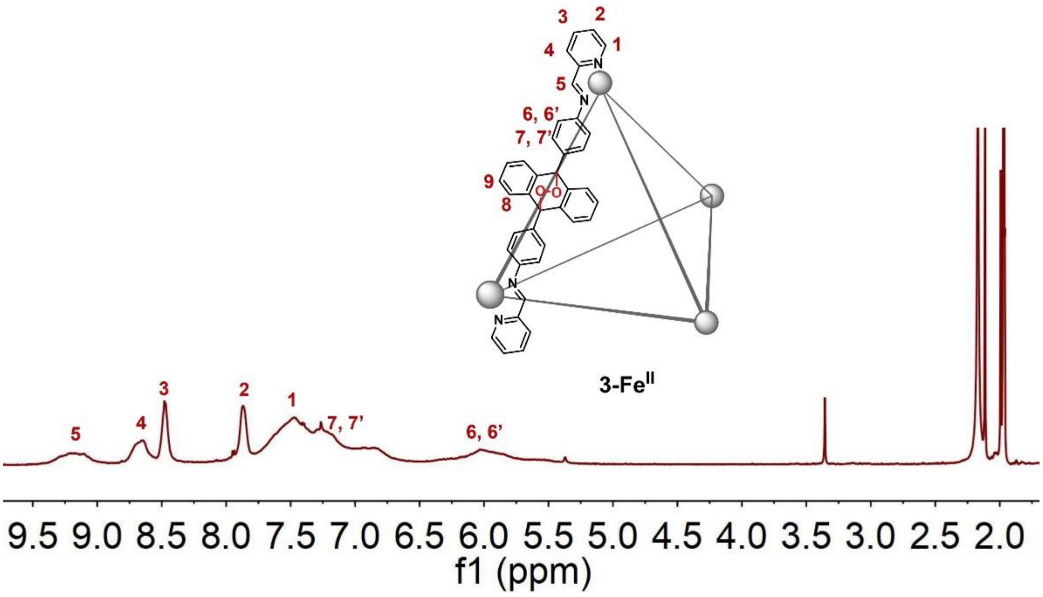

Figure S13. ^{1}H NMR spectrum of **3-Fe^{II}** (500 MHz, CD_3CN, 298 K).

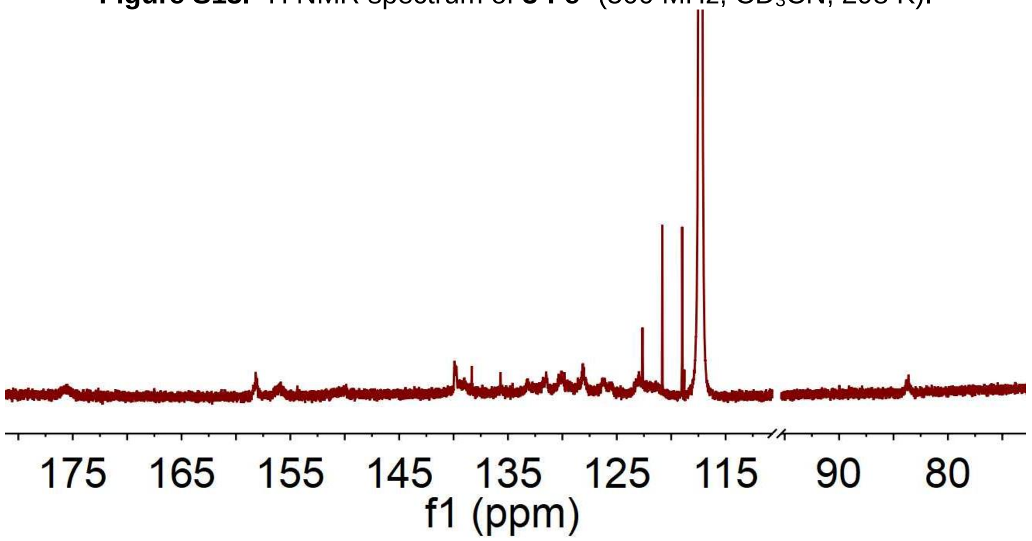

Figure S14. ^{13}C NMR spectrum of **3-Fe^{II}** (126 MHz, CD_3CN, 298 K).

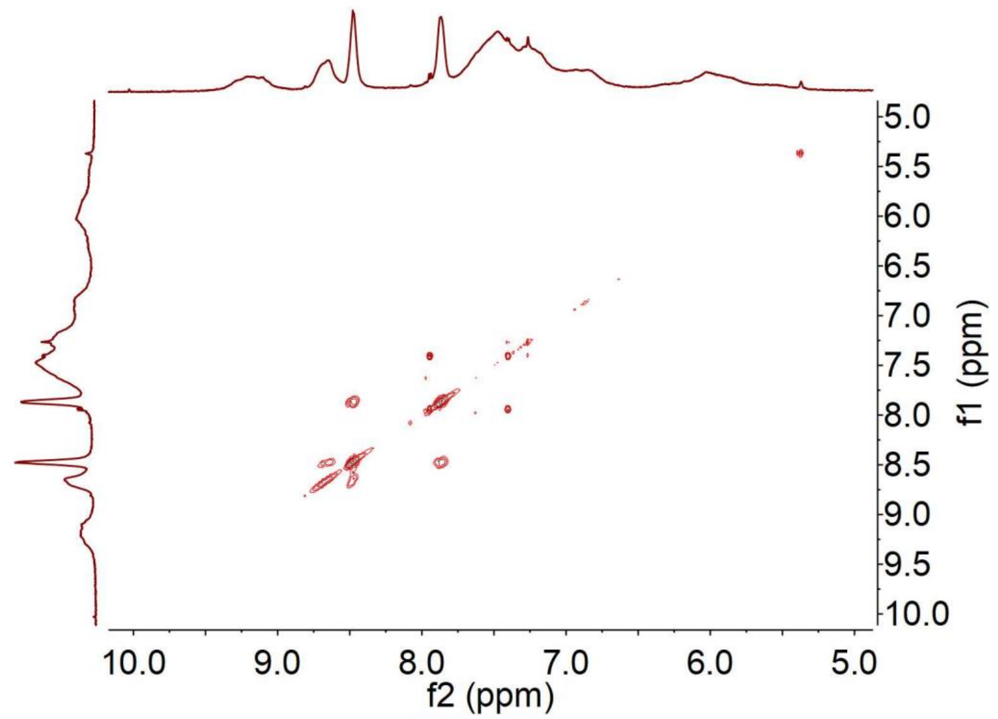

Figure S15. Partial ^{1}H-^{1}H COSY NMR spectrum of **3-Fe^{II}** (500 MHz, CD_3CN, 298 K).

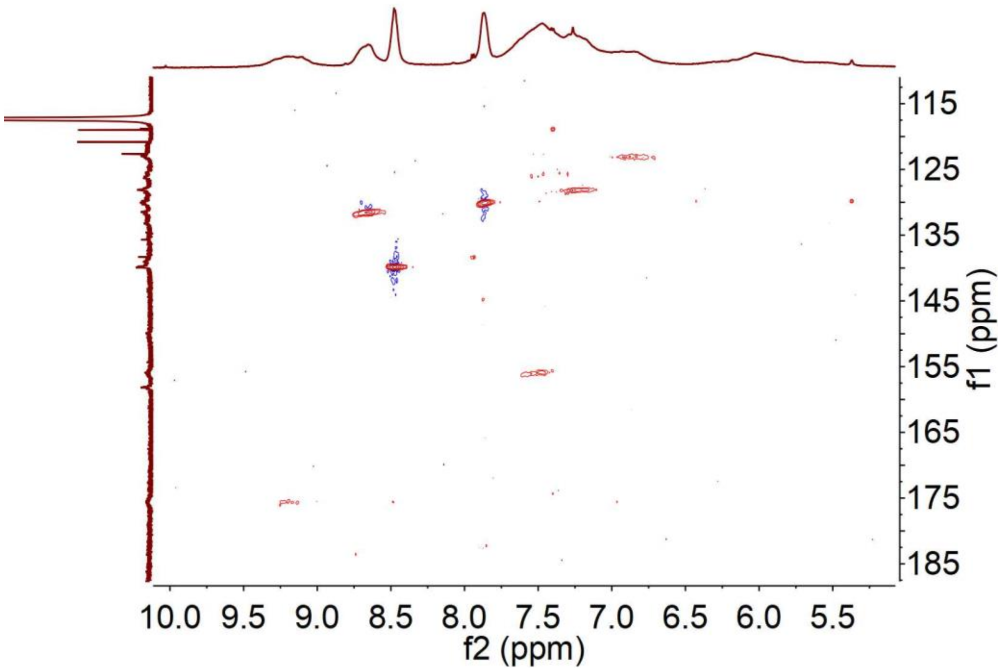

Figure S16. Partial ^{1}H-^{13}C HSQC NMR spectrum of **3-Fe^{II}** (500 MHz, CD_3CN, 298 K).

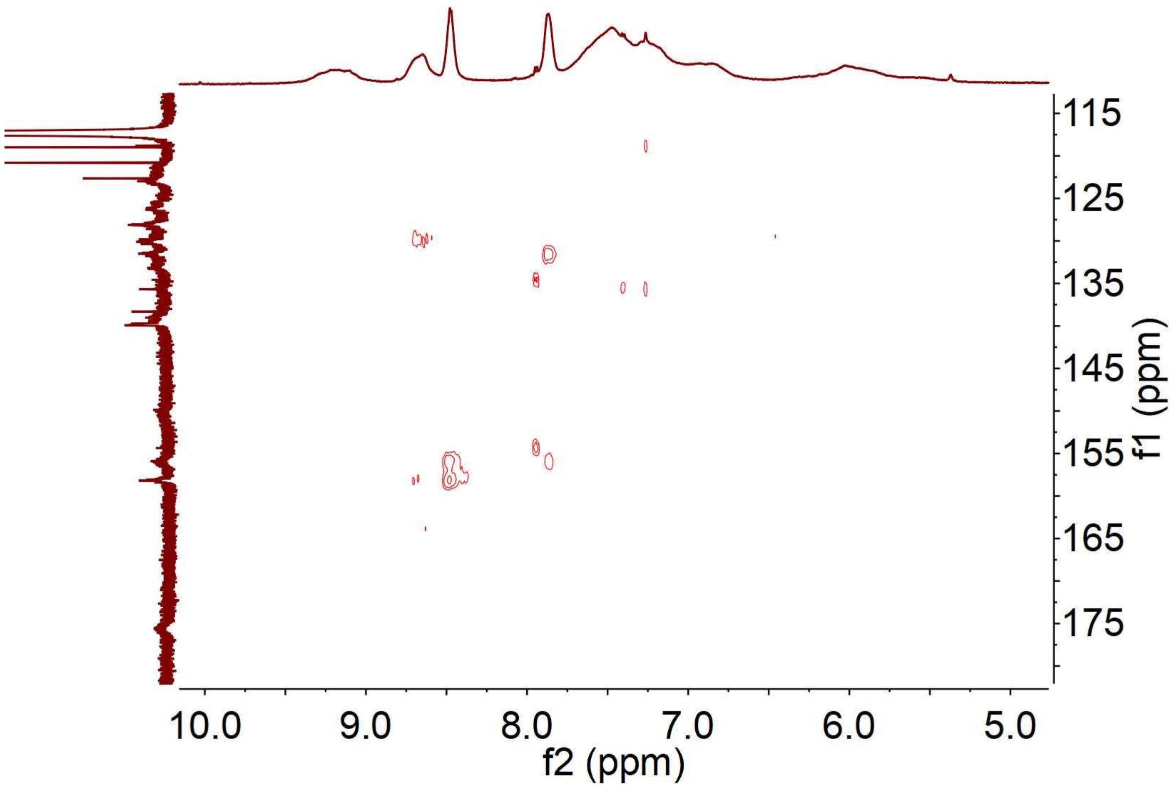

Figure S17. Partial ^{1}H-^{13}C HMBC NMR spectrum of **3-Fe^{II}** (500 MHz, CD_3CN, 298 K).

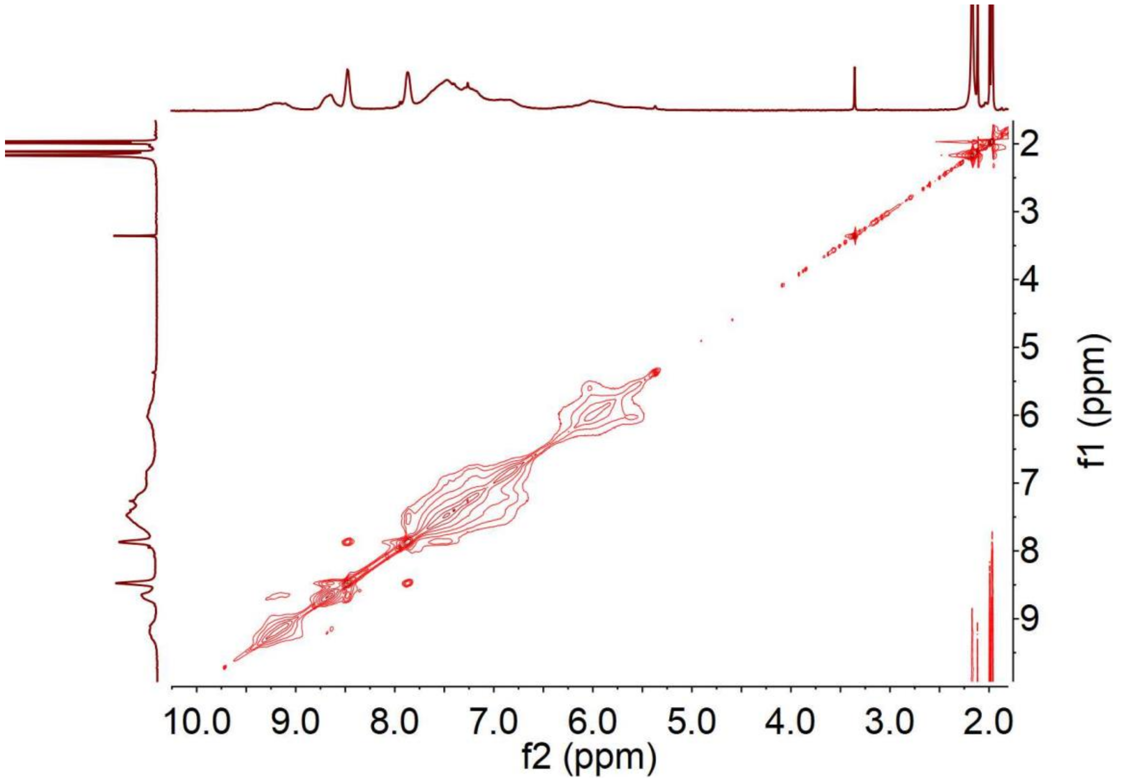

Figure S18. ^{1}H-^{1}H NOESY NMR spectrum of **3-Fe^{II}** (500 MHz, CD_3CN, 298 K).

S2.3.4 Characterization of **4-Fe^{II}**

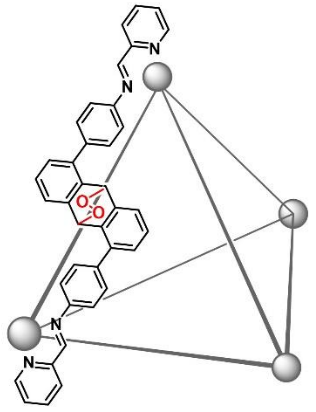

4-Fe^{II}

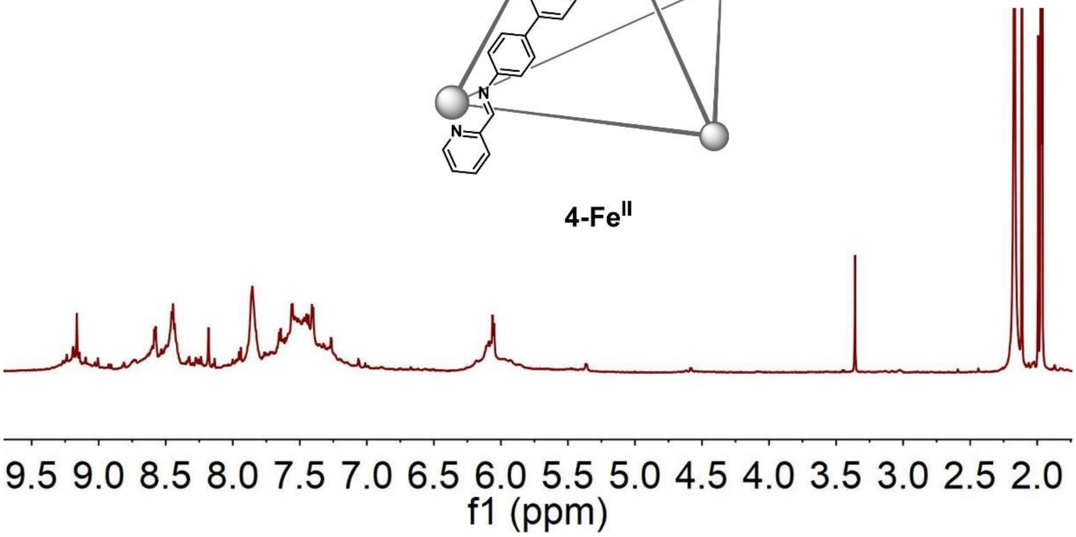

Figure S19. ^{1}H NMR spectrum of **4-Fe^{II}** (500 MHz, CD_3CN, 298 K).

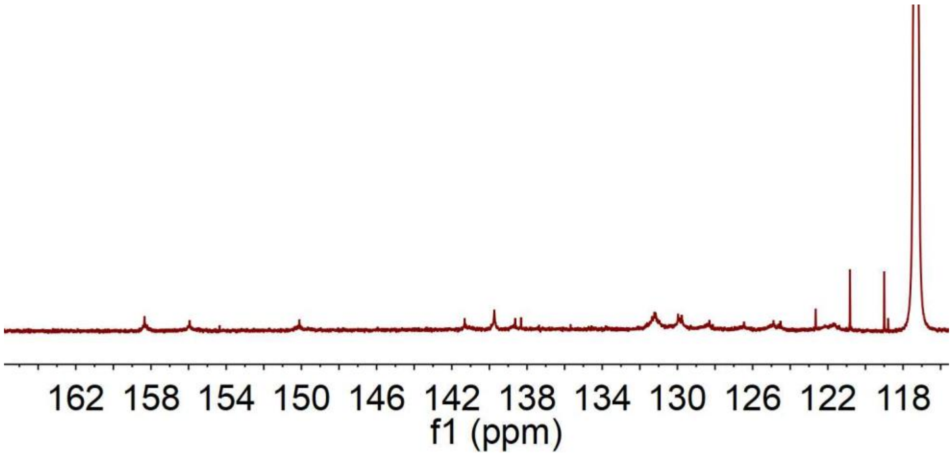

Figure S20. ^{13}C NMR spectrum of **4-Fe^{II}** (126 MHz, CD_3CN, 298 K).

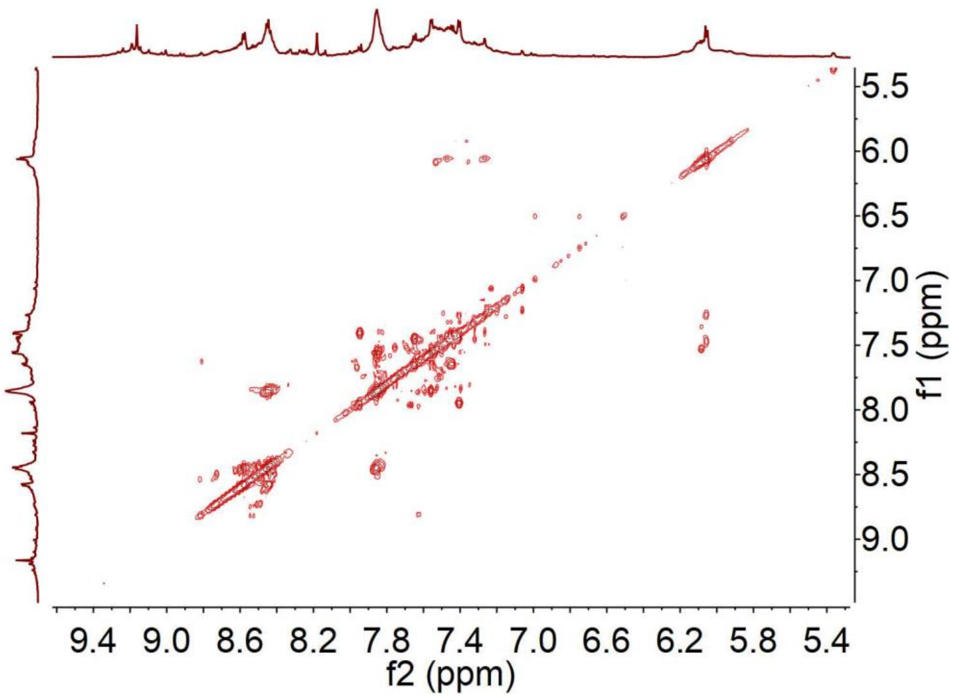

Figure S21. Partial ^{1}H-^{1}H COSY NMR spectrum of **4-Fe^{II}** (500 MHz, CD_3CN, 298 K).

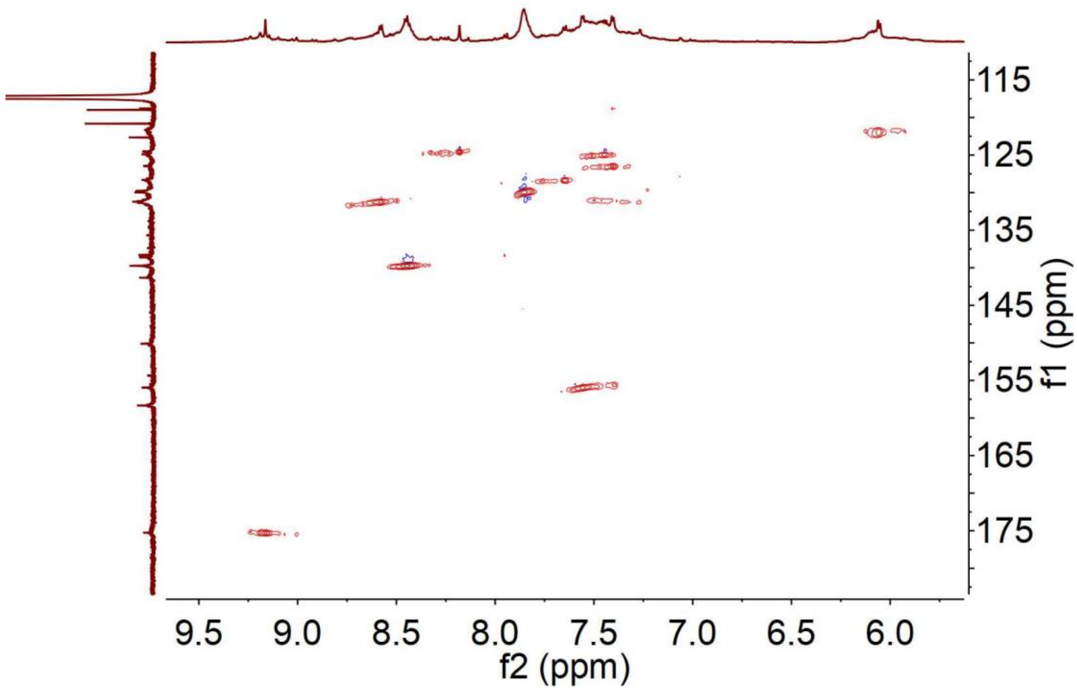

Figure S22. Partial ^{1}H-^{13}C HSQC NMR spectrum of **4-Fe^{II}** (500 MHz, CD_3CN, 298 K).

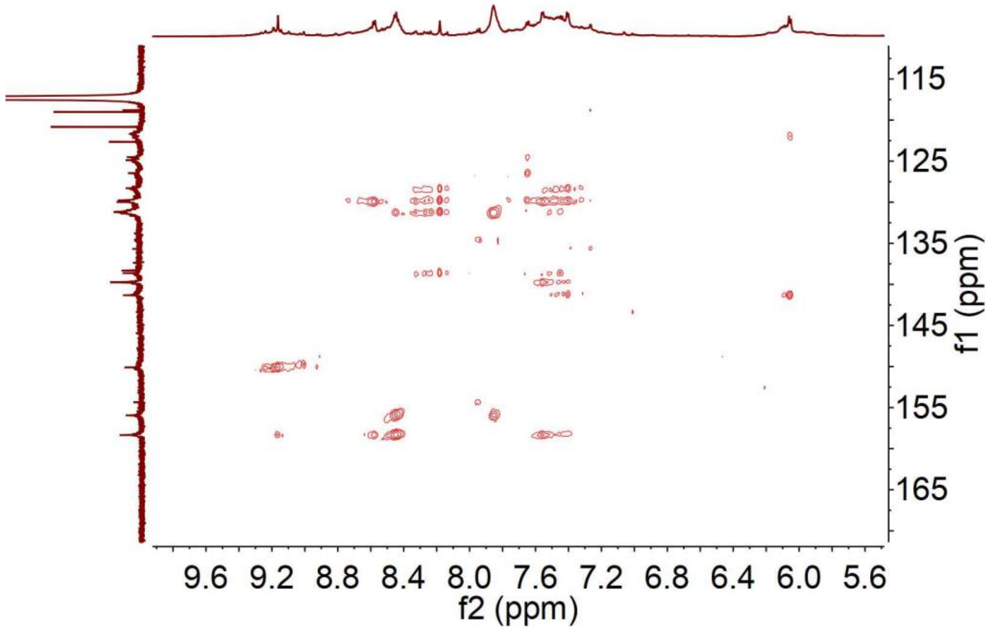

Figure S23. Partial ^{1}H-^{13}C HMBC NMR spectrum of **4-Fe^{II}** (500 MHz, CD_3CN, 298 K).

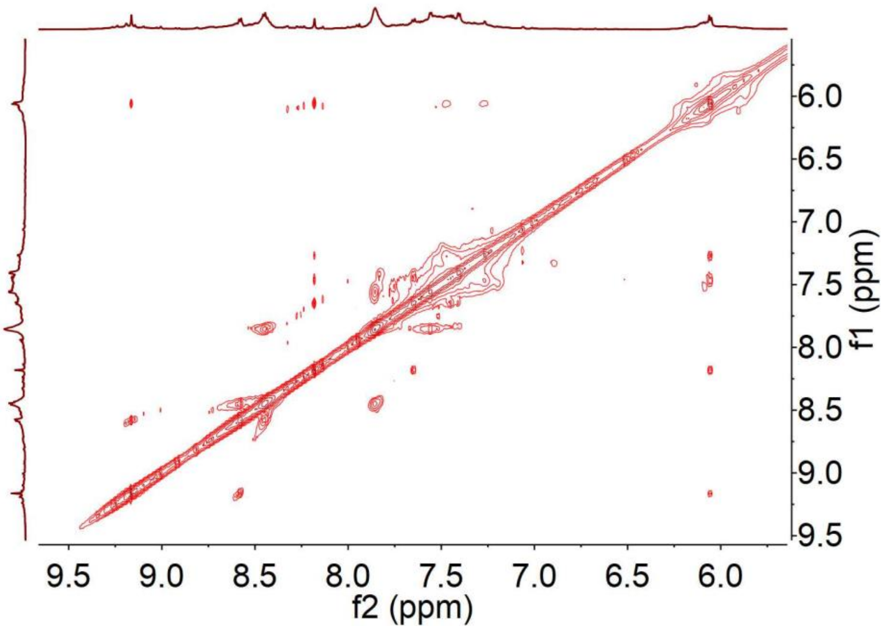

Figure S24. Partial ^{1}H-^{1}H NOESY NMR spectrum of **4-Fe^{II}** (500 MHz, CD_3CN, 298 K).

S2.3.5 ^{1}H NMR spectra of **1-Co^{II}** and **3-Co^{II}**

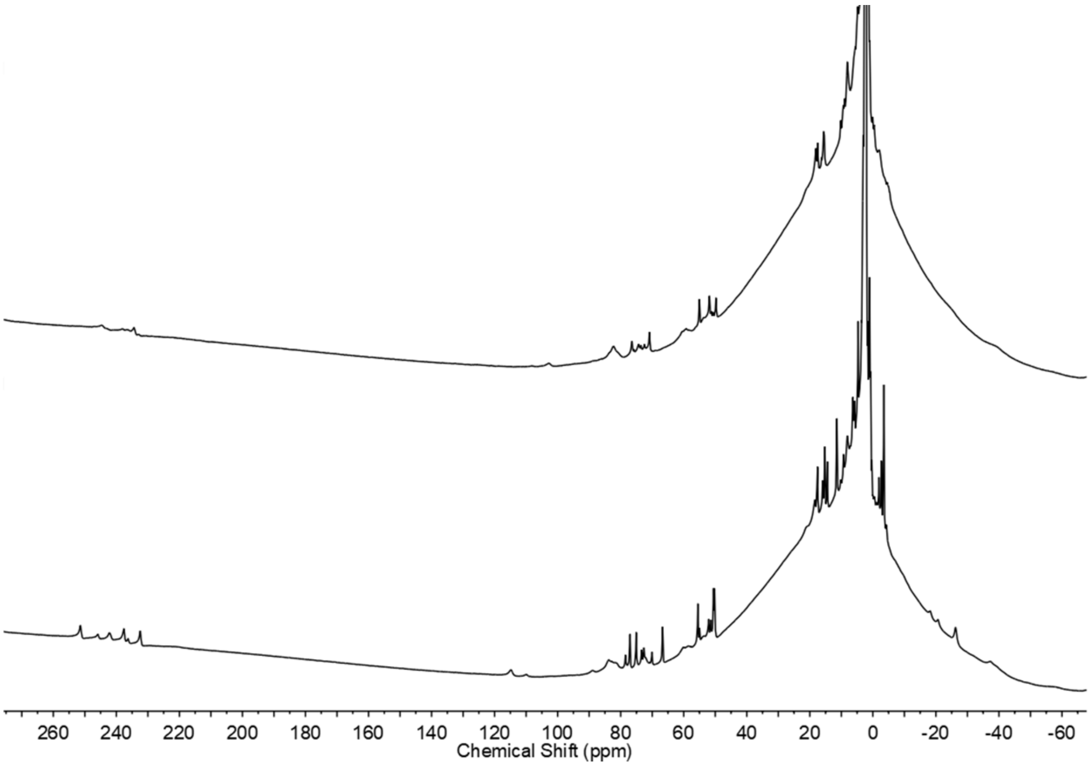

Figure S25. ^{1}H NMR (400 MHz, CD_3CN, 298 K) spectra of cage **1-Co^{II}** before (bottom) and after (top) the reaction with singlet oxygen to produce **3-Co^{II}**.

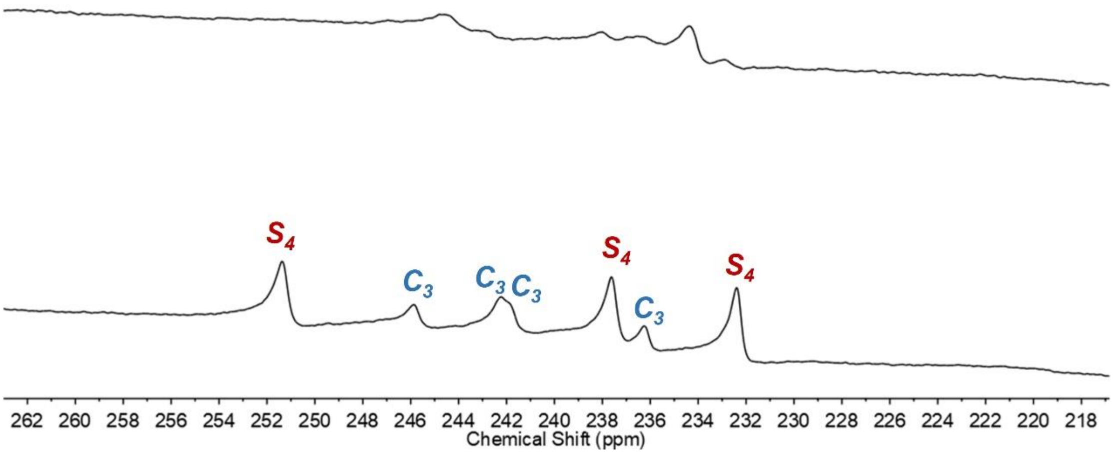

Figure S26. Imine region of the ^{1}H NMR (400 MHz, CD_3CN, 298 K) spectra of cage **1-Co^{II}** before (bottom) and after (top) the reaction with singlet oxygen to produce **3-Co^{II}**. Upon photooxygenation in solution, addition of O_2 to the cage on either face of each anthracene panel would result in the generation of many different diastereomers, consistent with the complex observed spectrum for **3-Co^{II}**.

S2.3.6 ^{1}H NMR spectra of **2-Co^{II}** and **4-Co^{II}**

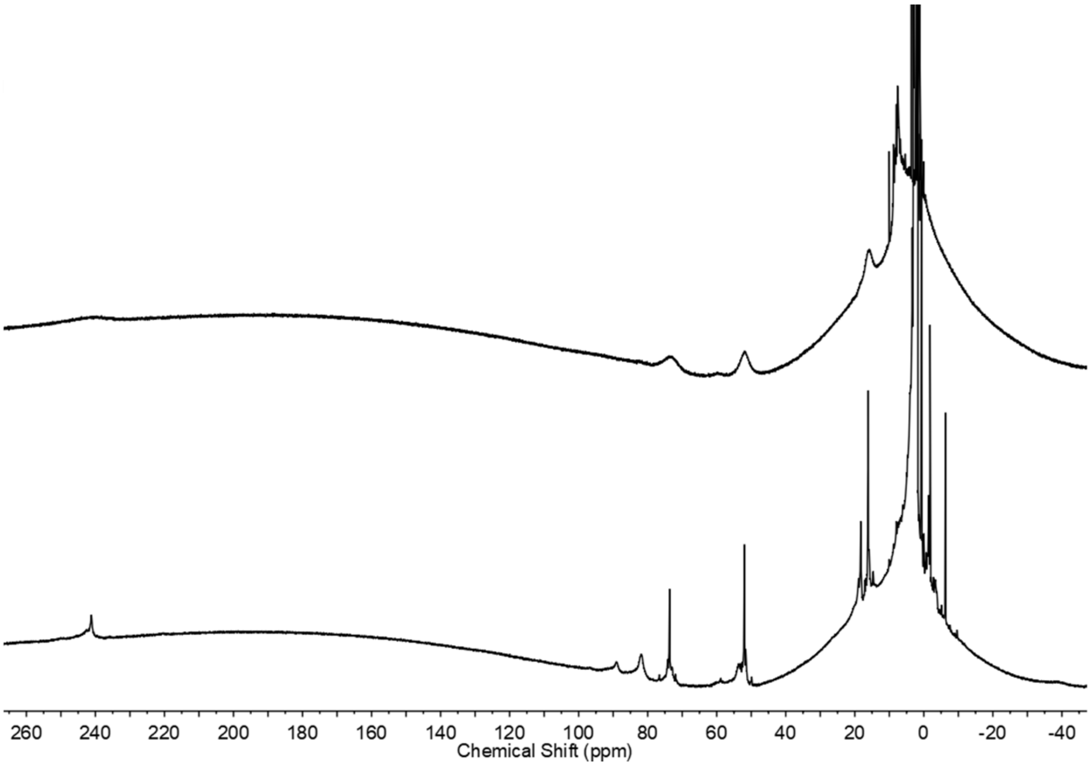

Figure S27. ^{1}H NMR (400 MHz, CD_3CN, 298 K) spectra of cage **2-Co^{II}** before (top) and after (bottom) the reaction with singlet oxygen to produce **4-Co^{II}**.

S2.3.7 ^{1}H NMR spectra of **1-Fe^{II}** and **3-Fe^{II}**

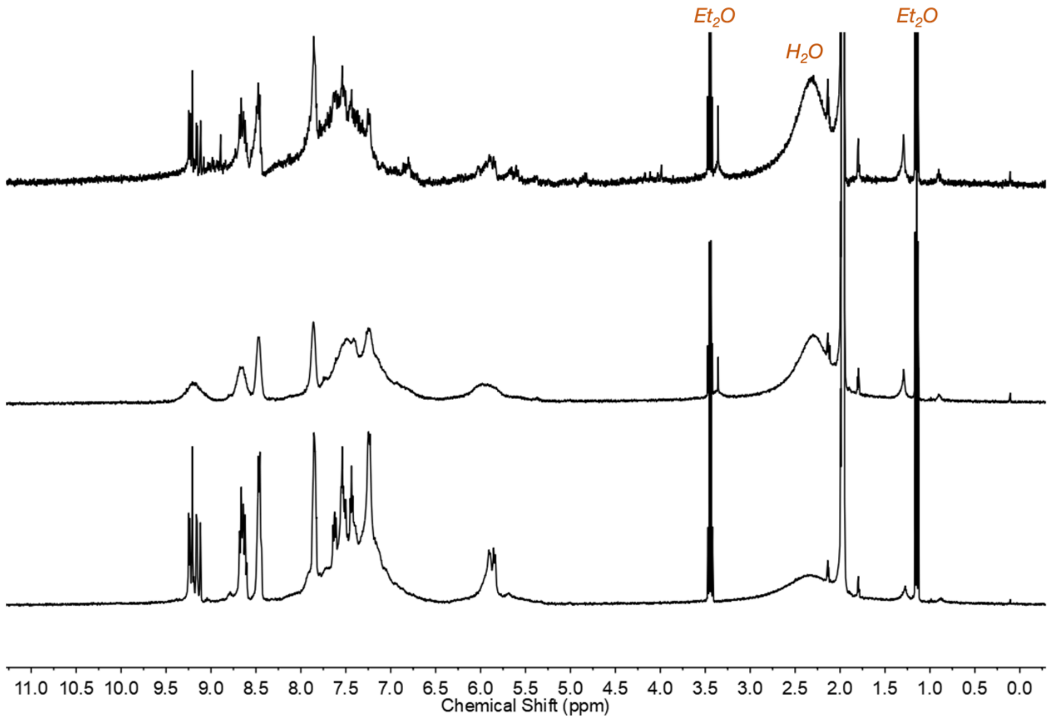

Figure S28. ^{1}H NMR (400 MHz, CD_3CN, 298 K) spectra of cage **1-Fe^{II}** before (bottom), after the reaction with singlet oxygen to produce **3-Fe^{II}** (middle) and after subjecting **3-Fe^{II}** to heating at 50 °C for three days (top).

S2.3.8 ^{1}H NMR spectra of **2-Fe^{II}** and **4-Fe^{II}**

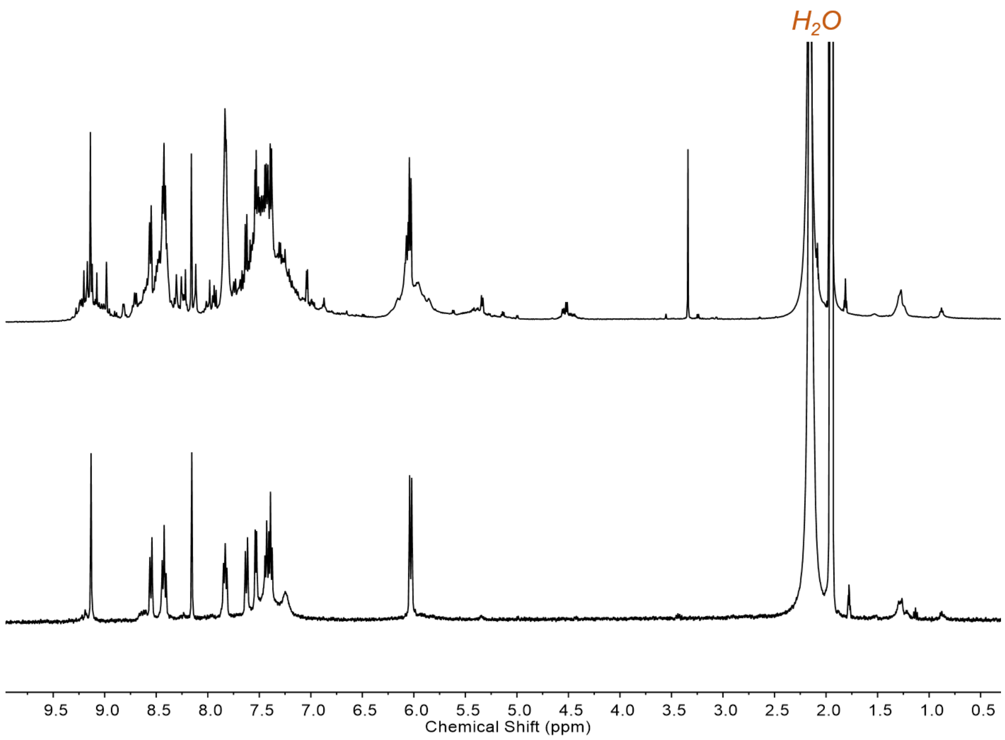

Figure S29. ^{1}H NMR (500 MHz, CD_3CN, 298 K) spectra of cage **2-Fe^{II}** before (bottom) and after the reaction with singlet oxygen to produce **4-Fe^{II}** (top).

S2.3.9 DOSY NMR spectra of **1-Fe**^{II}, **2-Fe**^{II}, **3-Fe**^{II} and **4-Fe**^{II}

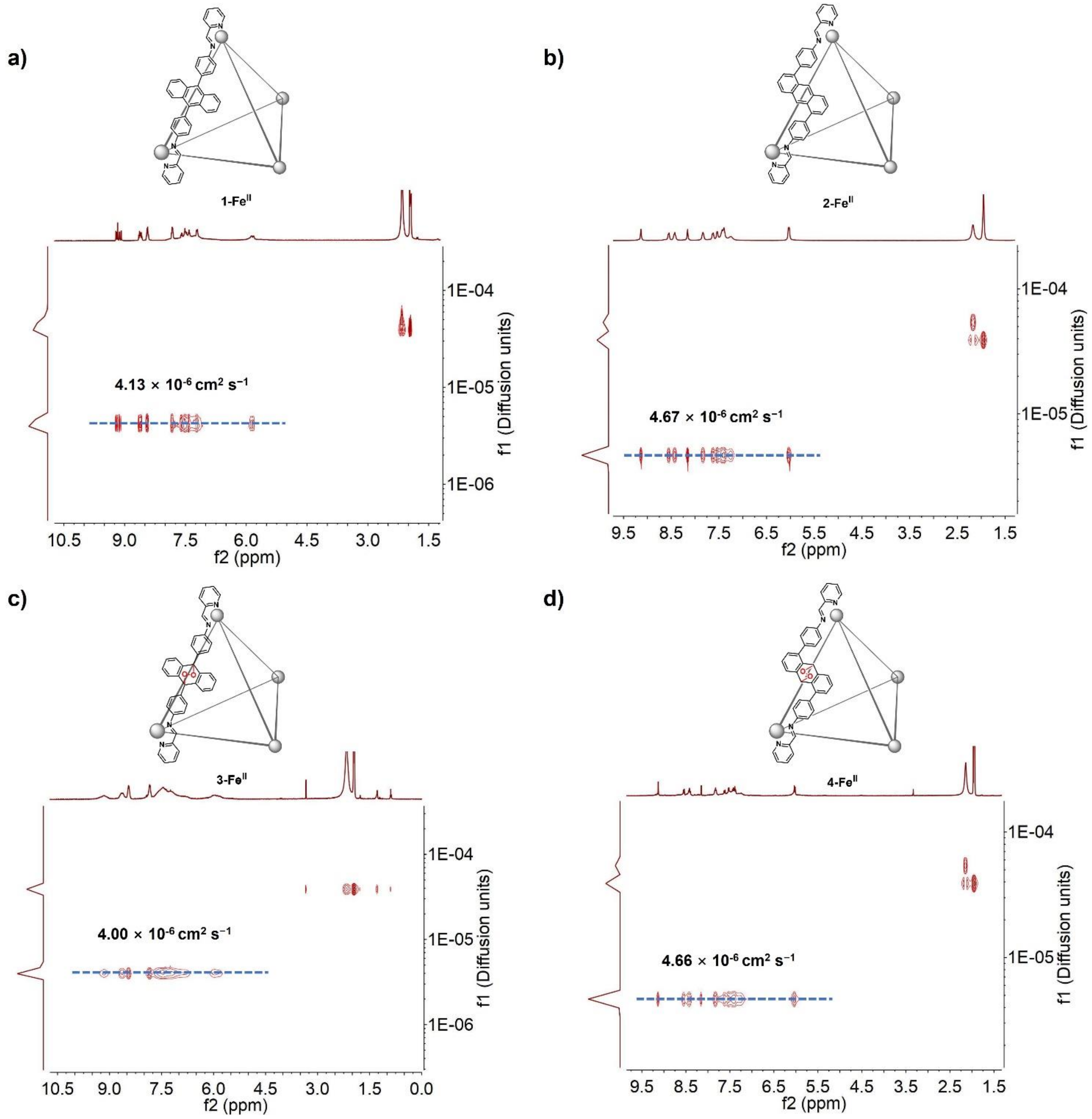

Figure S31. ^{1}H DOSY NMR spectra of **1-Fe**^{II}, **2-Fe**^{II}, **3-Fe**^{II} and **4-Fe**^{II} (400 MHz, CD_3CN, 298 K). a) **1-Fe**^{II} (0.5 mM). The diffusion coefficient for **1-Fe**^{II} was determined to be 4.13 × 10^{-6} cm^2 s^{-1}, corresponding to a hydrodynamic radius of 15.8 Å; b) **2-Fe**^{II} (0.5 mM). The diffusion coefficient for **2-Fe**^{II} was determined to be 4.67 × 10^{-6} cm^2 s^{-1}, corresponding to a hydrodynamic radius of 14.0 Å; c) **3-Fe**^{II} (0.5 mM). The diffusion coefficient for **3-Fe**^{II} was determined to be 4.00 × 10^{-6} cm^2 s^{-1}, corresponding to a hydrodynamic radius of 16.3 Å; d) **4-Fe**^{II} (0.5 mM). The diffusion coefficient for **4-Fe**^{II} was determined to be 4.66 × 10^{-6} cm^2 s^{-1}, corresponding to a hydrodynamic radius of 14.0 Å.

S2.4. Low-resolution ESI-mass spectrometry data

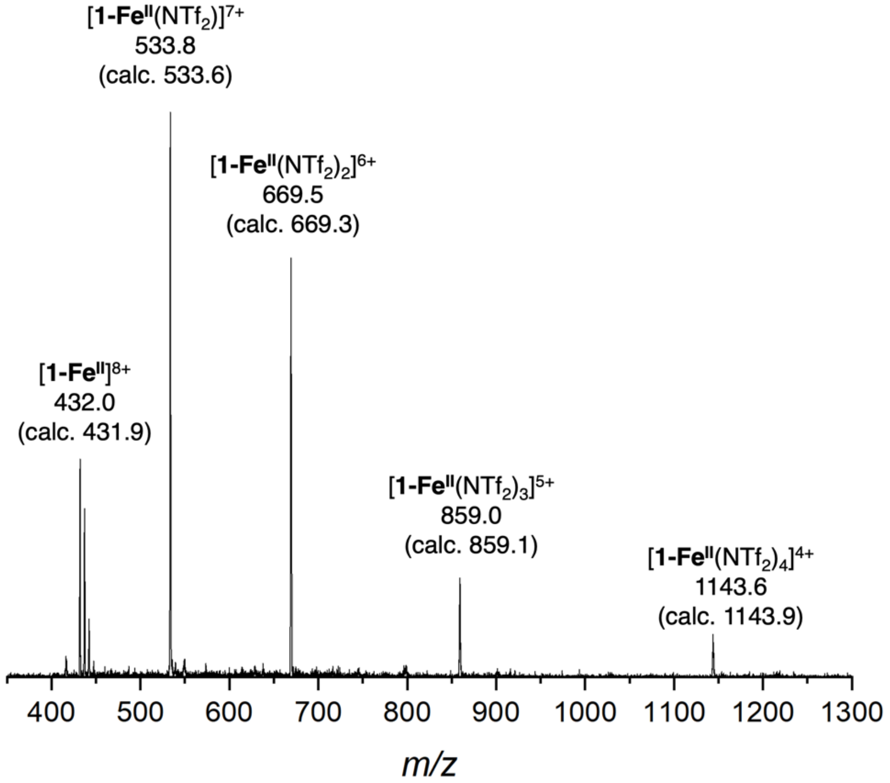

Figure S31. Low-resolution ESI-mass spectrum of cage **1-Fe^{II}** ·8NTf$_2$.

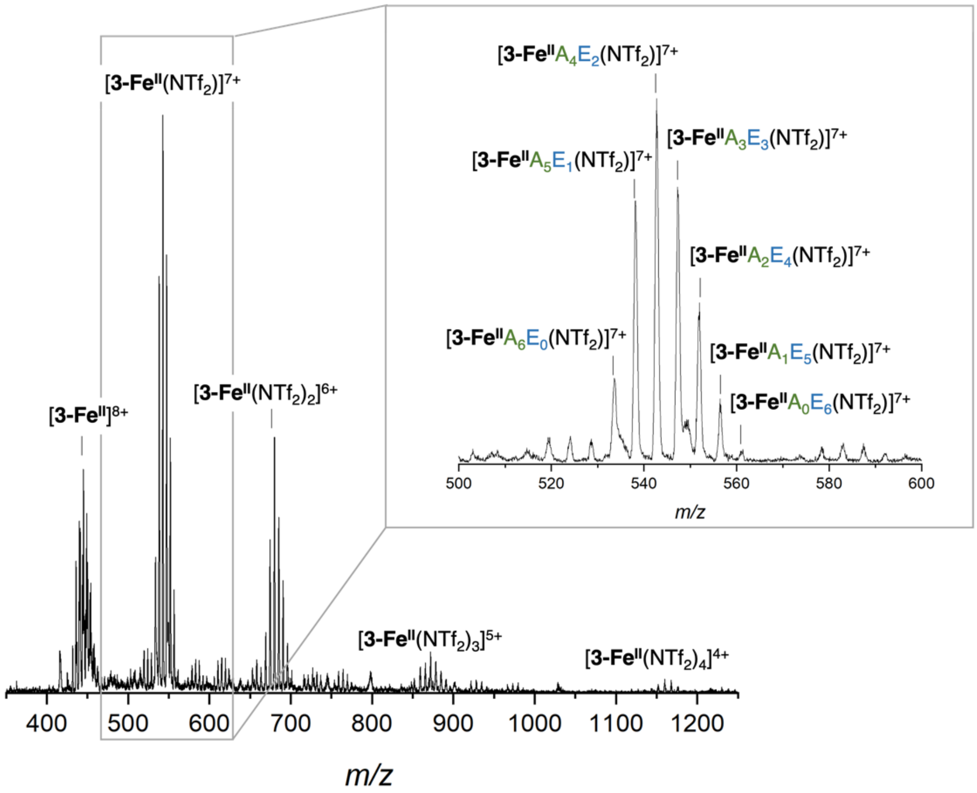

Figure S32. Low-resolution ESI-mass spectrum of cages **3-Fe^{II}** ·8NTf$_2$.

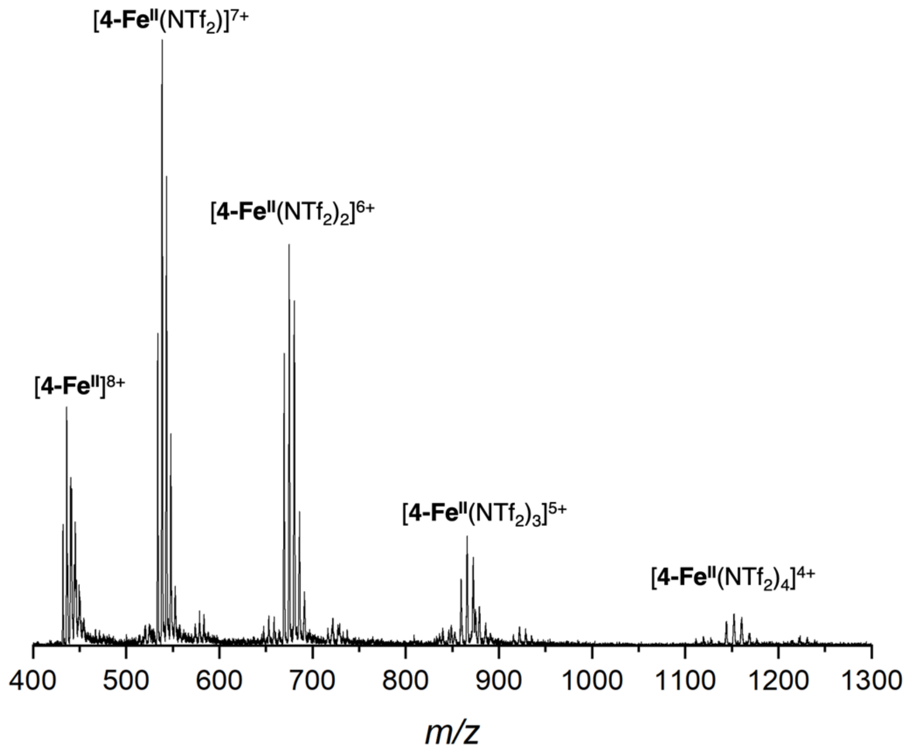

Figure S33. Low-resolution ESI-mass spectrum of cages **4-Fe^{II}** ·8NTf$_2$.

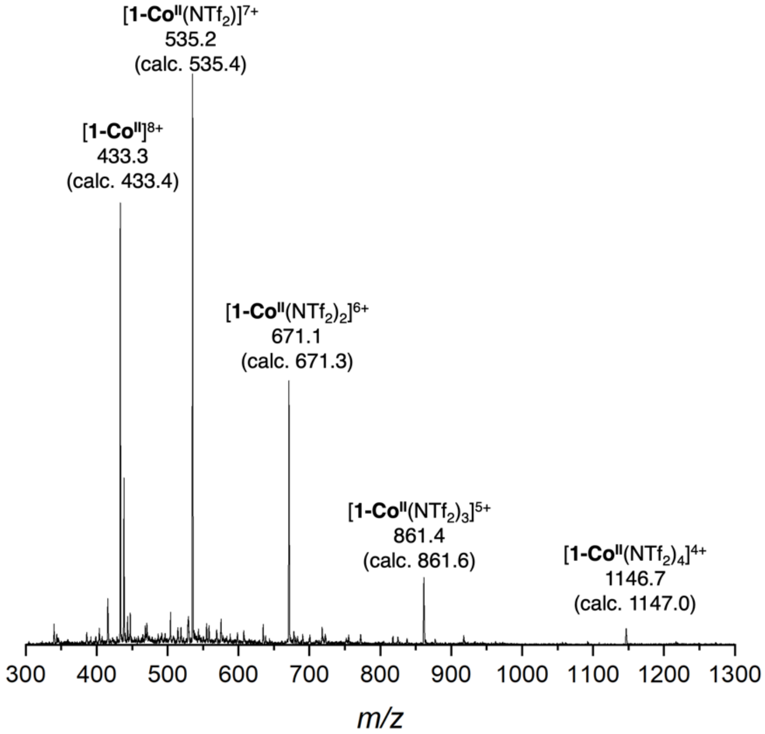

Figure S34. Low-resolution ESI-mass spectrum of cage **1-Co^{II}** ·8NTf$_2$.

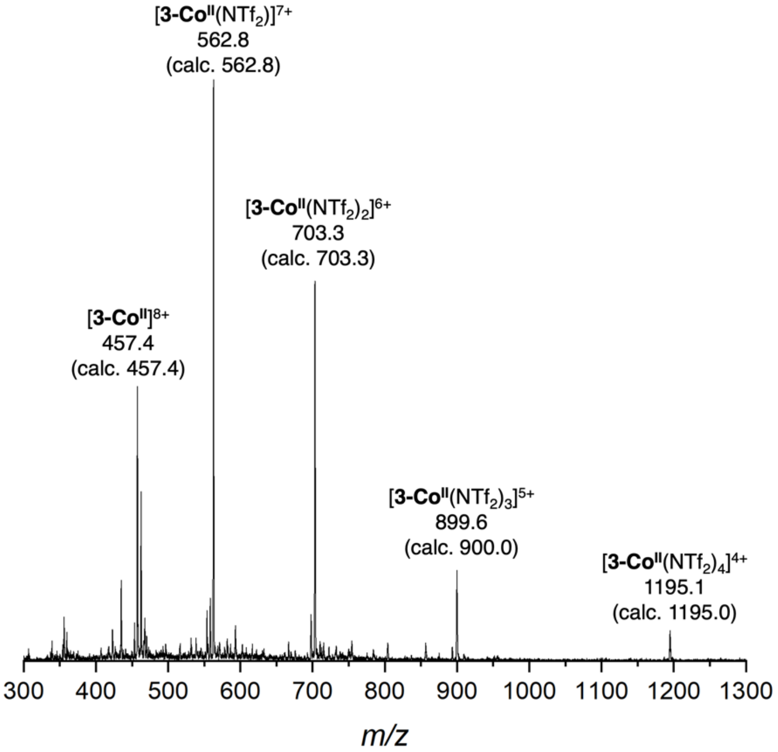

Figure S35. Low-resolution ESI-mass spectrum of cage **3-Co^{II}** ·8NTf$_2$.

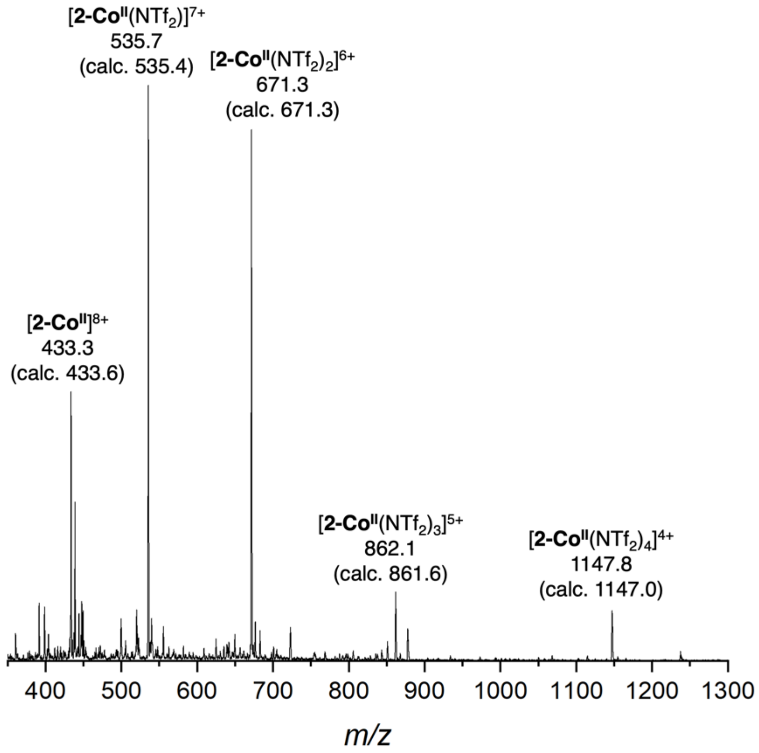

Figure S36. Low-resolution ESI-mass spectrum of cage **2-Co^{II}** ·8NTf$_2$.

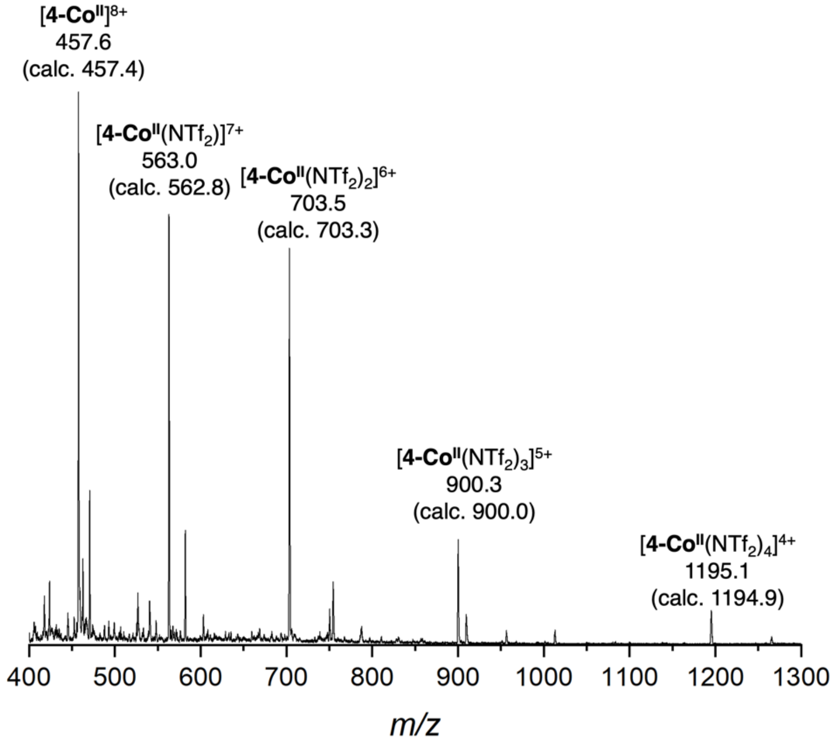

Figure S37. Low-resolution ESI-mass spectrum of cage **4-Co^{II}** ·8NTf$_2$.

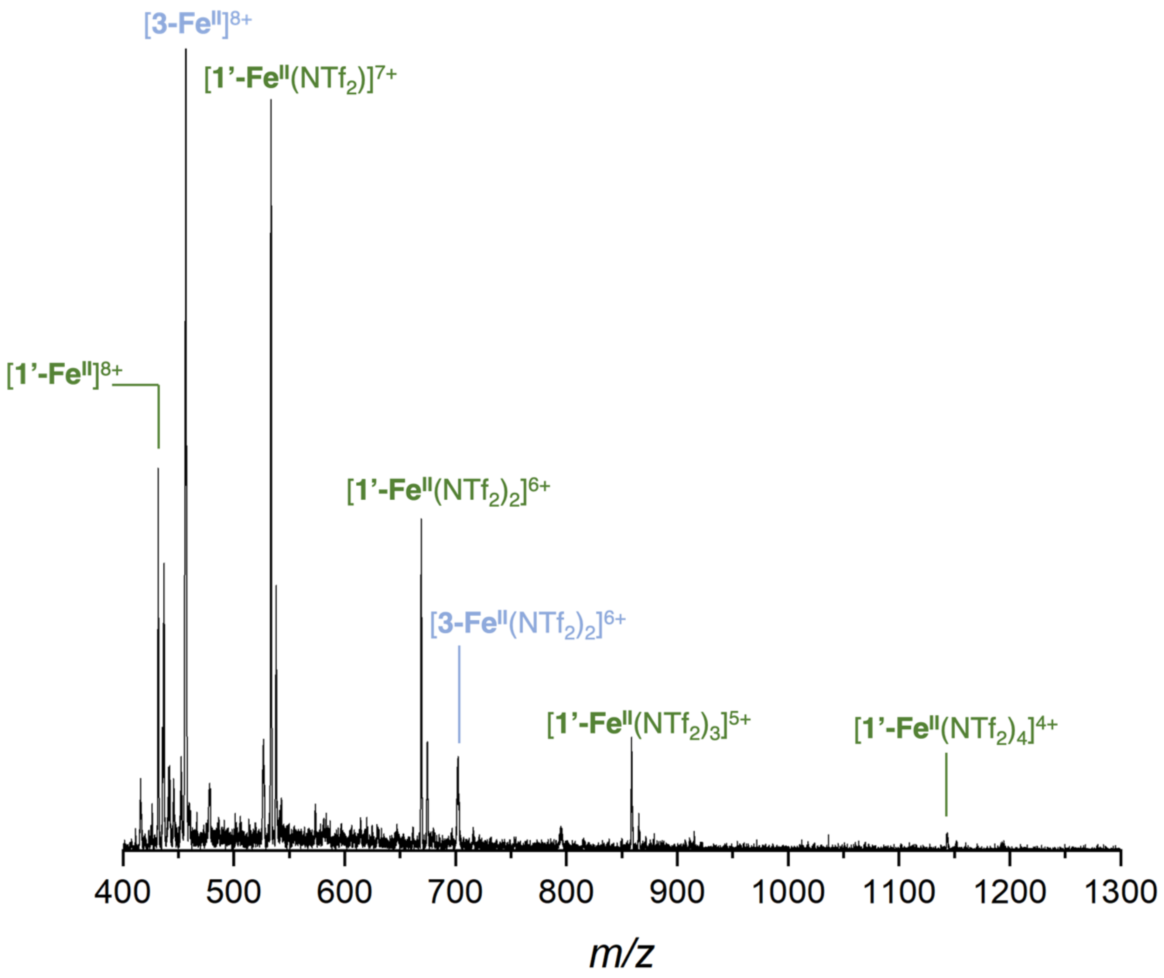

Figure S38. Low-resolution ESI-mass spectrum obtained after heating an acetonitrile solution of cage **3-Fe^{II}** at 50°C for 3 days. The solution contains a mixture of the modified cage **3-Fe^{II}** ·8NTf$_2$ and **1'-Fe^{II}** ·8NTf$_2$ obtained during retro-cycloaddition. Certain fragments are labeled as cages **1'-Fe^{II}** to emphasize the fact that these are cycloreversion products.

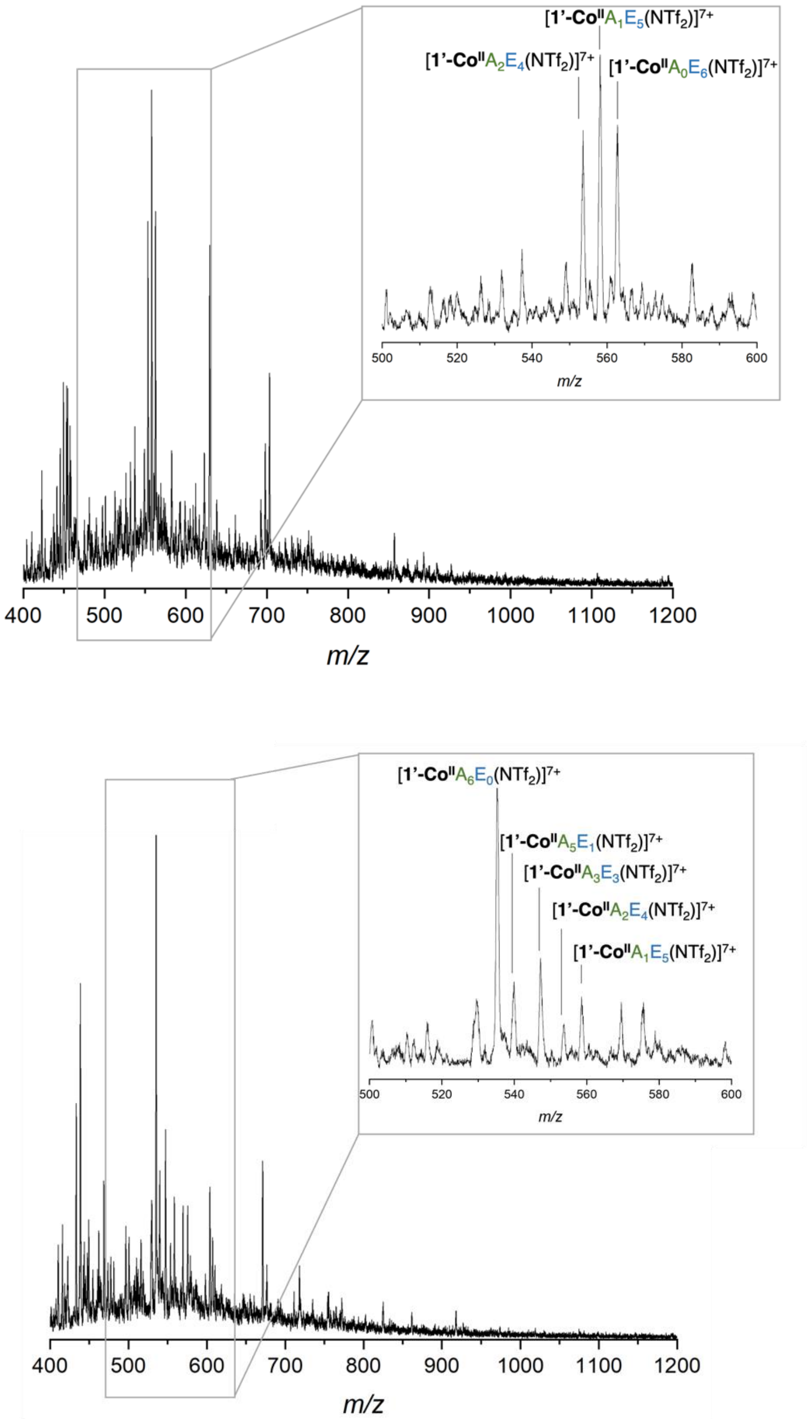

Figure S39. Low-resolution ESI-mass spectra obtained after heating solid samples of cages **3-Co^{II}** under a dynamic vacuum at 80°C (top) and 160°C (bottom) for 16 hours. Fragments are labeled as cages **1'-Co^{II}** to emphasize the fact that these are cycloreversion products.

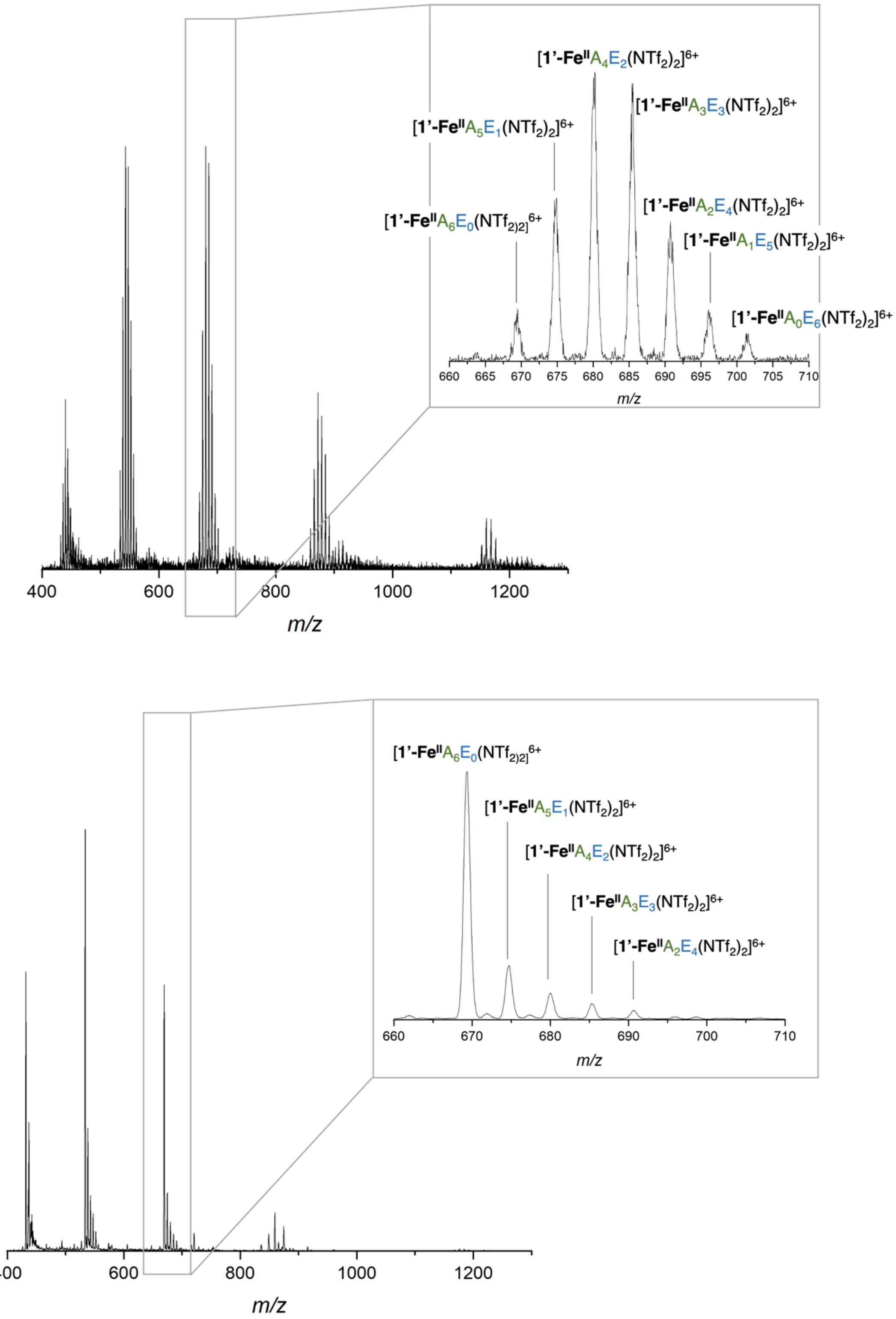

Figure S40. Low-resolution ESI-mass spectra obtained after heating solid samples of cages **3-Fe^{II}** before heating (top) and after being under a dynamic vacuum at 160°C for 16 hours (bottom). Fragments are labeled as cages **1'-Fe^{II}** to emphasize the fact that these are cycloreversion products.

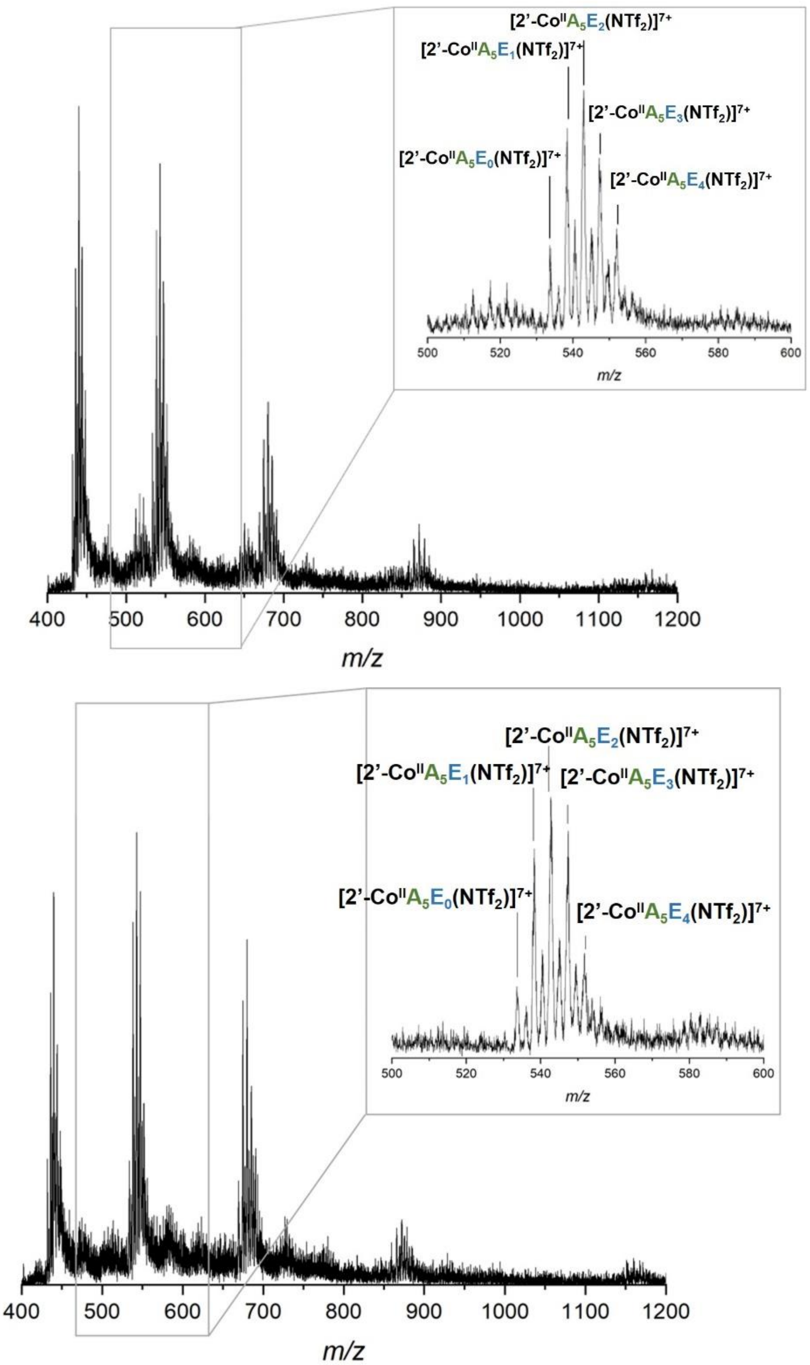

Figure S41. Low-resolution ESI-mass spectra obtained after heating solid samples of cages **4-Co^{II}** under a dynamic vacuum at 80°C (top) and 160°C (bottom) for 16 hours. Fragments are labeled as cages **2’-Co^{II}** to emphasize that these are cycloreversion products.

S2.5. High-resolution mass spectrometry data

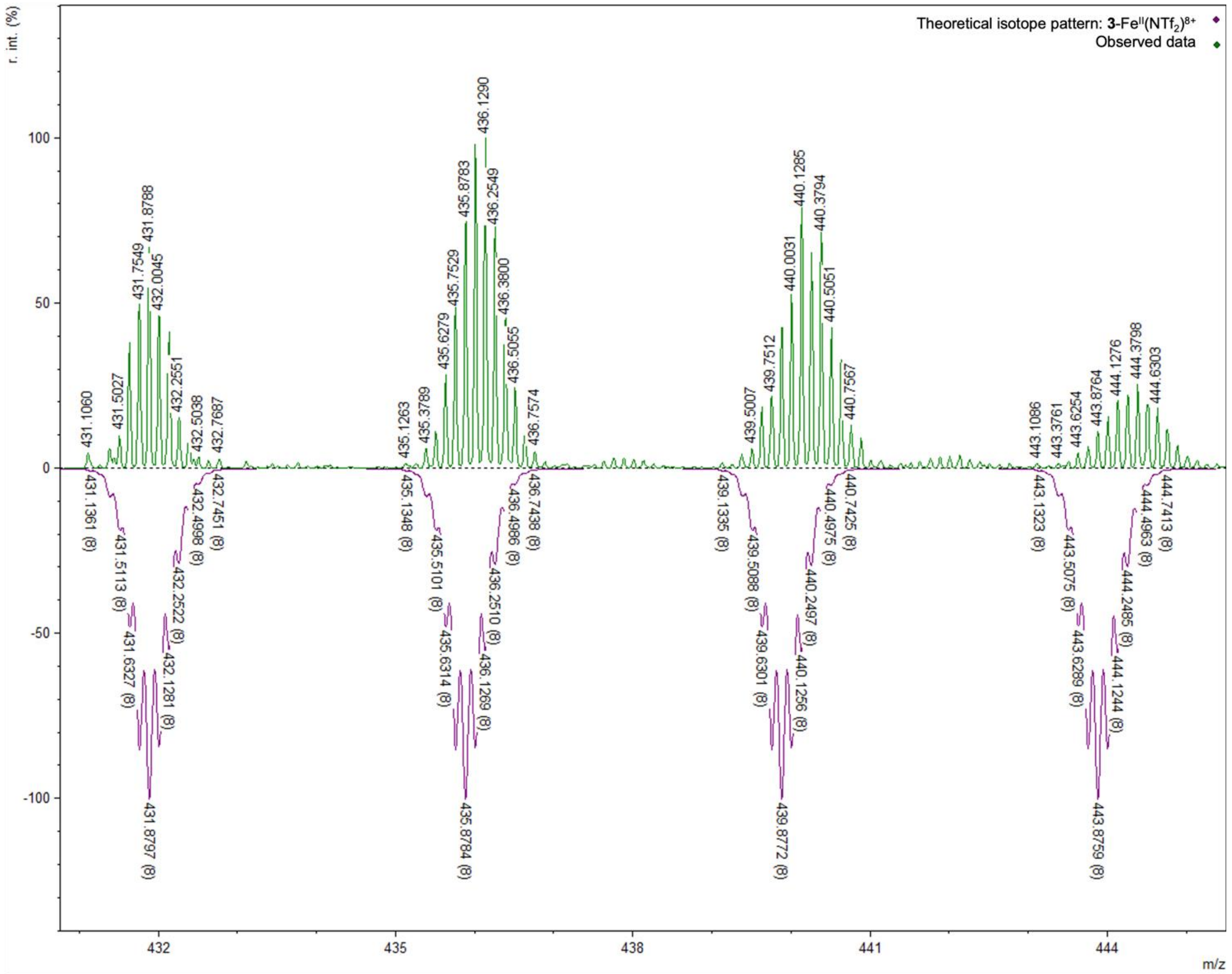

Figure S42. High-resolution mass spectrometry analysis of **3-Fe^{II}** showing the +8 peak. The fragmentation pattern observed for the +8 peak is attributed to homolytic fragmentation of the $[Fe_4L_6]^{8+}$ assembly under the MS conditions. The ionisation conditions of the instrument used to record the high-resolution ESI-MS are harsher than those used to record the low-resolution ESI-MS in Figure S33, as evidenced by much higher levels of fragmentation overall and the observation of strong peaks for low charged +1 and +2 fragments. This matches observations reported previously.[5]

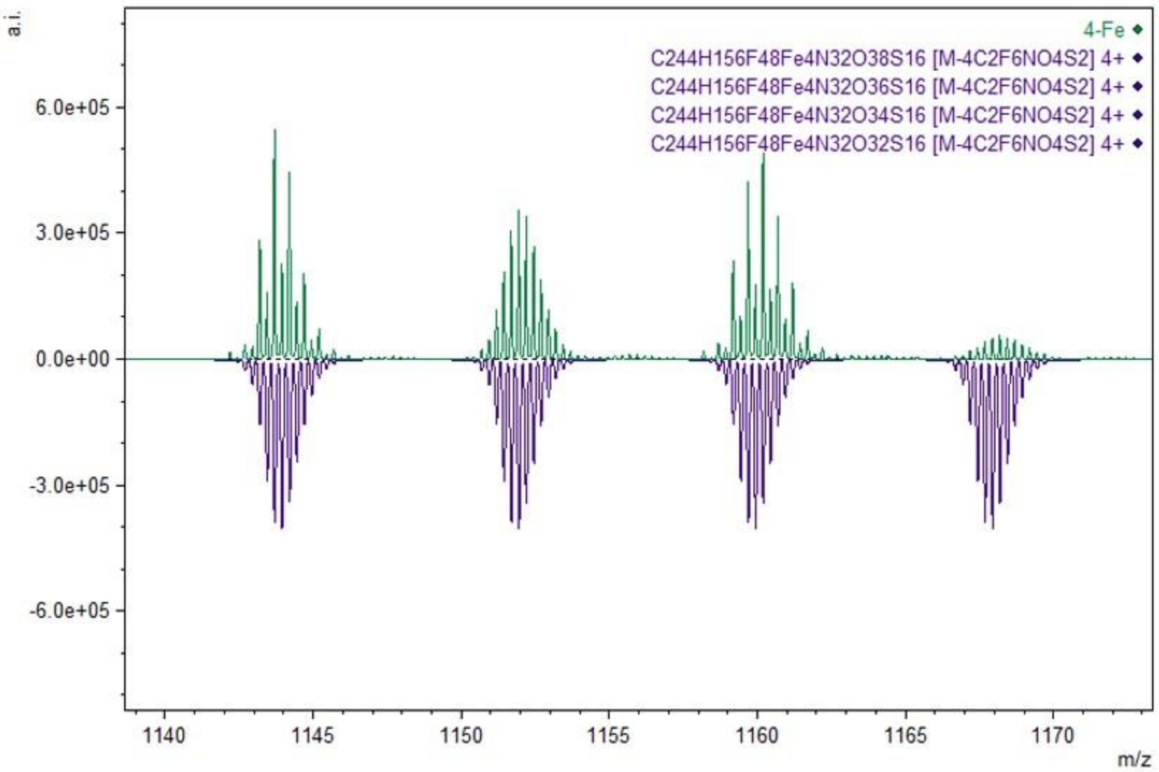

Figure S43. High-resolution mass spectrometry analysis of **4-Fe^{II}** sample, showing the observed (blue) and theoretical (purple) isotope patterns for +4 charged ions. The left signal corresponds to the parent **2-Fe^{II}**, while the right three signals correspond to ions incorporating progressively more O_2 moities per assembly, with 1 equiv, 2 equiv, and 3 equiv respectively.

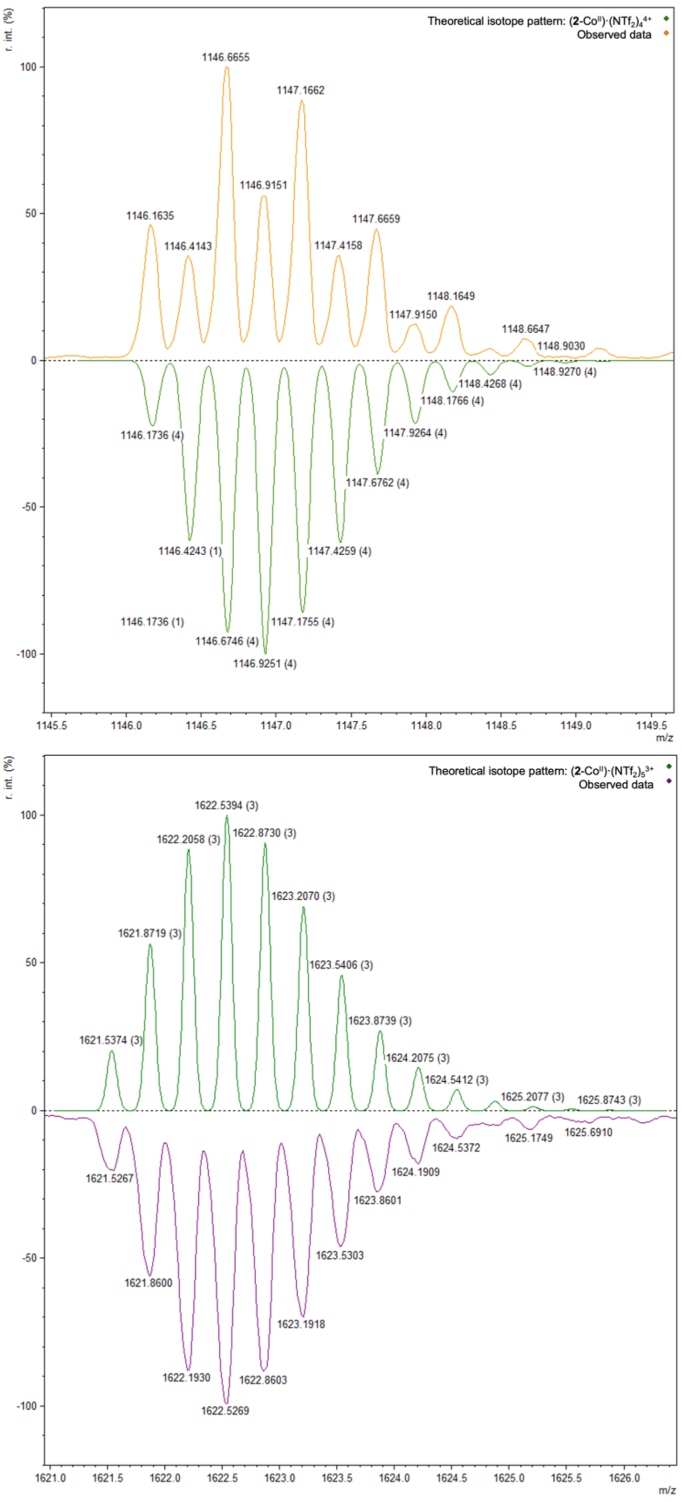

Figure S44. High-resolution mass spectrometry analysis of **2-Co^{II}** showing the +4 (top) and +3 (bottom) peaks.

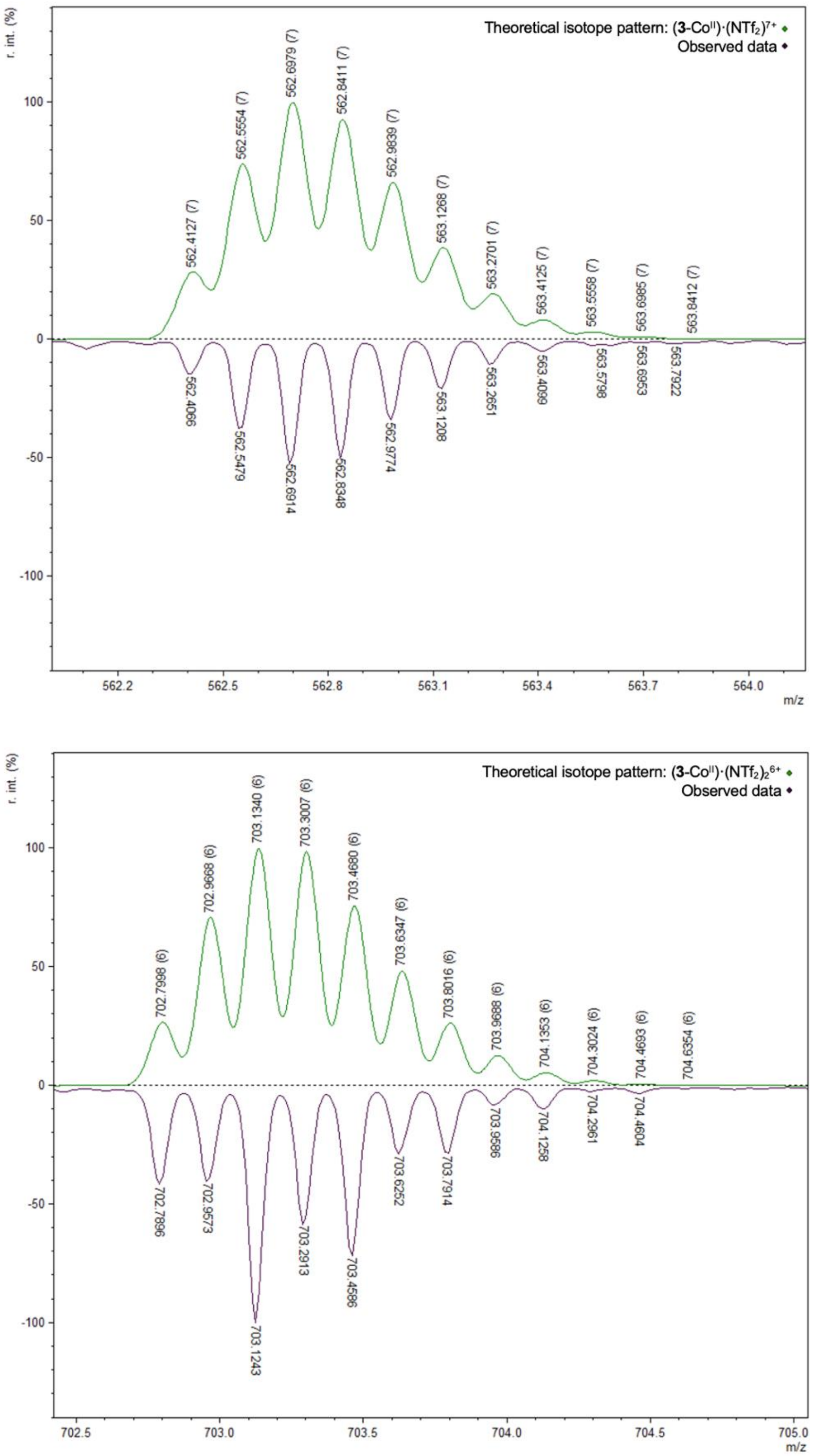

Figure S45. High-resolution mass spectrometry analysis of **3-Co^{II}** showing the +7 (top) and +6 (bottom) peaks. The fragmentation pattern observed for the +6 peak is attributed to homolytic fragmentation of the $[Co_4L_6]^{8+}$ assembly under the MS conditions. The ionisation conditions of the instrument used to record the high-resolution ESI-MS are harsher than those used to record the low-resolution ESI-MS in Figure S10, as evidenced by much higher levels of fragmentation overall and the observation of strong peaks for low charged +1 and +2 fragments. This matches observations reported previously.[5]

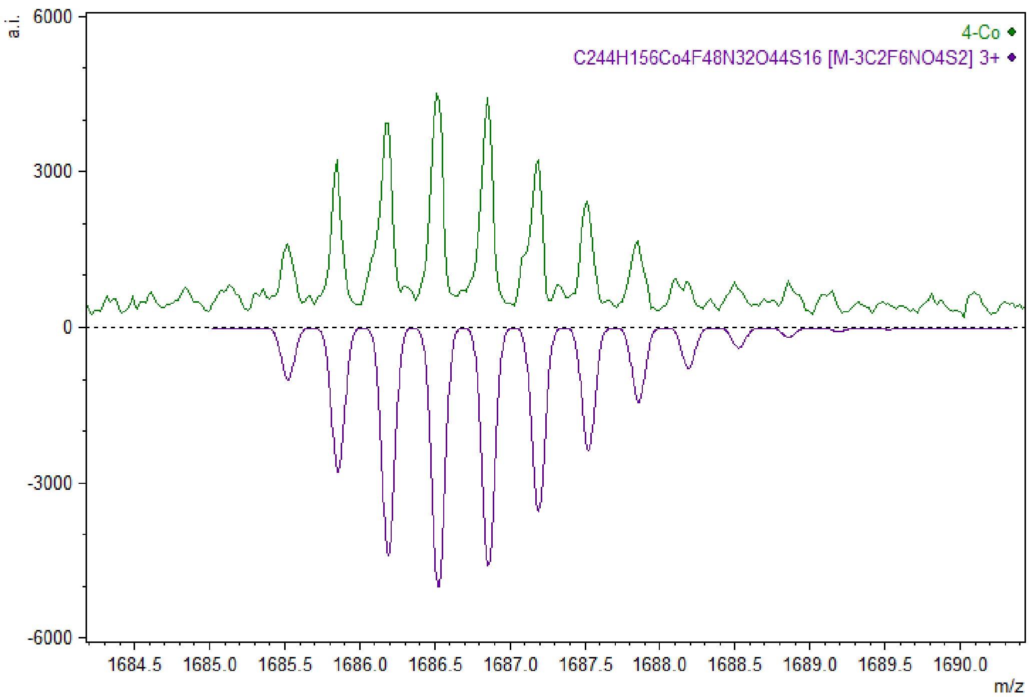

Figure S46. High-resolution mass spectrometry analysis of **4-Co**^{II} showing the observed (blue) and theoretical (purple) isotope patterns for the +3 ion.

S2.6. UV-visible spectroscopy data

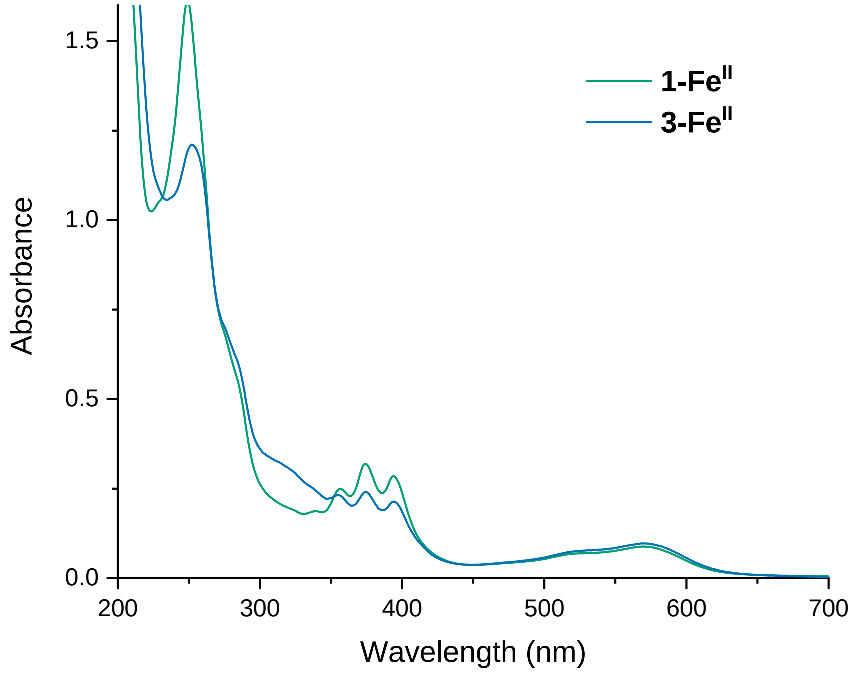

Figure S47. UV-Vis spectra of the precursor cage **1-Fe^{II}** (green line) and the resulting mixture of cages denoted as **3-Fe^{II}** (blue line) after the hetero-Diels-Alder reaction with $^1O_2$.

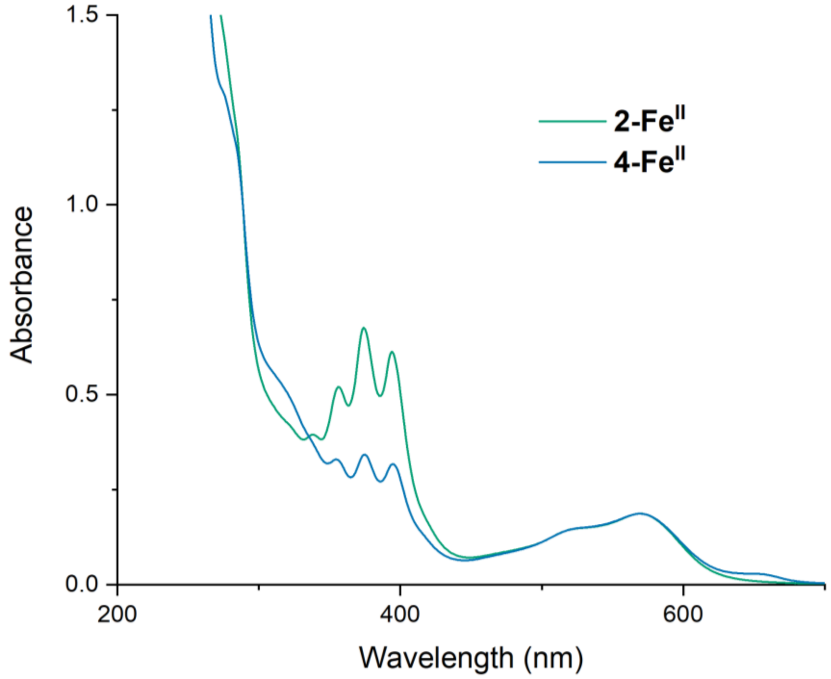

Figure S48. UV-Vis spectra of the precursor cage **2-Fe^{II}** (green line) and the resulting mixture of cages denoted as **4-Fe^{II}** (blue line) after the hetero-Diels-Alder reaction with $^1O_2$.

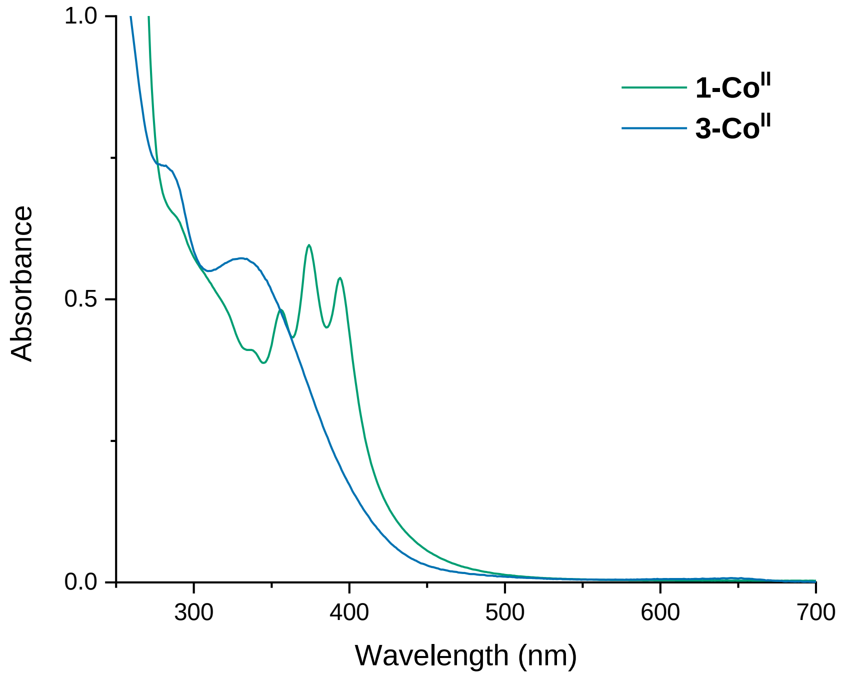

Figure S49. UV-Vis spectra of the precursor cage **1-Co^{II}** (green line) and the cage **3-Co^{II}** (blue line) after the hetero-Diels-Alder reaction with $^1O_2$.

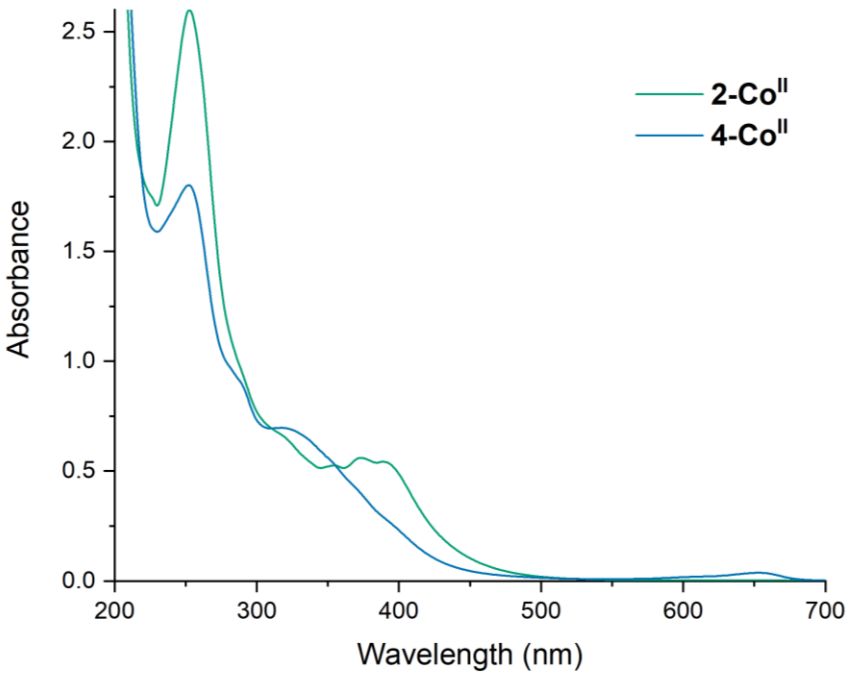

Figure S50. UV-Vis spectra of the precursor cage **2-Co^{II}** (green line) and the cage **4-Co^{II}** (blue line) after the hetero-Diels-Alder reaction with $^1O_2$.

S3. Host-guest chemistry experiments

S3.1. Summary of ITC results

Table S1. Summary of isothermal titration calorimetry data for titration of pyrene, phenanthrene, and *β*-endosulfan with each of the hosts **1-Co^{II}**, **2-Co^{II}**, **3-Co^{II}**, and **4-Co^{II}**.

Guest molecule	Host	K_a (M^{-1})	ΔH (cal/mol)	ΔS (cal/mol/deg)
pyrene	**1-Co^{II}**	K_{a1} = 740 ± 58;<br>K_{a2} = 5.08×10^3 ± 3.20×10^2	ΔH_1 = -483.5 ± 58.5;<br>ΔH_2 = -2.57×10^3 ± 72	ΔS_1 = 11.5<br>ΔS_2 = 8.35
	**2-Co^{II}**	K_{a1} = 630 ± 72;<br>K_{a2} = 250 ± 23	ΔH_1 = -198.6 ± 92.0;<br>ΔH_2 = -5.38×10^3 ± 296	ΔS_1 = 12.1<br>ΔS_2 = -7.07
	**3-Co^{II}**	6.4 ± 2.5	-1.40×10^3 ± 4.96×10^2	-0.996
	**4-Co^{II}**	<1.0		
phenanthrene	**1-Co^{II}**	641 ± 76.4	-601.9 ± 53.97	10.8
	**2-Co^{II}**	15 ± 0.48	-1.06 ×10^4 ± 281.1	-30.3
	**3-Co^{II}**	<1.0	-3.01×10^3 ± 1.03×10^8	-1.02 × 10^3
	**4-Co^{II}**	<1.0		
β-endosulfan	**1-Co^{II}**	(8.4 ± 0.82)×10^3	-5.96×10^3 ± 115.3	2.04
	**2-Co^{II}**	(7.6 ± 1.4)×10^3	-3.41×10^3 ± 212.5	6.34
	**3-Co^{II}**	21 ± 11	-683.2 ± 325.1	3.75
	**4-Co^{II}**	1.06×10^3 ± 3.27×10^2	-2.02×10^6 ± 5.12×10^9	-6.75 × 10^3

S3.2. Titration plots for host 1-Co^{II}

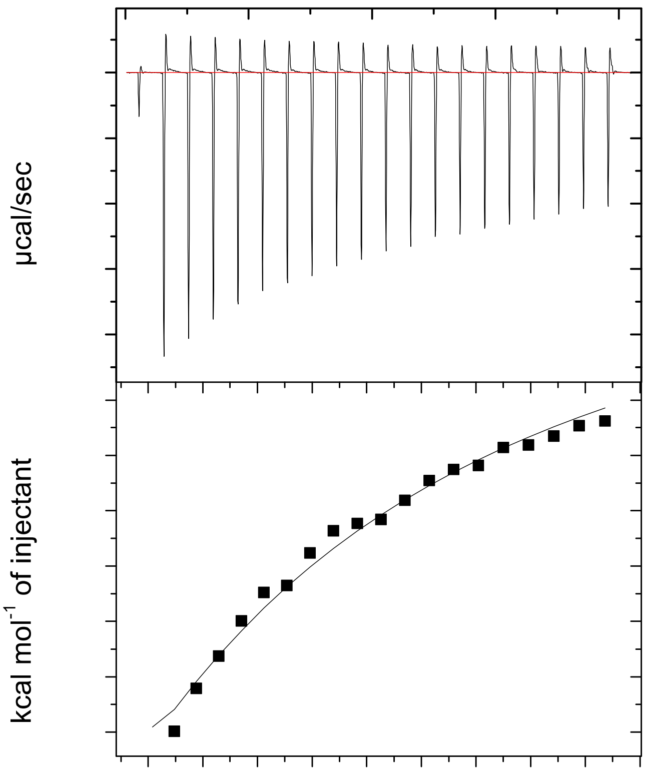

Figure S51. ITC experiments of cage **1-Co^{II}**. Titration plots (heat flow versus time and heat/mol versus guest/host ratio obtained by titrating the solution of the cage (3 mM) with pyrene guest (30 mM) in acetonitrile. The line represents the best-fit line resulting from fitting by using the sequential binding sites model.

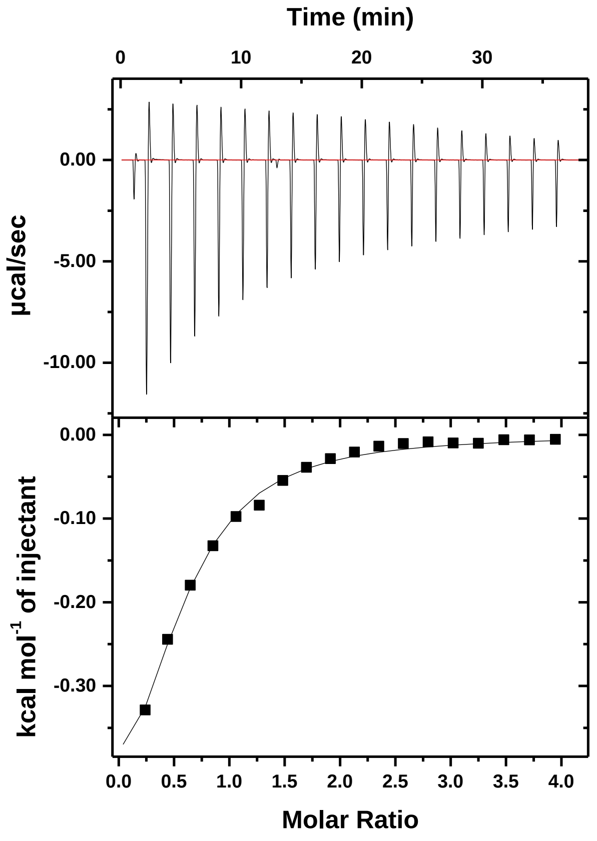

Figure S52. ITC experiments for cage **1-Co^{II}**. Titration plots (heat flow versus time and heat/mol versus guest/host ratio obtained by titrating a solution of the cage (5 mM) with the phenanthrene guest (100 mM) in acetonitrile. The line represents the best fit resulting from fitting using the one set of sites model.

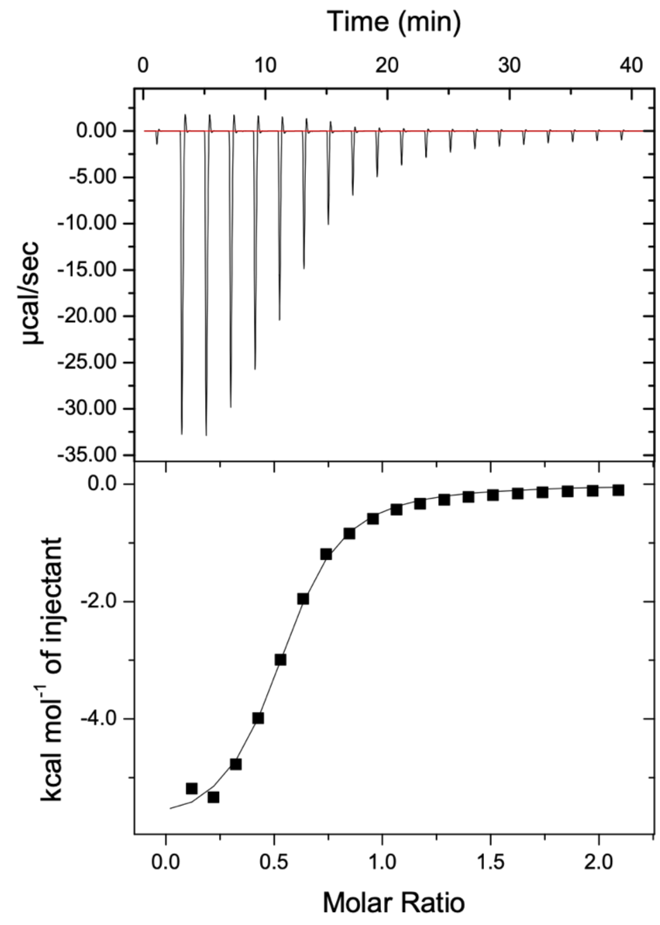

Figure S53. ITC experiments of cage **1-Co^{II}**. Titration plots (heat flow versus time and heat/mol versus guest/host ratio obtained by titrating the solution of the cage (3 mM) with *β*-endosulfan guest (30 mM) in acetonitrile. The line represents the best-fit line resulting from fitting by using the one set of sites model.

S3.3. Titration plots for host 2-Co^{II}

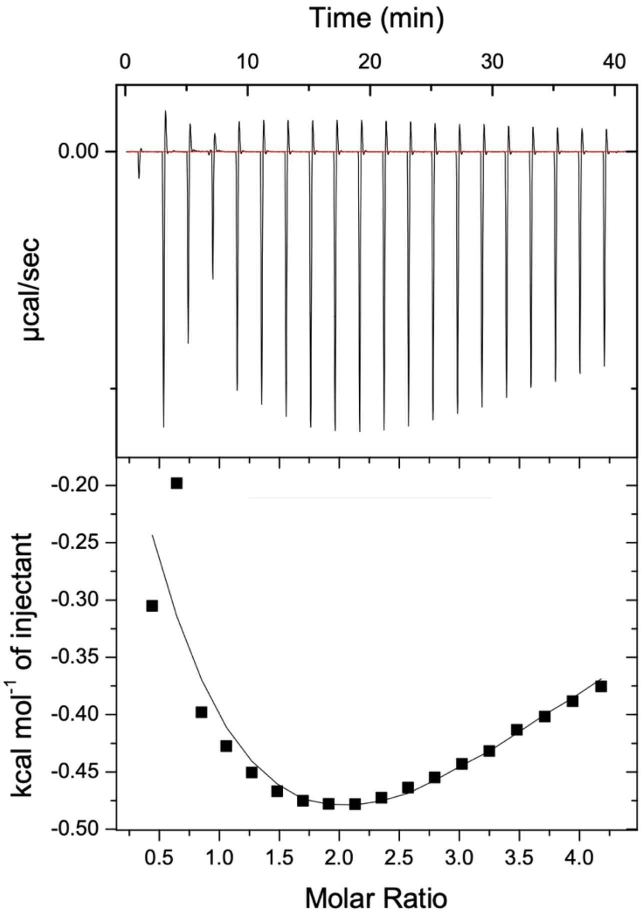

Figure S54. ITC experiments of cage **2-Co^{II}**. Titration plots (heat flow versus time and heat/mol versus guest/host ratio obtained by titrating the solution of the cage (1 mM) with pyrene guest (20 mM) in acetonitrile. The line represents the best-fit line resulting from fitting by using the sequential binding sites model.

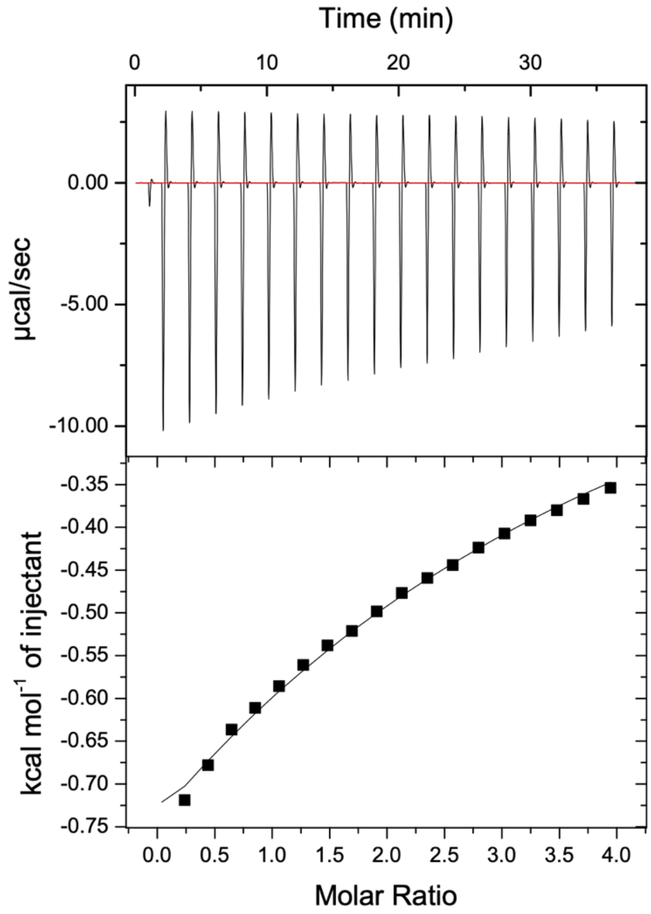

Figure S55. ITC experiments of cage **2-Co^{II}**. Titration plots (heat flow versus time and heat/mol versus guest/host ratio obtained by titrating the solution of the cage (1 mM) with phenanthrene guest (10 mM) in acetonitrile. The line represents the best-fit line resulting from fitting by using the one set of sites model.

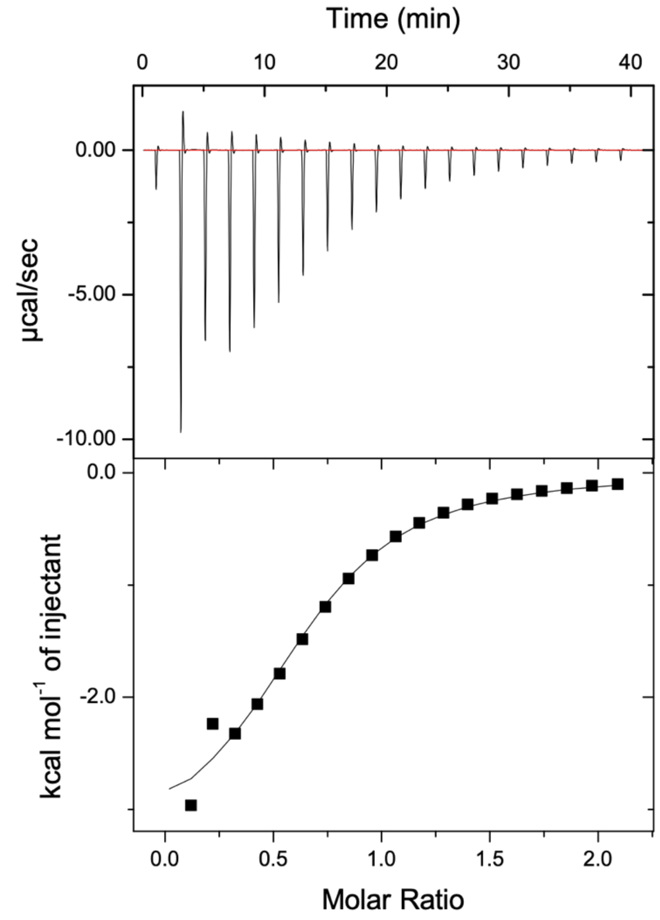

Figure S56. ITC experiments of cage **2-Co^{II}**. Titration plots (heat flow versus time and heat/mol versus guest/host ratio obtained by titrating the solution of the cage (1 mM) with *β*-endosulfan guest (10 mM) in acetonitrile. The line represents the best-fit line resulting from fitting by using the one set of sites model.

S3.4. Titration plots for host 3-Co^{II}

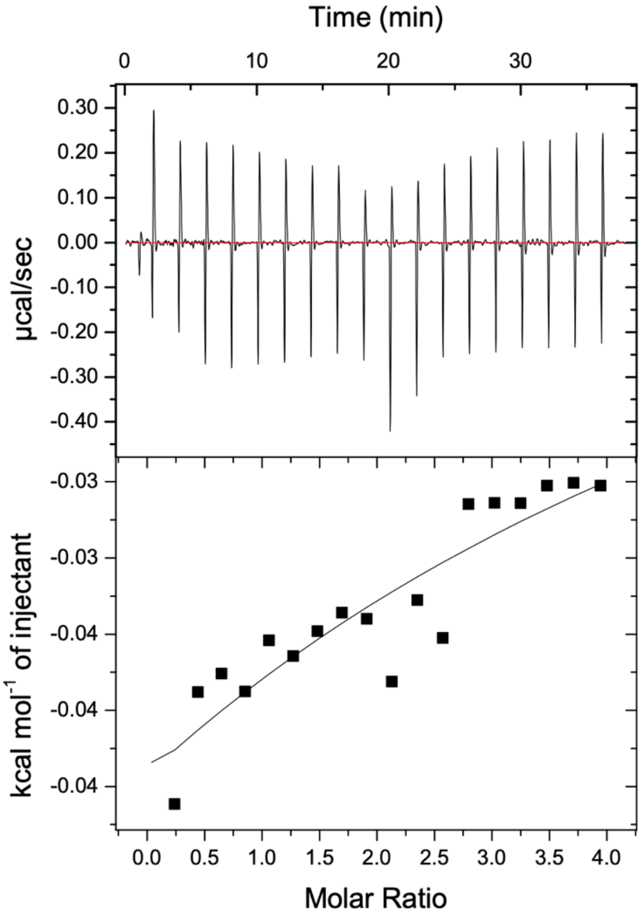

Figure S57. ITC experiments of cage **3-Co^{II}**. Titration plots (heat flow versus time and heat/mol versus guest/host ratio obtained by titrating the solution of the cage (3 mM) with pyrene guest (30 mM) in acetonitrile. The line represents the best-fit line resulting from fitting by using the one set of sites model. **3-Co^{II}** exhibited very weak binding to pyrene, which was also confirmed by ^{1}H NMR data in Figure S64, thus leading to the poor fittings for the ITC titration.

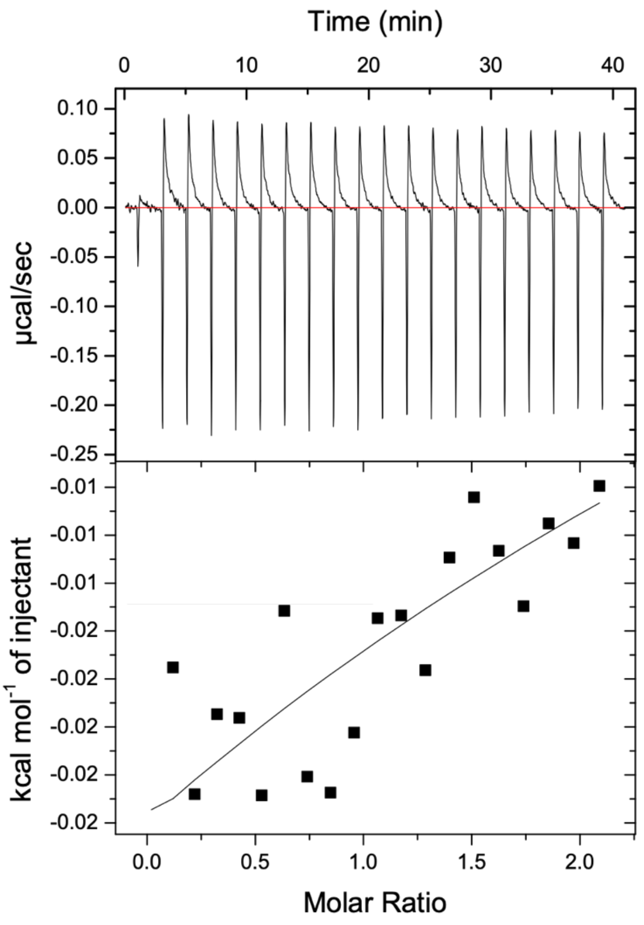

Figure S58. ITC experiments of cage **3-Co^{II}**. Titration plots (heat flow versus time and heat/mol versus guest/host ratio obtained by titrating the solution of the cage (3 mM) with phenanthrene guest (30 mM) in acetonitrile. The line represents the best-fit line resulting from fitting by using the one set of sites model. **3-Co^{II}** exhibited very weak binding to phenanthrene, which was also confirmed by ^{1}H NMR data in Figure S63, thus leading to the poor fittings for the ITC titration.

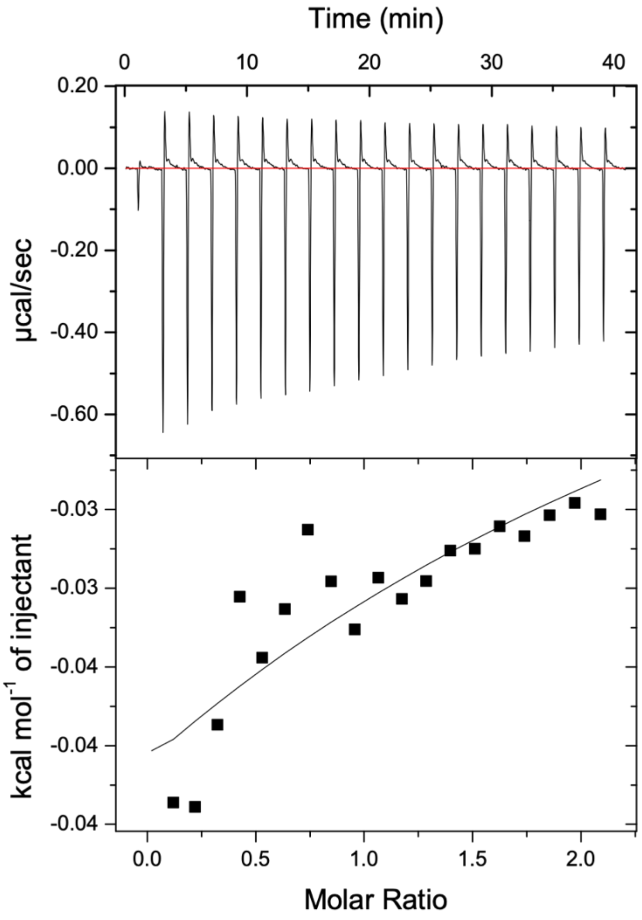

Figure S59. ITC experiments of cage **3-Co^{II}**. Titration plots (heat flow versus time and heat/mol versus guest/host ratio obtained by titrating the solution of the cage (3 mM) with *β*-endosulfan guest (30 mM) in acetonitrile. The line represents the best-fit line resulting from fitting by using the one set of sites model. **3-Co^{II}** exhibited very weak binding to *β*-endosulfan, which was also confirmed by ^{1}H NMR data in Figure S65, thus leading to the poor fittings for the ITC titration.

S3.5. Titration plots for host 4-Co^{II}

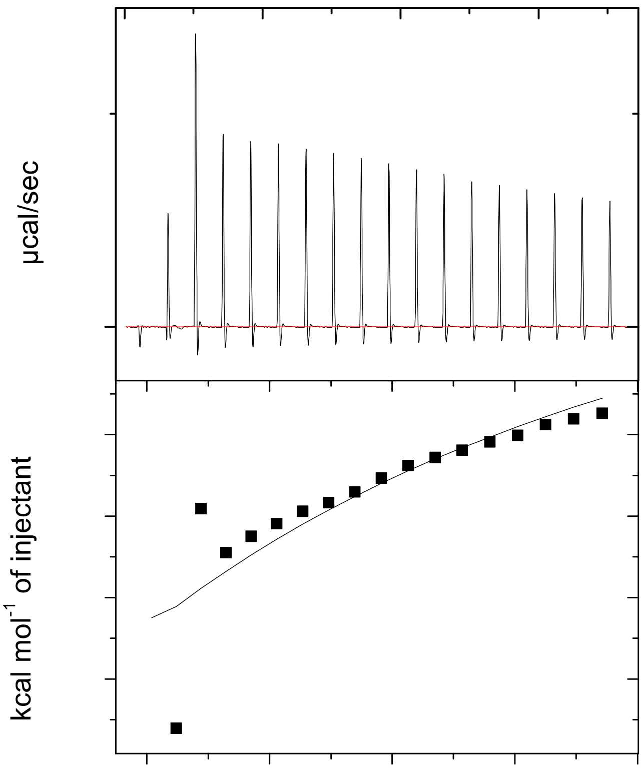

Figure S60. ITC experiments of cage **4-Co^{II}**. Titration plots (heat flow versus time and heat/mol versus guest/host ratio obtained by titrating the solution of the cage (3 mM) with pyrene guest (30 mM) in acetonitrile. The line represents the best-fit line resulting from fitting by using the one set of sites model.

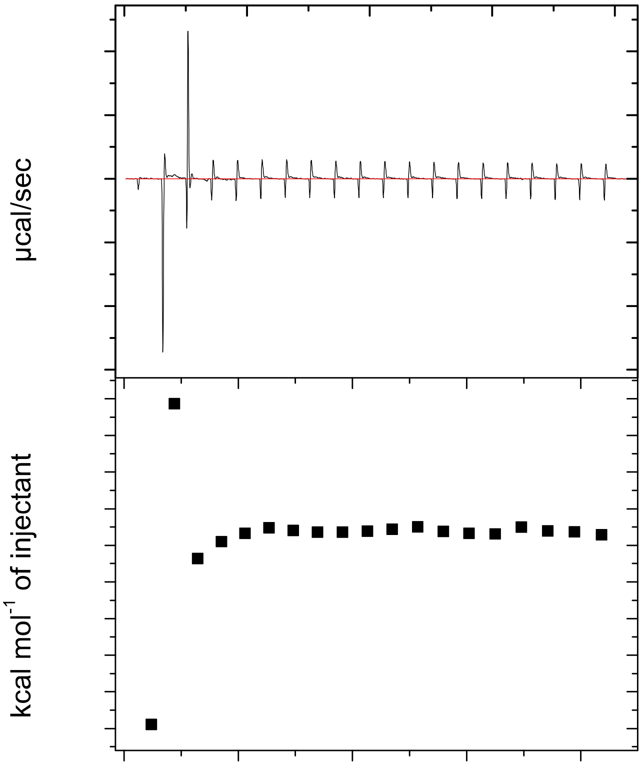

Figure S61. ITC experiments of cage **4-Co**^{II}. Titration plots (heat flow versus time and heat/mol versus guest/host ratio obtained by titrating the solution of the cage (3 mM) with phenanthrene guest (30 mM) in acetonitrile. As no meaningful best-fit line was obtained, it was concluded that the data indicate no binding. The line represents the best-fit line resulting from fitting by using the one set of sites model.

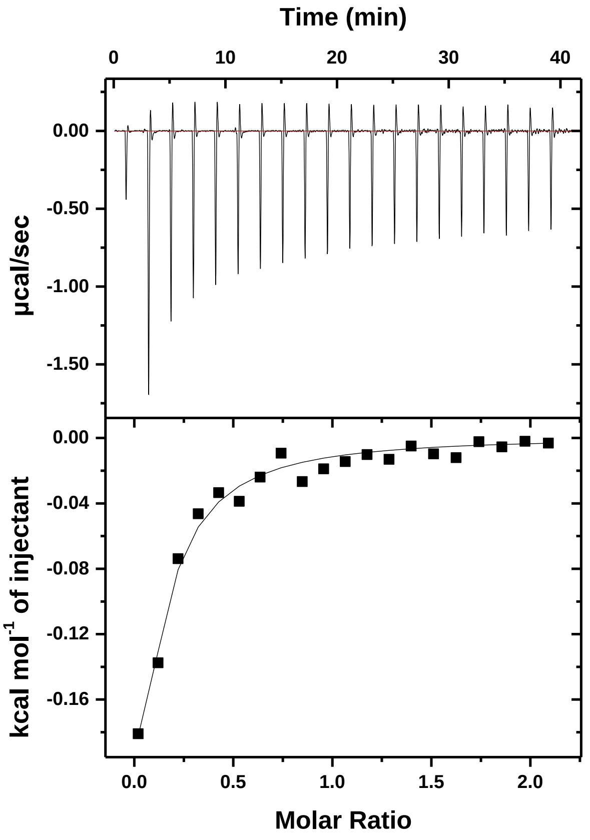

Figure S62. ITC experiments for cage **4-Co^{II}**. Titration plots (heat flow versus time and heat/mol versus guest/host ratio obtained by titrating the solution of the cage (3 mM) with the *β*-endosulfan guest (30 mM) in acetonitrile. The line represents the best fit resulting from fitting using the one set of sites model.

S3.6. Uptake and release of guests

General Method: Cage **1-Co^{II}**, **2-Co^{II}**, **1-Fe^{II}**, or **2-Fe^{II}** was dissolved in CD_3CN (0.5 mM) added to a small glass vial equipped with a stir bar. To the vial was then added a guest (0.5 mM, 1 equiv per assembly for the guests phenanthrene and *β*-endosulfan; 1.0 mM, 2 equiv per assembly for the guest pyrene) and the solution allowed to stir for 18 hours to equilibrate. A 5% solution of methylene blue was then transferred to the vial and the mixture stirred under light irradiation (630 nm) for two hours.

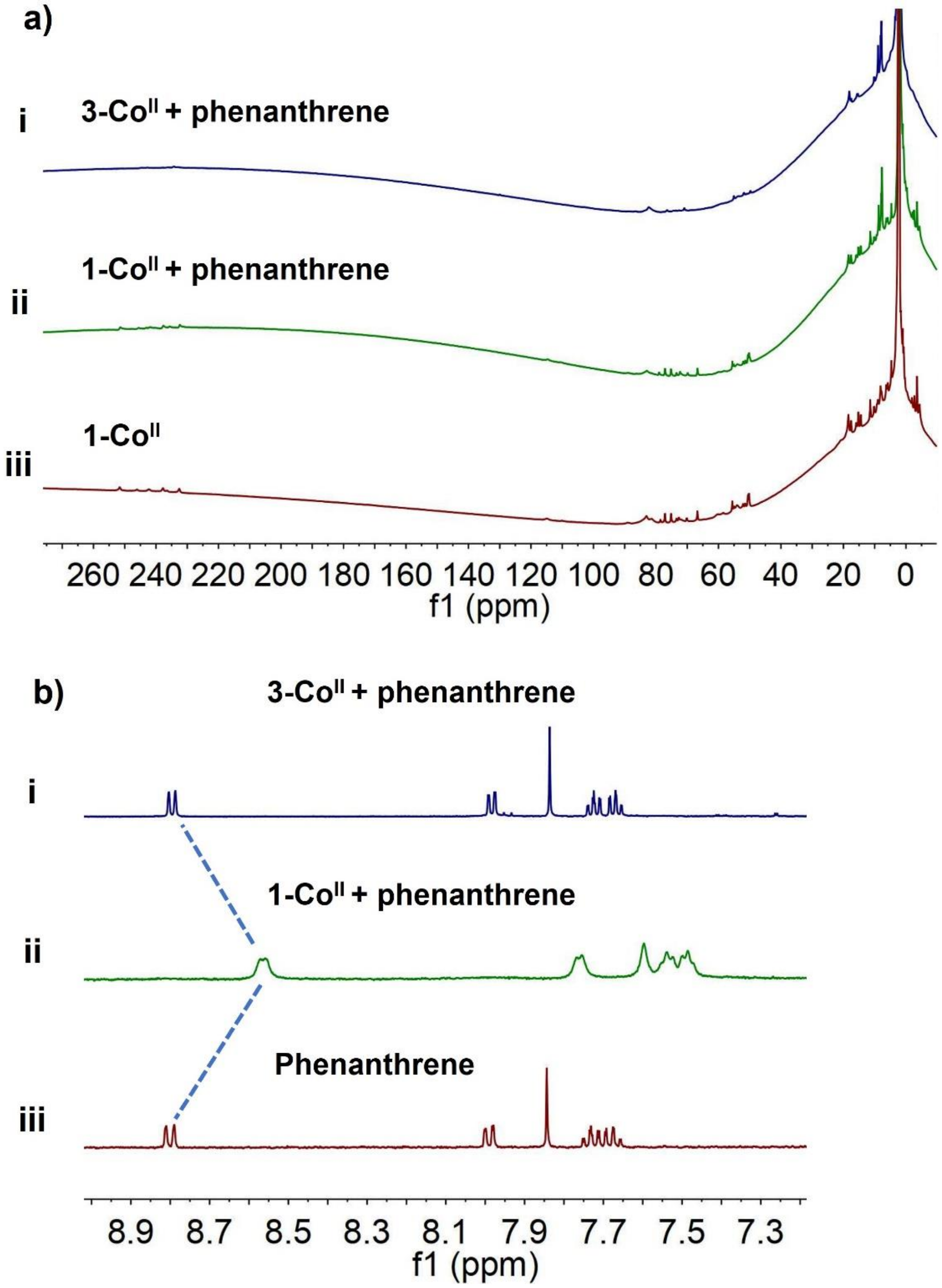

Figure S63. (a) Wide sweep ^{1}H NMR (400 MHz, CD_3CN, 298 K) spectra of i) cage **3-Co^{II}** with the guest phenanthrene; ii) cage **1-Co^{II}** with the guest phenanthrene; iii) cage **1-Co^{II}**. (b) Narrow sweep width ^{1}H NMR (400 MHz, CD_3CN, 298 K) of i) cage **3-Co^{II}** with the guest phenanthrene; ii) cage **1-Co^{II}** with the guest phenanthrene; iii) phenanthrene. After the hetero-Diels-Alder reaction with $^{1}O_2$ to produce **3-Co^{II}**, no further evidence for phenanthrene binding was observed, consistent with its release.

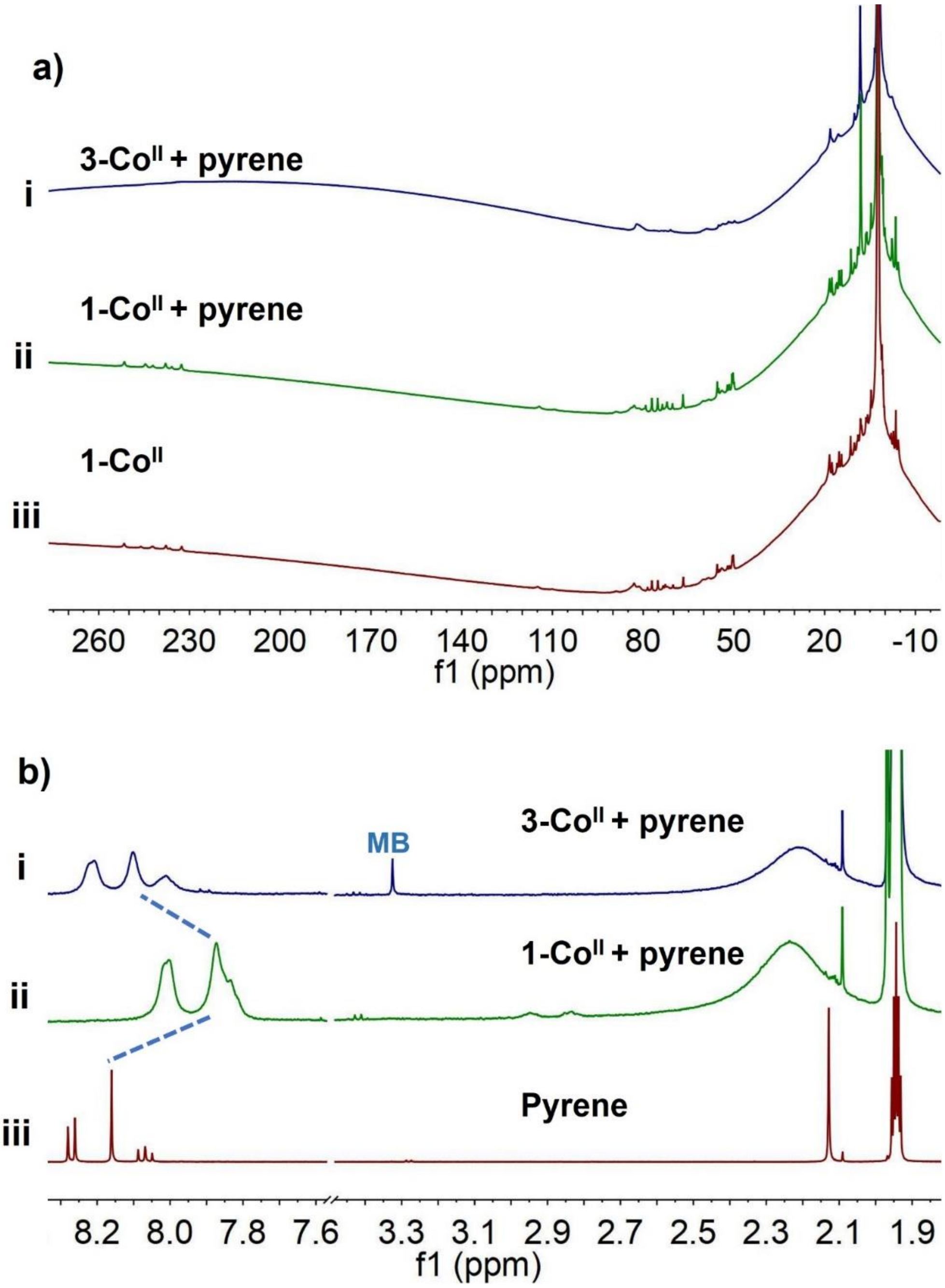

Figure S64. (a) Wide sweep ^{1}H NMR (400 MHz, CD_3CN, 298 K) spectra of i) cage **3-Co^{II}** with the guest pyrene; ii) cage **1-Co^{II}** with the guest pyrene; iii) cage **1-Co^{II}.** (b) Narrow sweep width ^{1}H NMR (400 MHz, CD_3CN, 298 K) of i) cage **3-Co^{II}** with the guest pyrene; ii) cage **1-Co^{II}** with the guest pyrene; iii) pyrene. After the hetero-Diels-Alder reaction with $^1O_2$ to produce **3-Co^{II}**, weaker binding with the guest pyrene was observed than in the case of **1-Co^{II}**, resulting in pyrene being partially released.

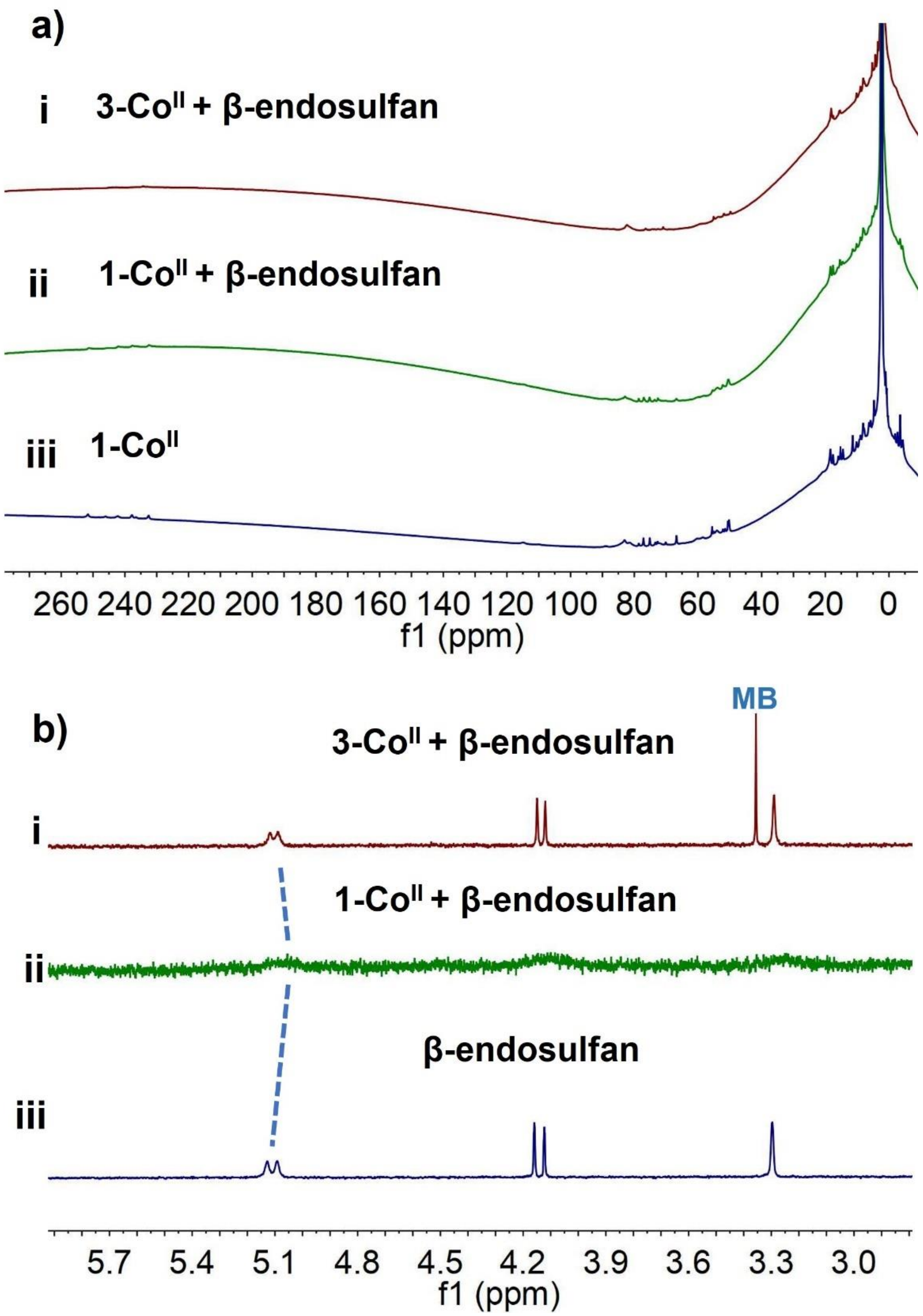

Figure S65. (a) Wide sweep ^{1}H NMR (400 MHz, CD_3CN, 298 K) spectra of i) cage **3-Co^{II}** with the guest *β*-endosulfan; ii) cage **1-Co^{II}** with the guest *β*-endosulfan; iii) cage **1-Co^{II}**. (b) Narrow sweep width ^{1}H NMR (400 MHz, CD_3CN, 298 K) of i) cage **3-Co^{II}** with the guest *β*-endosulfan; ii) cage **1-Co^{II}** with the guest *β*-endosulfan; iii) *β*-endosulfan. After the hetero-Diels-Alder reaction with $^1O_2$ to produce **3-Co^{II}**, no further evidence for *β*-endosulfan binding was observed, consistent with its release.

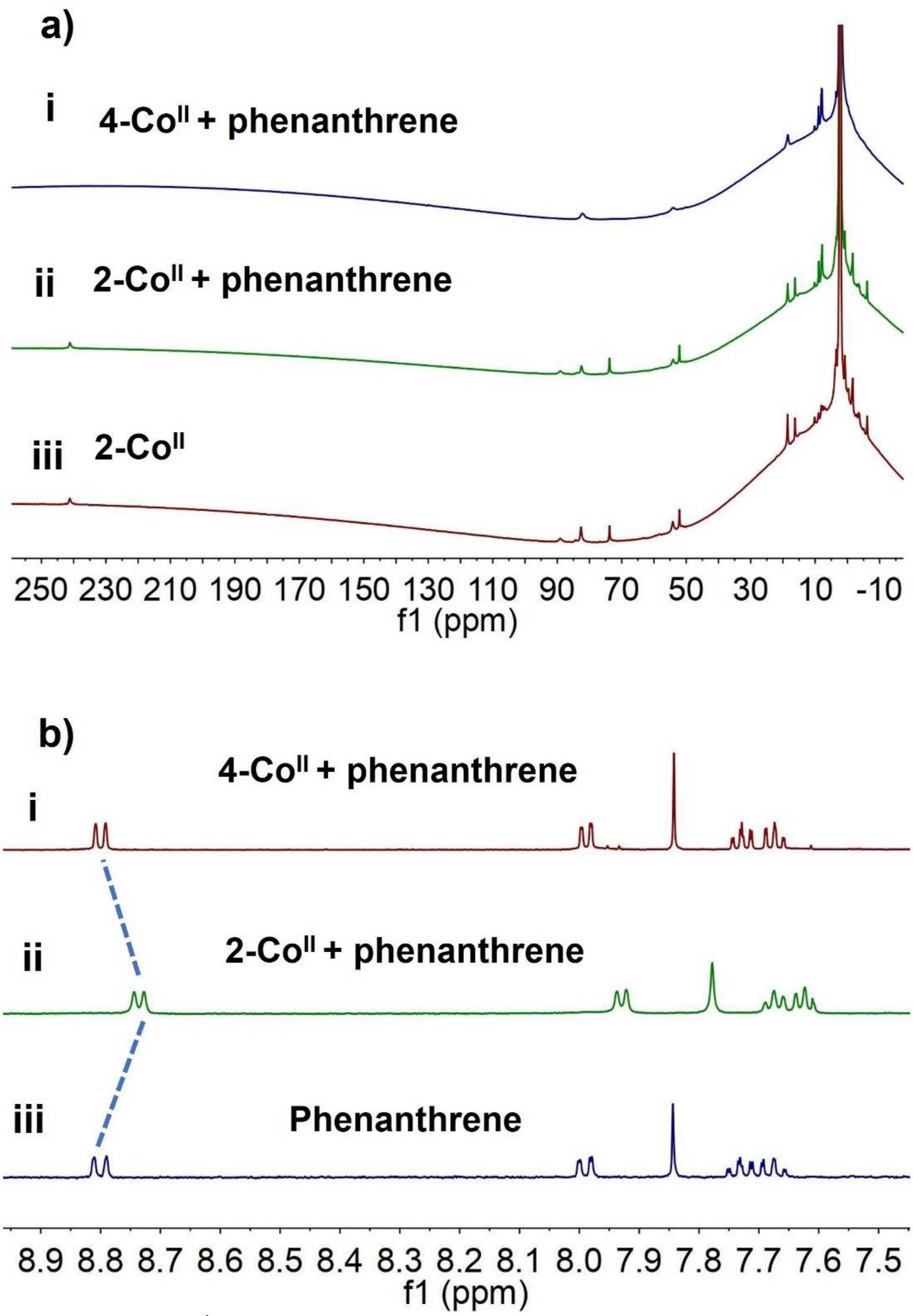

Figure S66. (a) Wide sweep ^{1}H NMR (400 MHz, CD_3CN, 298 K) spectra of i) cage **4-Co^{II}** with the guest phenanthrene; ii) cage **2-Co^{II}** with the guest phenanthrene; iii) cage **2-Co^{II}**. (b) Narrow sweep width ^{1}H NMR (400 MHz, CD_3CN, 298 K) of i) cage **4-Co^{II}** with the guest phenanthrene; ii) cage **2-Co^{II}** with the guest phenanthrene; iii) phenanthrene. After the hetero-Diels-Alder reaction with $^{1}O_2$ to produce **4-Co^{II}**, no further evidence for phenanthrene binding was observed, consistent with its release.

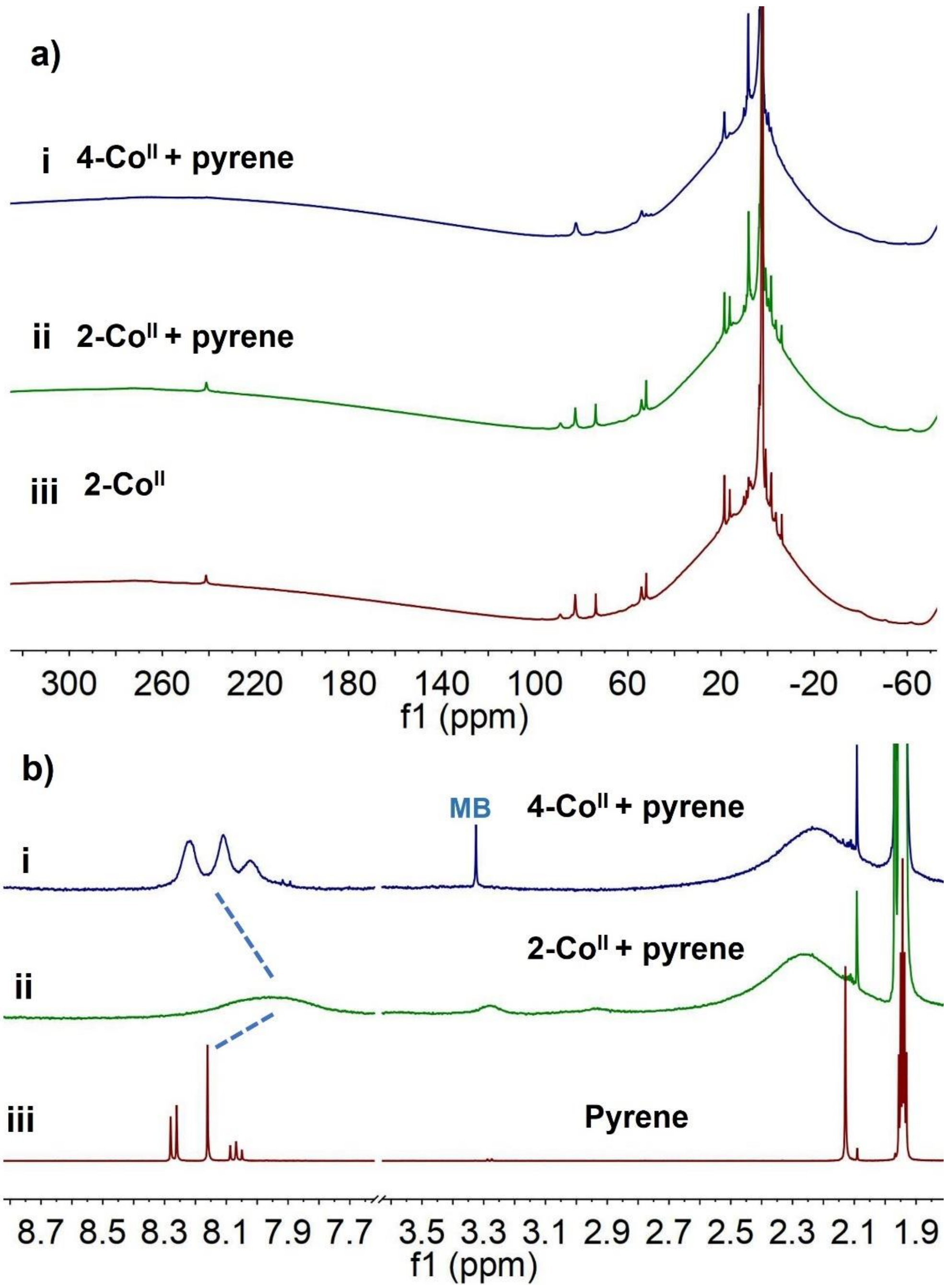

Figure S67. (a) Wide sweep ^{1}H NMR (400 MHz, CD_3CN, 298 K) spectra of i) cage **4-Co^{II}** with the guest pyrene; ii) cage **2-Co^{II}** with the guest pyrene; iii) cage **2-Co^{II}.** (b) Narrow sweep width ^{1}H NMR (400 MHz, CD_3CN, 298 K) of i) cage **4-Co^{II}** with the guest pyrene; ii) cage **2-Co^{II}** with the guest pyrene; iii) pyrene. After the hetero-Diels-Alder reaction with $^1O_2$ to produce **4-Co^{II}**, weaker binding with the guest pyrene was observed than in the case of **2-Co^{II}**, resulting in pyrene being partially released.

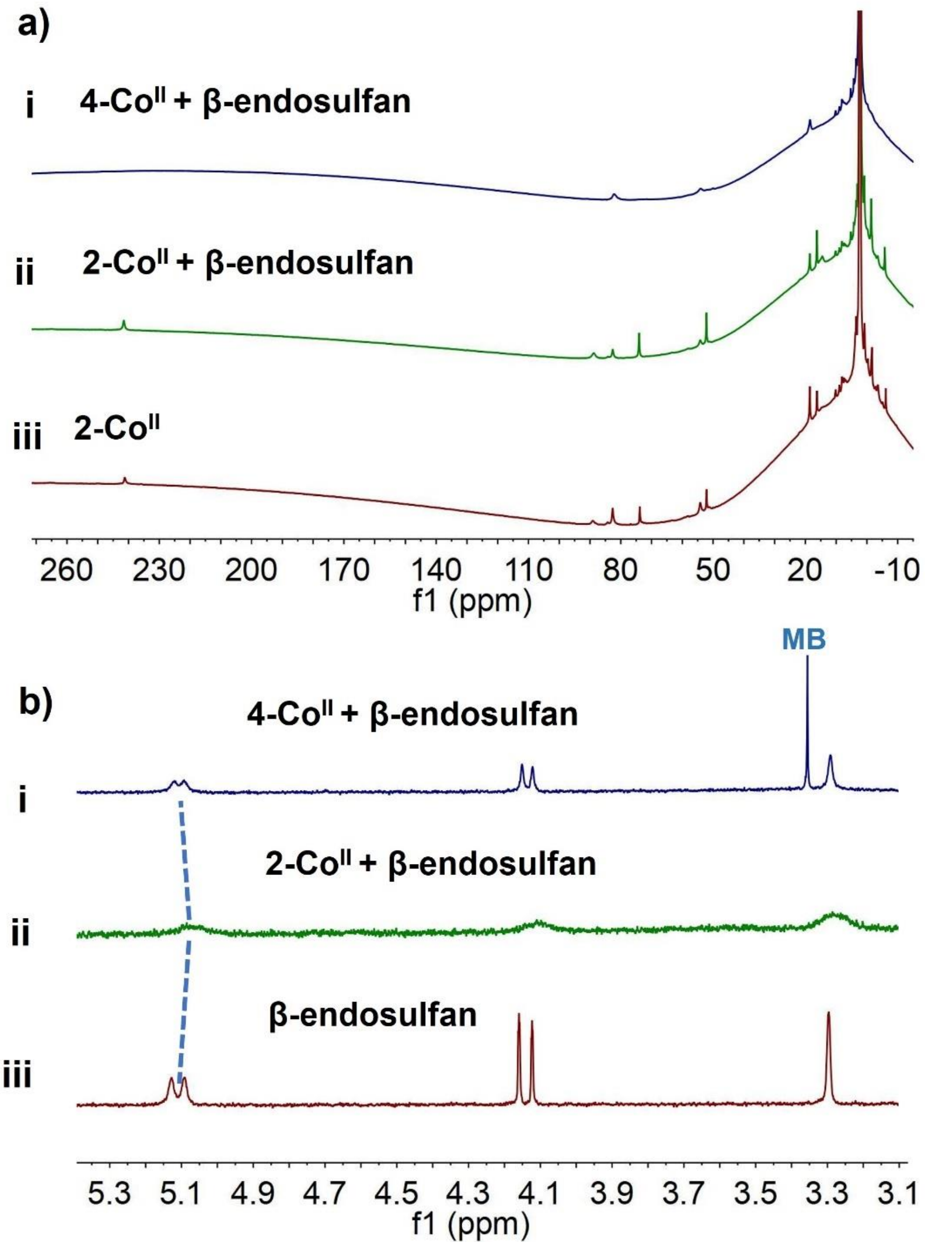

Figure S68. (a) Wide sweep ^{1}H NMR (400 MHz, CD_3CN, 298 K) spectra of i) cage **4-Co^{II}** with the guest *β*-endosulfan; ii) cage **2-Co^{II}** with the guest *β*-endosulfan; iii) cage **2-Co^{II}**. (b) Narrow sweep width ^{1}H NMR (400 MHz, CD_3CN, 298 K) of i) cage **4-Co^{II}** with the guest *β*-endosulfan; ii) cage **2-Co^{II}** with the guest *β*-endosulfan; iii) *β*-endosulfan. After the hetero-Diels-Alder reaction with $^1O_2$ to produce **4-Co^{II}**, no further evidence for *β*-endosulfan binding was observed, consistent with its release.

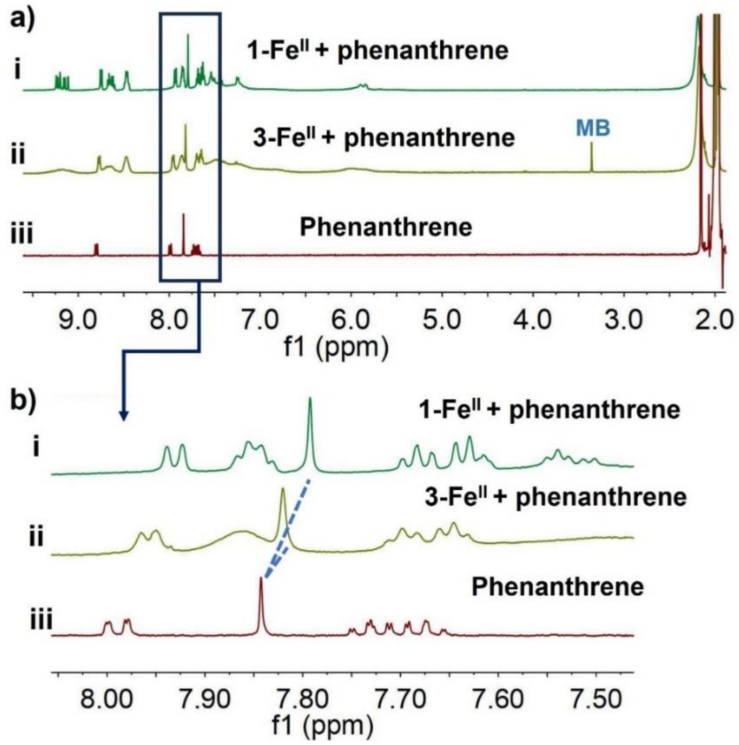

Figure S69. (a) ^{1}H NMR (400 MHz, CD_3CN, 298 K) spectra of i) cage **1-Fe^{II}** with the guest phenanthrene; ii) cage **3-Fe^{II}** with the guest phenanthrene; iii) phenanthrene. (b) Partial ^{1}H NMR (400 MHz, CD_3CN, 298 K) spectra of (a). After the hetero-Diels-Alder reaction with $^{1}O_2$ to produce **3-Fe^{II}**, weaker binding with the guest phenanthrene was observed than in the case of **1-Fe^{II}**, resulting in phenanthrene being partially released.

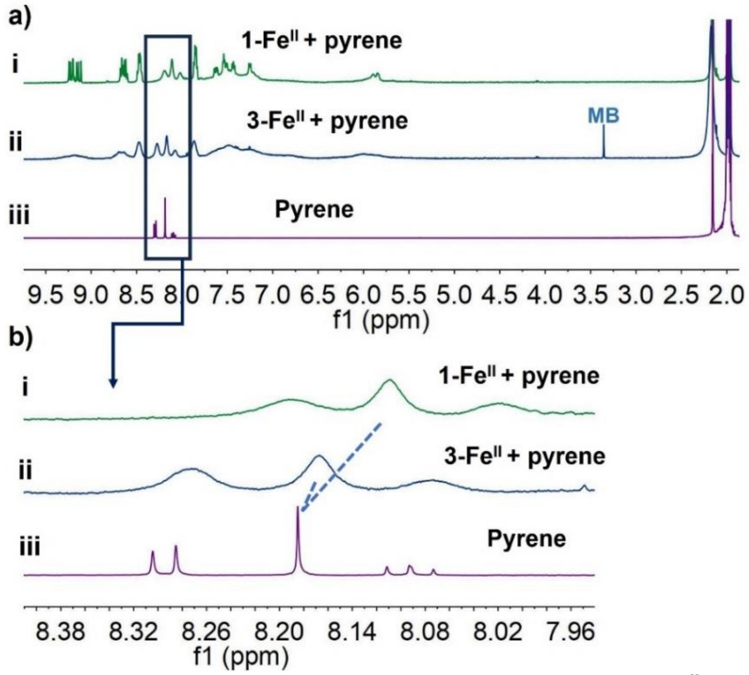

Figure S70. (a) ^{1}H NMR (400 MHz, CD_3CN, 298 K) spectra of i) cage **1-Fe^{II}** with the guest pyrene; ii) cage **3-Fe^{II}** with the guest pyrene; iii) pyrene. (b) Partial ^{1}H NMR (400 MHz, CD_3CN, 298 K) spectra of (a). After the hetero-Diels-Alder reaction with $^{1}O_2$ to produce **3-Fe^{II}**, weaker binding with the guest pyrene was observed than in the case of **1-Fe^{II}**, resulting in pyrene being partially released.

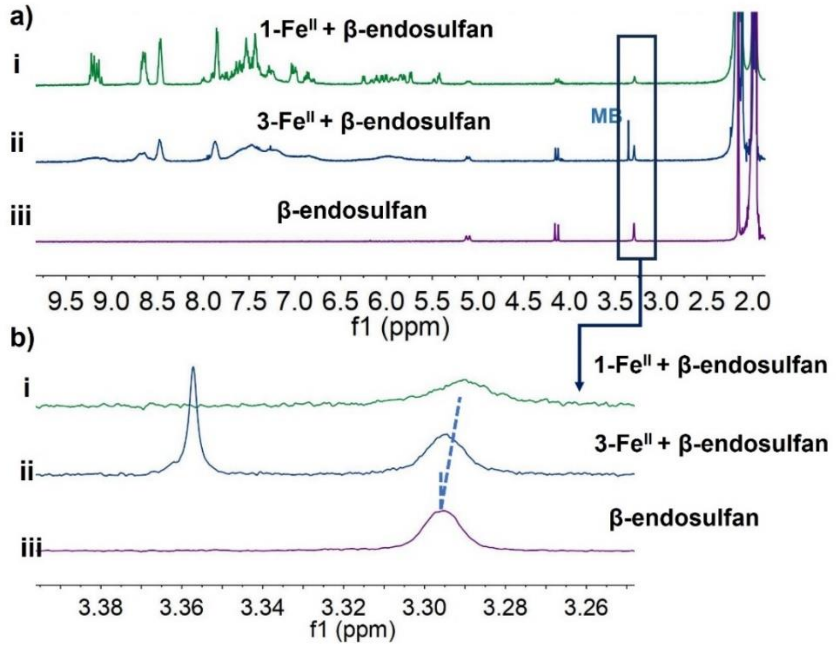

Figure S71. (a) ^{1}H NMR (400 MHz, CD_3CN, 298 K) spectra of i) cage **1-Fe^{II}** with the guest *β*-endosulfan; ii) cage **3-Fe^{II}** with the guest *β*-endosulfan; iii) *β*-endosulfan. (b) Partial ^{1}H NMR (400 MHz, CD_3CN, 298 K) spectra of (a). After the hetero-Diels-Alder reaction with $^1O_2$ to produce **3-Fe^{II}**, weaker binding with the guest *β*-endosulfan was observed than in the case of **1-Fe^{II}**, resulting in *β*-endosulfan being partially released.

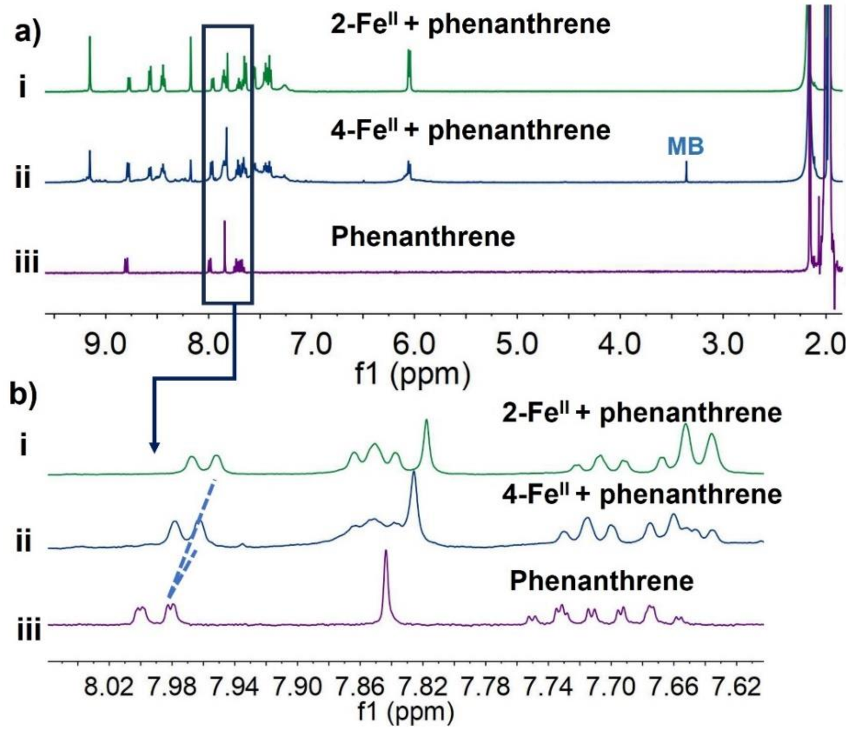

Figure S72. (a) ^{1}H NMR (400 MHz, CD_3CN, 298 K) spectra of i) cage **2-Fe^{II}** with the guest phenanthrene; ii) cage **4-Fe^{II}** with the guest phenanthrene; iii) phenanthrene. (b) Partial ^{1}H NMR (400 MHz, CD_3CN, 298 K) spectra of (a). After the hetero-Diels-Alder reaction with $^1O_2$ to produce **4-Fe^{II}**, weaker binding with the guest phenanthrene was observed than in the case of **2-Fe^{II}**, resulting in phenanthrene being partially released.

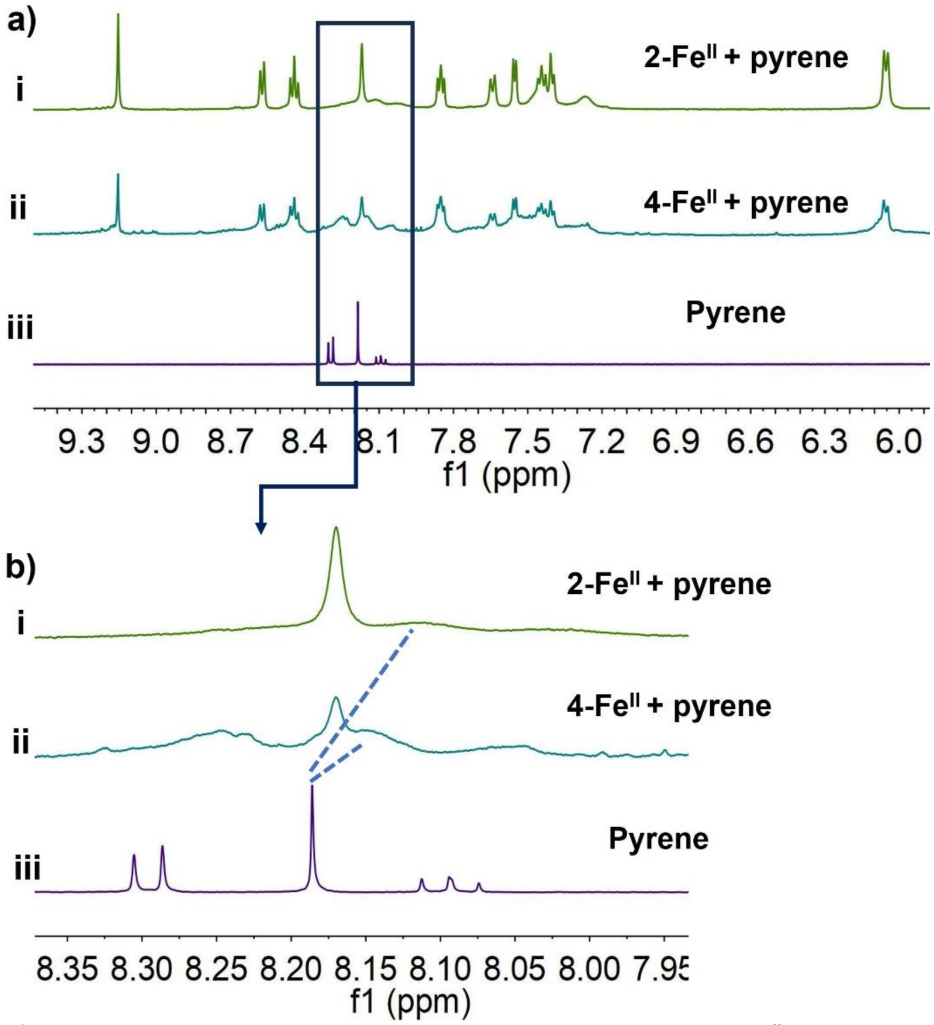

Figure S73. (a) ^{1}H NMR (400 MHz, CD_3CN, 298 K) spectra of i) cage **2-Fe^{II}** with the guest pyrene; ii) cage **4-Fe^{II}** with the guest pyrene; iii) pyrene. (b) Partial ^{1}H NMR (400 MHz, CD_3CN, 298 K) spectra of (a). After the hetero-Diels-Alder reaction with $^{1}O_2$ to produce **4-Fe^{II}**, weaker binding with the guest pyrene was observed than in the case of **2-Fe^{II}**, resulting in pyrene being partially released.

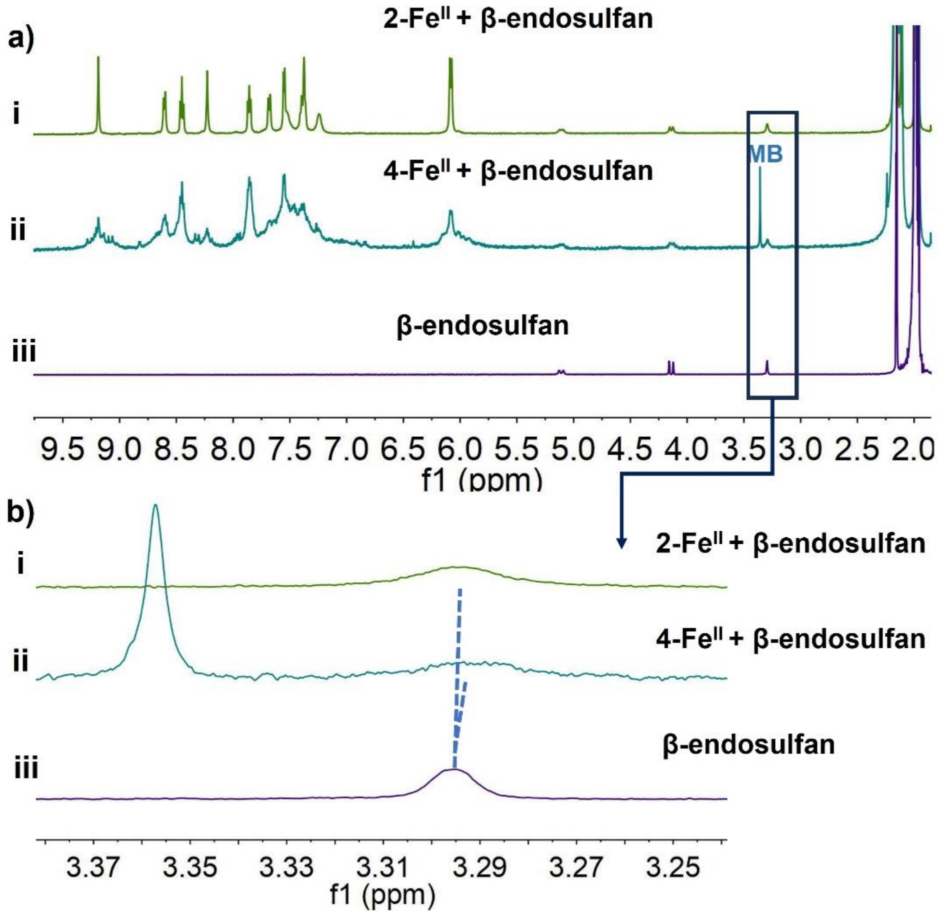

Figure S74. (a) ^{1}H NMR (400 MHz, CD_3CN, 298 K) spectra of i) cage **2-Fe^{II}** with guest *β*-endosulfan; ii) cage **4-Fe^{II}** with the guest *β*-endosulfan; iii) *β*-endosulfan. (b) Partial ^{1}H NMR (400 MHz, CD_3CN, 298 K) spectra of (a). After the hetero-Diels-Alder reaction with $^1O_2$ to produce **4-Fe^{II}**, the ^{1}H NMR signals of the guest *β*-endosulfan did not exhibit clear shifts, consistent with *β*-endosulfan still being encapsulated.

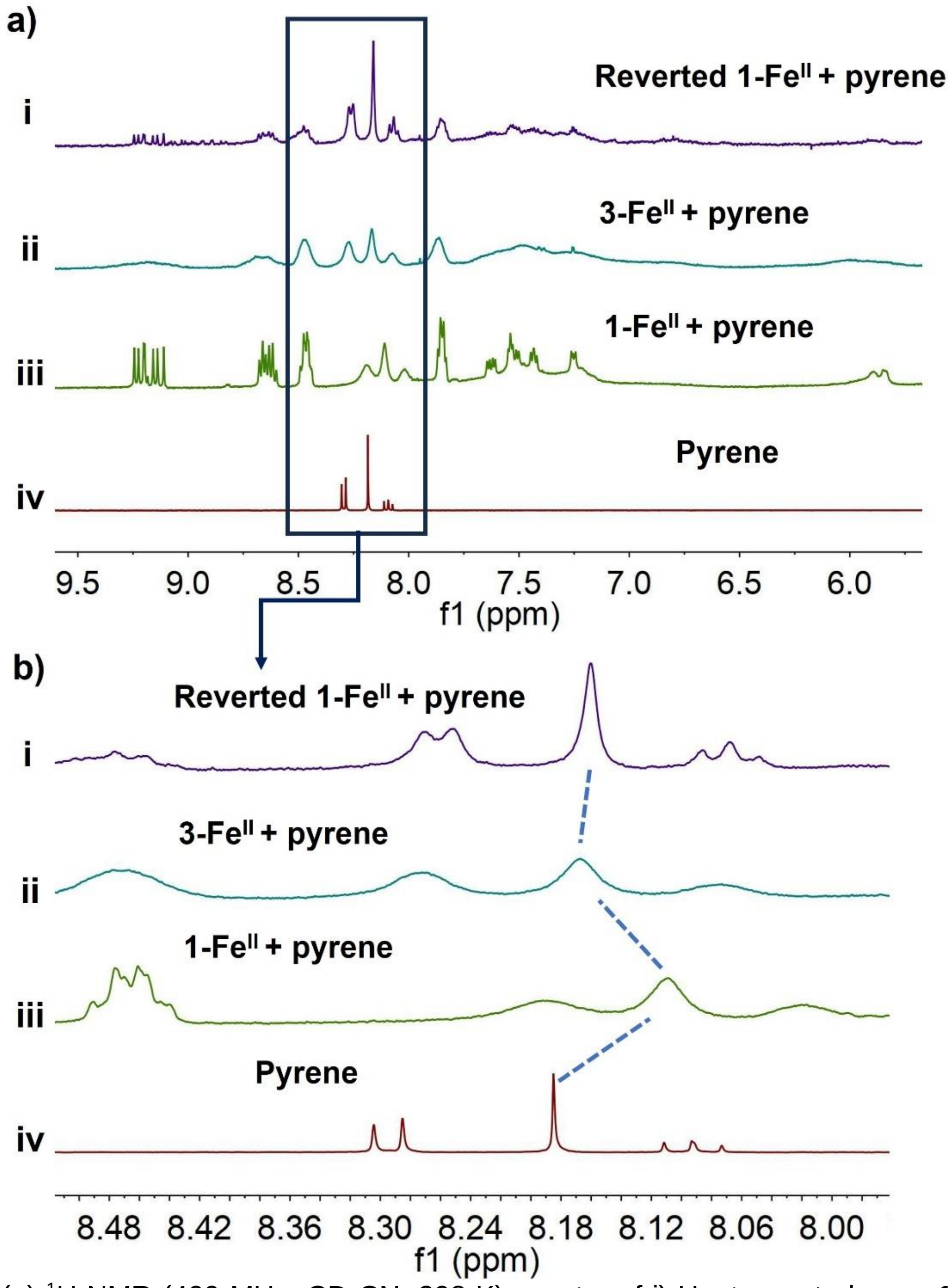

Figure S75. (a) ^{1}H NMR (400 MHz, CD_3CN, 298 K) spectra of i) Heat reverted cage **1-Fe^{II}** with the guest pyrene; ii) cage **3-Fe^{II}** with the guest pyrene; iii) cage **1-Fe^{II}** with the guest pyrene; iv) pyrene. (b) Partial ^{1}H NMR (400 MHz, CD_3CN, 298 K) spectra of (a). After heating, the reversible hetero-Diels-Alder reaction partially reverted to produce the parent cage **1-Fe^{II}**, which exhibits host-guest binding with pyrene, leading to the reversible encapsulation of this guest.

S4. References

(1) Wiseman, T.; Williston, S.; Brandts, J. F.; Lin, L. N. *Anal. Biochem.* **1989**, *179*, 131–137.

(2) Ronson, T. K.; Meng, W.; Nitschke, J. R. *J. Am. Chem. Soc.* **2017**, *139*, 9698–9707.

(3) Mizoue, L. S.; Tellinghuisen, J. *Anal. Biochem.* **2004**, *326*, 125–127.

(4) Ronson, T. K.; Pilgrim, B. S.; Nitschke, J. R. *J. Am. Chem. Soc.* **2016**, *138*, 10417–10420.

(5) Ronson, T. K.; Wang, Y.; Baldridge, K.; Siegel, J. S.; Nitschke, J. R. *J. Am. Chem. Soc.* **2020**, *142*, 10267–10272.
